# CRISPR/Cas9-based toolkit for rapid marker recycling and combinatorial libraries in *Komagataella phaffii*

**DOI:** 10.1007/s00253-024-13037-1

**Published:** 2024-02-07

**Authors:** Wei Zhou, Yuanyi Li, Guosong Liu, Weichuang Qin, Dongzhi Wei, Fengqing Wang, Bei Gao

**Affiliations:** State Key Laboratory of Bioreactor Engineering, New World Institute of Biotechnology, East China University of Science and Technology, 130 Meilong Road, P.O.B.311, Shanghai, 200237 China

**Keywords:** *Komagataella phaffii*, CRISPR/Cas9, Visual marker recycling, Multi-fragment integration, Large DNA fragment knockout, Yeast assembled libraries

## Abstract

**Abstract:**

*Komagataella phaffii*, a nonconventional yeast, is increasingly attractive to researchers owing to its posttranslational modification ability, strict methanol regulatory mechanism, and lack of Crabtree effect. Although CRISPR-based gene editing systems have been established in *K. phaffii*, there are still some inadequacies compared to the model organism *Saccharomyces cerevisiae*. In this study, a redesigned gRNA plasmid carrying red and green fluorescent proteins facilitated plasmid construction and marker recycling, respectively, making marker recycling more convenient and reliable. Subsequently, based on the knockdown of *Ku70* and *DNA ligase IV*, we experimented with integrating multiple DNA fragments at a single locus. A 26.5-kb-long DNA fragment divided into 11 expression cassettes for lycopene synthesis could be successfully integrated into a single locus at one time with a success rate of 57%. A 27-kb-long DNA fragment could also be precisely knocked out with a 50% positive rate in *K. phaffii* by introducing two DSBs simultaneously. Finally, to explore the feasibility of rapidly balancing the expression intensity of multiple genes in a metabolic pathway, a yeast combinatorial library was successfully constructed in *K. phaffii* using lycopene as an indicator, and an optimal combination of the metabolic pathway was identified by screening, with a yield titer of up to 182.73 mg/L in shake flask fermentation.

**Key points:**

*• Rapid marker recycling based on the visualization of a green fluorescent protein*

*• One-step multifragment integration and large fragment knockout in the genome*

*• A random assembly of multiple DNA elements to create yeast libraries in K. phaffii*

**Graphical Abstract:**

**Figure:**
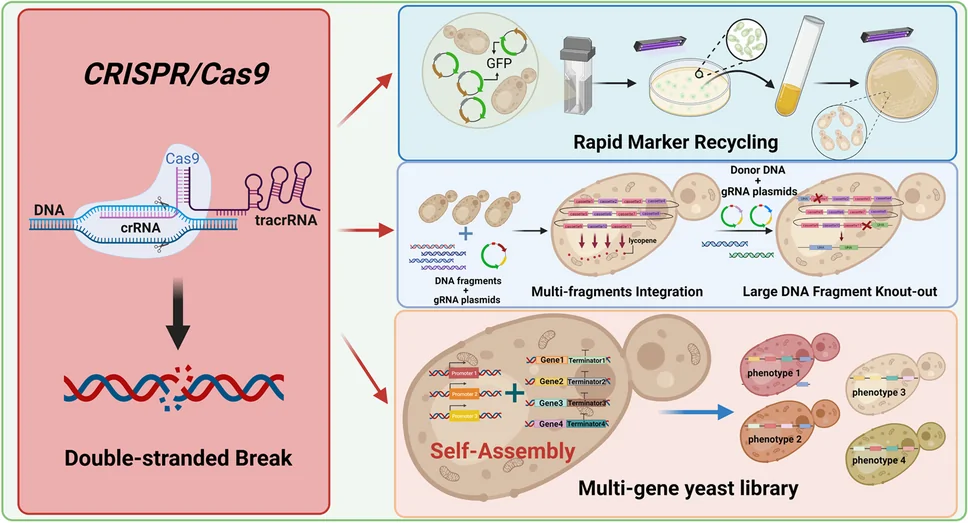

**Supplementary Information:**

The online version contains supplementary material available at 10.1007/s00253-024-13037-1.

## Introduction

With the advantages of mild reaction conditions and high reaction specificity, a vast number of microbial cell factories have been constructed in recent years to synthesize a variety of bulk chemicals through synthetic biology and metabolic engineering. Yeast, as a typical eukaryotic microorganism, plays an important role in the microbial cell factory; for example, Liu et al. (2020) achieved the compartmentalized synthesis of squalene in the peroxisome organelle of *Saccharomyces cerevisiae*. Among various yeasts, *K. phaffii* is a well-established GRAS (generally recognized as safe) eukaryotic host that is typically used for the expression and production of heterologous proteins due to its excellent posttranslational modification ability and tight methanol regulation mechanism (Ahmad et al. 2014; Yang and Zhang 2018). In contrast to *S. cerevisiae*, *K. phaffii* is a methylotrophic Crabtree-negative yeast and accordingly produces almost no ethanol as a byproduct during glucose fermentation. Additionally, *K. phaffii* could achieve high-density fermentation in basic media with methanol as the only carbon source to maintain cell growth and synthesize products. And the peroxisome organelles of *K. phaffii* could proliferate and expand under methanol-induced conditions (Bernauer et al. 2021; Ohsawa et al. 2022). Therefore, an increasing number of researchers have focused on yeast engineering to construct cellular factories of *K. phaffii* (Guo et al. 2021; Jin et al. 2021; Zuo et al. 2022).

Previously, systems for the stable expression and secretion of heterologous proteins in *K. phaffii* were developed mainly depending on single crossover, which was also applied in the biosynthesis of secondary metabolites. For instance, Jin et al. (2021) reported the efficient synthesis of nonanimal chondroitin sulfate using a linearized plasmid insertion approach. However, this method presents limitations in metabolic engineering and regulation of gene circuits, such as the relatively small number of regulatable genes and cumbersome selection marker recovery. Since constructing a well-organized cell factory usually involves the co-regulation of multiple genes, the conventional approach for genomic modification in *K. phaffii* could not comprehensively fulfill the need to manufacture cell factories. Thus, more efficient gene-editing techniques are desired.

Due to its simplicity, efficiency, and precision, CRISPR/Cas9 has been applied in the modification of *K. phaffii* (Weninger et al. 2015, 2016). For example, metabolic network rearrangement was realized via the CRISPR technique and succeeded in the synthesis of a variety of high-value chemicals, such as malic acid (Guo et al. 2021), α-santalene (Zuo et al. 2022), and fatty acids (Cai et al. 2022). Moreover, single-locus multifragment integration and multilocus editing were developed in *K. phaffii* (Cai et al. 2021; Liu et al. 2019; Otto et al. 2021; Zhang et al. 2021*)*. Although CRISPR-based gene editing systems have been constructed in *K. phaffii*, they still present shortcomings compared to their application in *S. cerevisiae*. For instance, in *S. cerevisiae*, a total of 15 DNA fragments have been knocked in at three loci in one step by CasEMBLR in one transformation (Jakociunas et al. 2015). However, in *K. phaffii*, only up to nine fragments (from three expression cassettes) have been integrated into a single target in one transformation (Nishi et al. 2022). In addition, constructing cell factories generally requires reusable selective markers for iterative transformation. *ScURA3*, with which *S. cerevisiae* is unable to grow on plates containing 5-fluoroorotic acid, could be rapidly recycled through counter-selection (Kotaka et al. 2009; Moon et al. 2022). However, the primary selective markers used in *K. phaffii* are Geneticin (G418) (Che et al. 2020), bleomycin (Zeo) (Shen et al. 2021), and *KpHis4* (Shen et al. 2021; Tan et al. 2011), which are recycled upon ineffective natural loss by culturing colonies in a nonselective medium.

Thus, this study aimed to extend the CRISPR/Cas9 gene-editing tools in *K. phaffii*. First, gRNA plasmids were redesigned with the addition of green fluorescent protein (GFP) to facilitate the screening of positive transformants and visualize the rapid recycling of markers. Multi-fragment integration in one transformation step was then tested, and the design for the integration of 11 DNA fragments at a single locus with a total length of more than 26 kb was performed. In addition, the knockout of a large fragment of more than 27 kb in the genome was carried out by simultaneously designing and importing two gRNAs. Finally, a yeast combinatorial library of a multigene pathway was first designed in *K. phaffii*, and the feasibility of the concept was verified using the lycopene synthesis pathway.

## Materials and methods

### Materials, reagents, medium, and cultivation conditions

*E. coli DH5α* was used as a host for plasmid construction and amplification cultured in LB medium containing 10 g/L tryptone, 5 g/L yeast extract, and 10 g/L NaCl at 37 °C. Ampicillin and kanamycin were added to LB medium for screening *E. coli*-positive transformants, working at 100 mg/L and 50 mg/L, respectively. *K. phaffii CBS7435* (NRRL-Y11430 and ATCC 76273) was used as a chassis strain in this study, cultured in YPD medium containing 10 g/L yeast extract, 20 g/L peptone, and 20 g/L glucose. Genomycin (G418) was added to the YPD for screening positive transformants at a working concentration of 300 mg/L. The primers used in this study (listed in Table S3) were provided by Tsingke (Shanghai, China). KodOne DNA polymerase (TOYOBO, Japan) and PrimeSTAR Max DNA Polymerase (Takara, Japan) were used to amplify DNA fragments. Hieff Clone™ Plus One Step Cloning kit (Yeasen, China), restriction endonucleases, and T4 DNA ligase (Thermo Fisher Scientific, USA) were used for constructing plasmids. The methods of preparation and electrotransfer of *K. phaffii* competent cells were referred to *Pichia* Expression Kit (Invitrogen, USA). HiPure Yeast Plasmid Mini Kit (Magen, Guangzhou, China) was used for plasmid extraction from yeast.

### Construction of plasmids and yeast strains

The plasmids used in this study are listed in Table S1. The promoters and terminators used in the experiments were amplified directly from the *K. phaffii CBS7435* genome, and the empty plasmids were constructed based on the pUC19 plasmid (Thermo Fisher Scientific, USA) through Gibson Assembly. The *crtE* (GenBank: DQ016502.1), *crtI* (GenBank: AY177424.1), and *crtYB* (GenBank: AY177204.1) from *Phaffia rhodozyma* were synthesized and cloned downstream of the constitutive promoter *P*_*ADH2*_, *P*_*GAP*_, and *P*_*FBP1*_, yielding the plasmid pCrtI, pCrtE, and pCrtYB-1. The plasmid pCrtYB was generated by mutating *crtYB* to *crtYB*^*W61R*^ through a site-directed mutation of the plasmid pCrtYB-1. The gene *tHMG1* (GenBank: NM_001182434.1) was amplified from the *S. cerevisiae s288c* genome and cloned downstream of the constitutive promoter *P*_*FBA1*_, yielding the plasmid ptHMG1. A homologous analog of tRNA^Gly^ in *S. cerevisiae* has been demonstrated for gRNA processing (Zhang et al. 2019), and a promoter-tRNA-sgRNA Fusion Cassette enabled efficient knockdown of genes in *K. phaffii* (Dalvie et al. 2020). PARS1 (*P. pastoris*-specific autonomously replicating sequence, GenBank: M11199.1) for autonomous replication of gRNA plasmids in yeast cells (Nakamura et al. 2018), and tRNA^Gly^ was cloned from the *K. phaffii* genome for constructing empty guide RNA (gRNA) plasmids. Hammerhead ribozyme, HDV ribozyme, and gRNA scaffold were introduced into plasmids via iterative PCR amplification. A *PARS1*-based gRNA plasmid containing a *S. pyogenes* Cas9 (*SpCas9*, GenBank: UFQ04583.1) expression cassette (pGS188) was adapted from plasmid pPpT4_pHTX1-PARS1-HsCas9 (GenBank: MW604246.1) (Weninger et al. 2016). The plasmid pGS327 was derived from pGS188 by adding the *GFP* (GenBank: OM858837.1) expression cassette and *RFP* expression cassette (PDB: 7ARQ_AA), and replacing the Hammerhead ribozyme with the tRNA^Gly^ sequence. The *ERG10* (GenBank: NM_001183842.1), *ERG8* (GenBank: NM_001182727.1), *ERG12* (GenBank: NM_001182715.1), *mMVD1* (GenBank: NM_001183220.1), *ERG20* (GenBank: NM_001181600.1), *mERG13* (GenBank: NM_001182489.1), and *IDI1* (GenBank: NM_001183931.1) used in this study were cloned from the *S. cerevisiae s288c* genome and inserted downstream of different constitutive promoters, yielding plasmids pGS287 ~ pGS293. The guide RNA (gRNA) plasmids were constructed through restriction-ligation, and the sequences of gRNA are listed in Table S6. The gene *MEL1* (GenBank: LN997408.1) used to evaluate homologous recombination efficiency. The sequences of pGS188 (GenBank: OR298115), pGS327 (GenBank: OR298114), and pZW1 (GenBank: OR298116) are listed in Table S7. The sequences of used genes are listed in Table S4.

The methods of preparation and electrotransfer of *K. phaffii* competent cells were referred to *Pichia* Expression Kit (Invitrogen, USA). In all genomic integration and knockdown experiments, gRNA plasmids and DNA fragments were used in amounts of 2 µg, except for the upstream and downstream homology arms, which were used in amounts of 1 µg. All DNA fragments used in the manuscript for CRISPR/Cas9-mediated genomic integration ranged in length from 1800 to 3600 bp, except for the upstream and downstream homologous arms (~ 1000 bp). Donor DNA was obtained by concentrating the mixture of gRNA plasmids and DNA fragments (homologous arms and expression cassettes) and subsequently electrotransferred into yeast cells. The transformants were screened on YPD-G418 plates. The yeast strains used in this study are listed in Supplementary Table S2.

### Shake fermentation

A single colony was picked from a YPD plate and incubated in a test tube containing 5 mL YPD at 30 °C and 220 rpm overnight. The 500 μL cultures were transferred to 20 mL YPD and incubated for 12 h to prepare seed cultures. The seed cultures were transferred into 250-mL shake flasks containing 50 mL YPD at the initial OD_600_ of 0.2 and then fermented at 30 ℃ and 220 rpm for 7 days. Samples were taken every 24 h for product analysis during fermentation.

### Extraction and analysis of products

For the extraction of products, cells of 500 µL cultures were harvested through centrifugation, followed by washing twice with deionized water. The precipitate was resuspended with 500 µL 3 M HCl and was subsequently bathed in boiling water for 4 min and an ice water bath for 3 min for breaking yeast cells. Subsequently, the supernatant was removed by centrifugation and the precipitate was washed twice with deionized water and resuspended by adding 500 µL acetone to extract lycopene. The assay for lycopene was referenced to previously reported studies (Ma et al. 2019). Briefly, the HPLC was performed on an sb-aq liquid chromatography column (Agilent, USA) with two mobile phase solutions: mobile phase A was 90% acetonitrile in water (v:v), and mobile phase B was a mixture of methanol and isopropanol in the ratio (V:V) of 3:2. The HPLC was carried out using a gradient elution mode with a flow rate of 1 mL/min: 0–15 min, mobile phase A was from 100 to 10% and mobile phase B was from 0 to 90%; 15–30 min, mobile phase A was 10% and mobile phase B was 90%; 30–35 min, mobile phase A was from 10 to 100% and mobile phase B was from 90 to 0%.

## Results

### Repressing NHEJ to improve HR efficiency

**Fig. 1:**
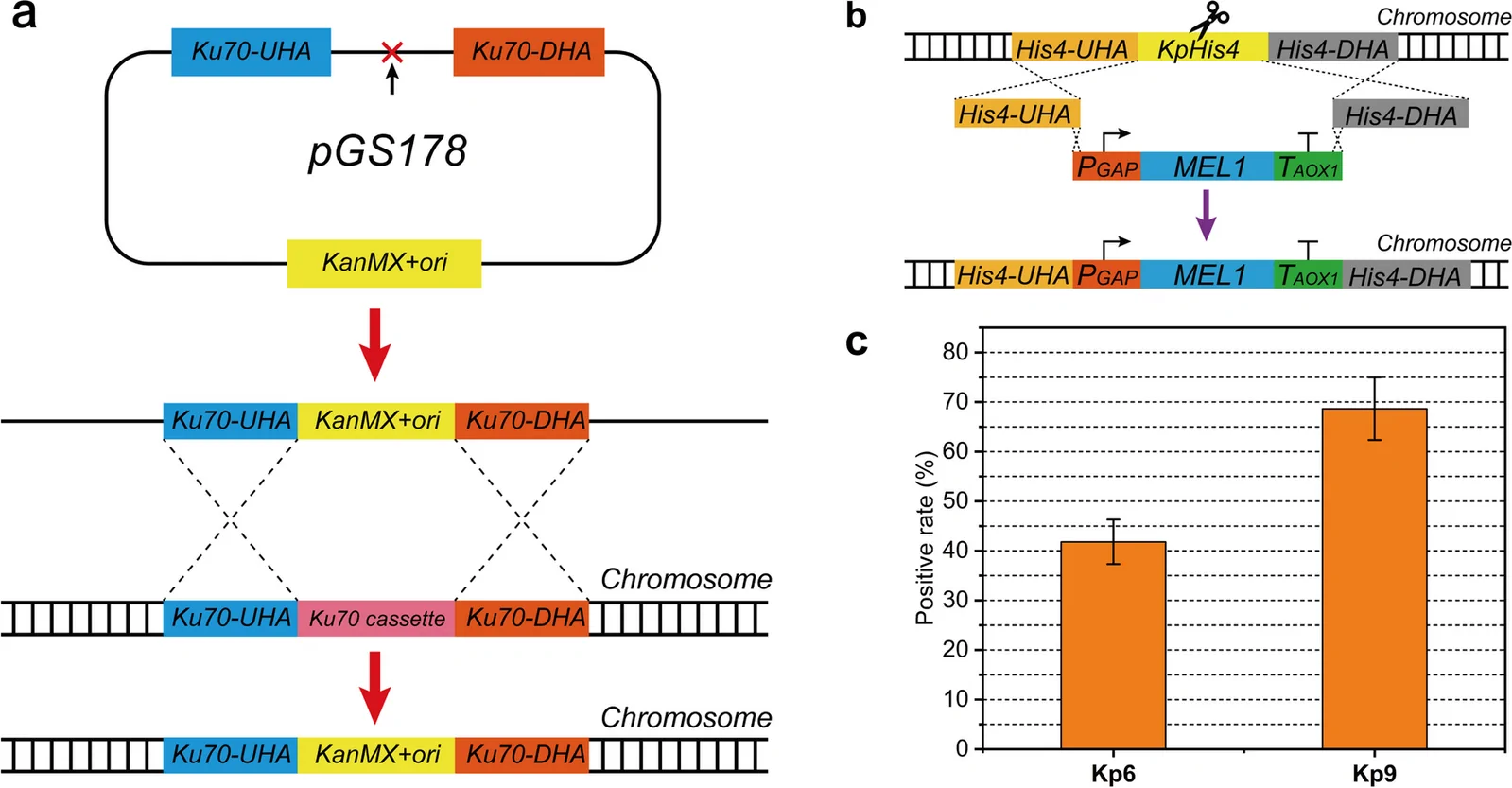
Improvement of HR efficiency in *K. phaffii*. **a** Design of *Ku70* knock-out. Knockout of *Ku70* was achieved by introducing the linearized plasmid pGS178 for single crossover. The red cross marked by a black arrow represents the restriction site used to linearize the plasmid. *Ku70-UHA* and *Ku70-DHA* were the upstream and downstream homologous arms of *Ku70*, respectively, and their lengths were both approximately 800 bp. **b** Integration of *MEL1* at *KpHis4* locus. The scissor means the cleavage site of CRISPR/Cas9.The length of flanking homologous sequences between the fragments ranged from 50 to 60 bp. **c** Integration efficiency of *MEL1* at *KpHis4* locus in *Kp6* and *Kp9*. The percentage of positive transformants was obtained via blue-white selection. All samples were run in triplicate

In contrast with *S. cerevisiae*, nonhomologous end-joining (NHEJ) dominates the double-strand break (DSB) repair process in *K. phaffii*. The *Ku70/Ku80* complex and *DNA ligase IV (DNL4)* play crucial roles in NHEJ-mediated DSB repair (Näätsaari et al. 2012). Accordingly, the *Ku70* gene was eliminated in *K. phaffii CBS7435* via double crossover with a linearized plasmid in this study (Fig. 1a). Additionally, *DNL4* was deleted through CRISPR/Cas9 based on *CBS7435ΔKu70* (Figure S1A, B).

To evaluate the efficiency of the CRISPR/Cas9 system in *CBS7435ΔKu70* (named *Kp6*) and *CBS7435ΔKu70ΔDNL4* (named *Kp9*), *KpHis4* was selected as the target locus for integration, and the *MEL1* gene encoding β-galactosidase was used as a reporter for integration efficiency (Fig. 1b). Yeast cells expressing *MEL1* can exhibit blue color on plates containing 5-bromo-4-chloro-3-indoxyl-α-D-galactopyranoside (X-α-gal), and the integration efficiency can be calculated via blue-white screening. The integration efficiency for *MEL1* was 35% and 65% in *Kp6* and *Kp9*, respectively, indicating that the efficiency of HR was superior in *K. phaffii* when both *Ku70* and *DNL4* were deficient (Fig. 1c). Additionally, a gRNA targeting *KanMX* was designed to substitute the *KanMX* expression cassette previously integrated at the *Ku70* locus with a *SpCas9* expression cassette in *Kp9* (the resulting strain was named *Kp12,* Figure S1C), enabling recycling of the G418 marker and making the gRNA plasmid almost half as small and more conducive for plasmid construction.

### Fast GFP-based marker recycling

**Fig. 2:**
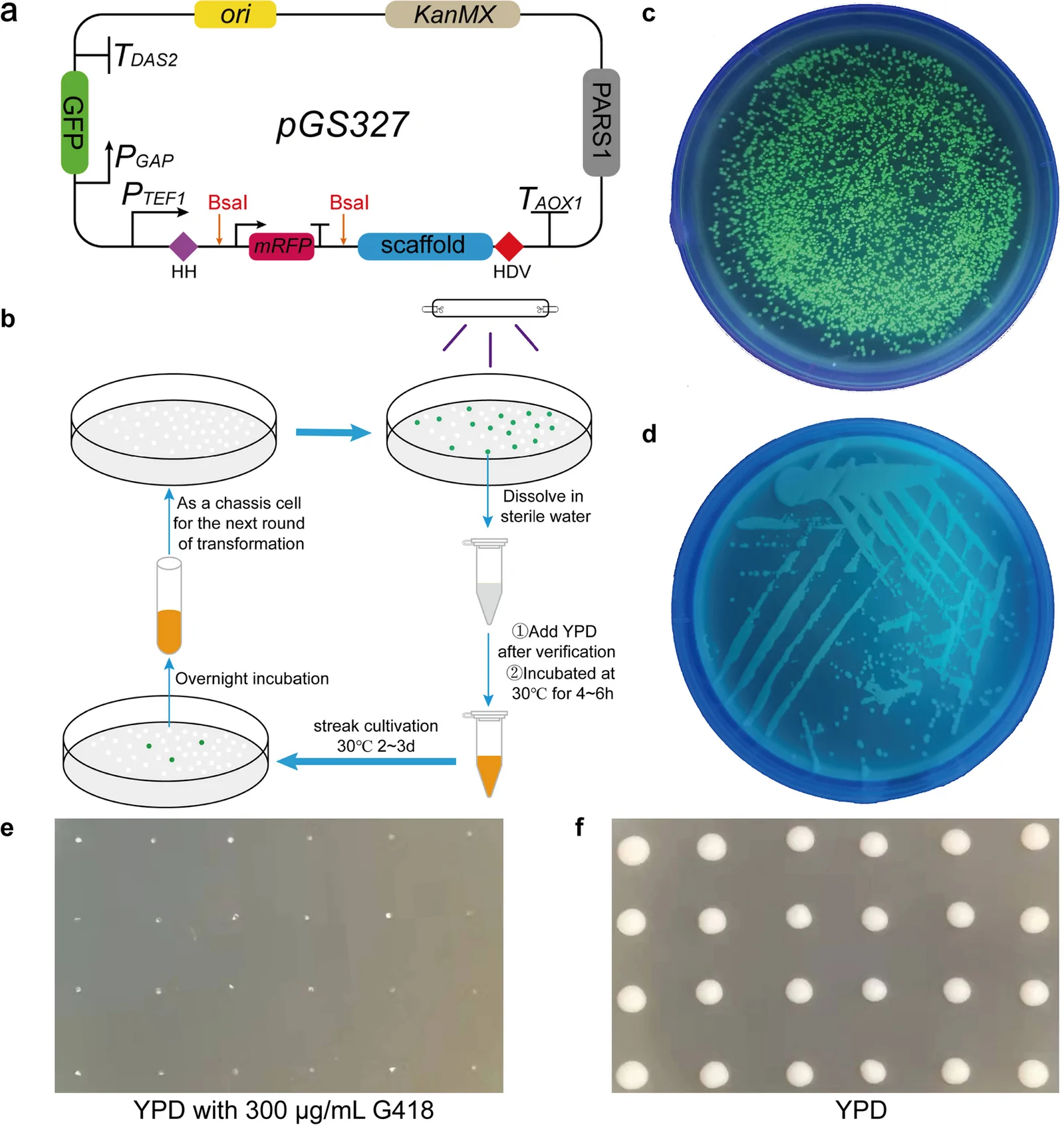
Design of a novel gRNA plasmid and its application in marker recycling. **a** Schematic description of the designed empty gRNA plasmid. GFP is the gene of green fluorescent protein, tRNA.^Gly^ is a self-processing tRNA element used for formation of precise final sgRNA sequences, mRFP is the gene of red fluorescent protein. BsaI is a restriction site. Scaffold is a sgRNA scaffold. HDV is hepatitis delta virus (HDV) ribozyme. *Ori* and *PARS1* were used for autonomous replication and amplification of the gRNA plasmid in *E. coli* and *K. phaffii*, respectively. *KanMX* shows kanamycin resistance and genomycin resistance in *E. coli* and *K. phaffii*, respectively. **b** An overview of the marker rapid recycling process. The gRNA plasmid with GFP makes the yeast cells exhibit green fluorescence, facilitating the screening of positive transformants. By incubating positive transformants under stress-free conditions, the rapid loss of the gRNA plasmid removes the green fluorescence in yeast, allowing for rapid recycling of labels. **c** A plate image of yeast carrying pGS327. **d** A picture of a streak plate. Yeast from **c** that showed fluorescence was incubated overnight in 5 mL YPD and then streaked on a YPD plate. Non-fluorescent clones from **d** were sequentially spot-blotted onto YPD-G418 (**e**) and YPD plates (**f**)

Constructing cell factories commonly requires iterative genetic engineering to rearrange metabolic networks, and gene editing usually requires appropriate markers for screening positive transformants. Restricted by the availability of markers, it is essential to recycle and reuse markers. To rapidly and conveniently confirm the elimination of episomal gRNA plasmids that contained selective markers, an appropriate reporter gene similar to *ScURA3* of *S. cerevisiae* was needed. However, experiments indicated that the growth of *CBS7435ΔURA3* was impaired to some extent (Lin Cereghino et al. 2001), suggesting that *KpURA3* (*URA3* of *K. phaffii*) is not an appropriate candidate for selective markers. The widely used *G418*, *Zeo*, and *KpHis4* screening markers in *K. phaffii* could not be visualized to determine whether the plasmids are lost. Furthermore, episomal plasmids in *K. phaffii* are easily lost without selective pressure. Therefore, an alternative method for recycling selective markers was considered. Green fluorescent protein (*GFP*) and red fluorescent protein (*RFP*), which could be observed in vivo, were attached to the gRNA plasmid. The novel gRNA plasmid (named *pGS327*) also carried *PARS1* (*P. pastoris*-specific autonomously replicating sequence) as well as the marker *KanMX* (Fig. 2a). On the one hand, the insertion of the 20 bp gRNA sequence during the plasmid construction phase causes the red fluorescent protein gene on the plasmid to be disrupted, and therefore displays white coloration after transformation into *E. coli*. Conversely, for an empty plasmid, the red fluorescent protein could be expressed normally in *E. coli*, and the strain would appear red. In this way, the success of plasmid construction can be visualized. Moreover, *GFP* was used to visualize yeast clones carrying the gRNA plasmid. Therefore, *GFP* could serve as an additional screening marker on selective plates to facilitate the screening of positive transformants during the transformation phase. During the marker recycling phase, *GFP* can serve as a reporter gene to visualize yeast cells not lost gRNA plasmids (Fig. 2b).

To verify the effectiveness of the marker recycling strategy, the plasmid *pGS327* was electrotransferred into *Kp12* and incubated on a YPD-G418 plate for 2–3 days. When the plate was exposed to a UV lamp (365 nm), most of the transformants exhibited green fluorescence (Fig. 2c). A single clone with green fluorescence was incubated overnight in 5 mL YPD and streaked onto a YPD plate for 2-day incubation. Examining the fluorescence intensity of YPD plates revealed that most of the clones grown were found to be non-green fluorescent (Fig. 2d), suggesting that these clones had already lost gRNA plasmids. To validate this assumption, the clones without green fluorescence were sequentially spot-blotted onto YPD-G418 and YPD plates. As presented in Fig. 2e, these clones grew normally on YPD plates, while their growth was severely inhibited on YPD-G418 plates, which demonstrated that clones without green fluorescence indeed lost gRNA plasmids. Therefore, the rapid and visualized recycling of selective markers was achieved in *K. phaffii* through a redesigned gRNA plasmid, in which *GFP* was used as an effective label for determining the loss of gRNA plasmids.

### Validation of precise multifragment assembly

**Fig. 3:**
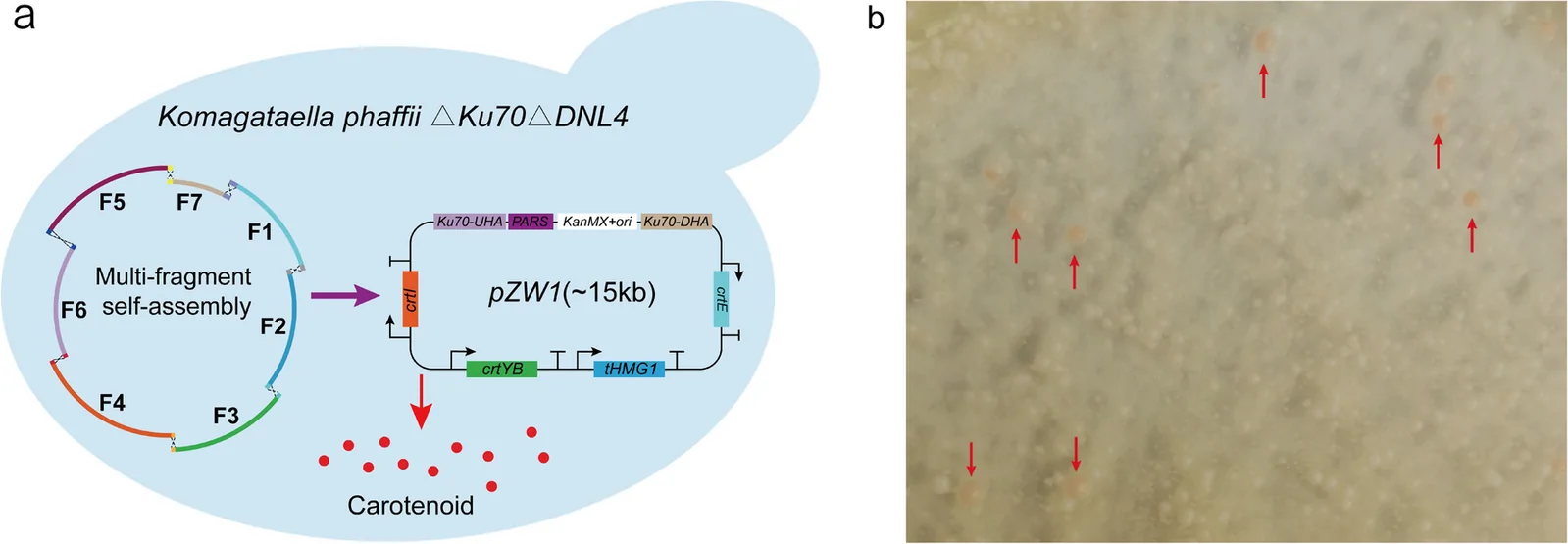
Multiple fragments in vivo were assembled to a complete plasmid. **a** Schematic map of multi-fragment assembly. Seven DNA fragments from different plasmids are intracellularly assembled into a complete plasmid, thus allowing yeast to synthesize carotenoids. The flanking homologous sequences between the fragments range from 50 to 60 bp. **b** Image of a plate with intracellular multi-fragment assembly. Orange-red colonies (pointed by red arrows) could indicate the presence of an intact plasmid in the yeast cell

Multifragment integration involves the correct assembly of multiple fragments into a single fragment in vivo. In this study, the lycopene synthesis pathway was introduced to evaluate the reliability of the precise assembly of multiple expression cassettes. Seven DNA fragments (named F1 to F7, the sequences of them are listed in Table S5) amplified from six plasmids were transformed into *Kp12*. Fragments F1, F2, F3, and F4 were expression cassettes for *crtI*, *crtYB*, *crtE*, and *tHMG1* from plasmids *pCrtI*, *pCrYB-1*, *pCrtE*, and *ptHMG1*, respectively. Fragment F5 containing *KanMX*, *ori*, and *PARS1* was derived from *pGS327*. Fragments F6 and F7 amplified from *pGS170* were upstream and downstream homology arms of the *KpHis4* locus. Meanwhile, homologous sequences of 55–60 bp were introduced at each end of the fragments. Theoretically, these fragments could assemble a complete episomal plasmid (named *pZW1*) de novo via HR (Fig. 3a), and yeast cells containing *pZW1* would synthesize carotenoids and exhibit an orange-red color. As displayed in Fig. 3b, 20.13% orange-red colonies grew on the YPD-G418 medium, suggesting that at least seven DNA fragments could be properly assembled into one fragment in *Kp12*. Subsequent sequencing also verified this result (Figures S2, S10, and S11).

### Single-locus multifragment integration

In yeast cells, the stable presence of episomal plasmids commonly relies on selective pressure. In the absence of selective pressure, episomal plasmids are more likely to be lost, resulting in cells containing decreasing amounts of plasmids, which could result in a dramatic decrease in yield. Additionally, there is an unequal distribution of plasmids during the cell division process. Although these problems could be alleviated by optimizing the culture conditions, this may not be very realistic for industrial applications. In contrast to episomal plasmids, genomic integration enables more stable expression of genes (Kong et al. 2022). Therefore, we followed up with research on the integration of multiple fragments into the genome in *K. phaffii*. To investigate the one-step multifragment integration efficiency, *KpHis4* and *KpADH900* were selected as the target loci.

**Fig. 4:**
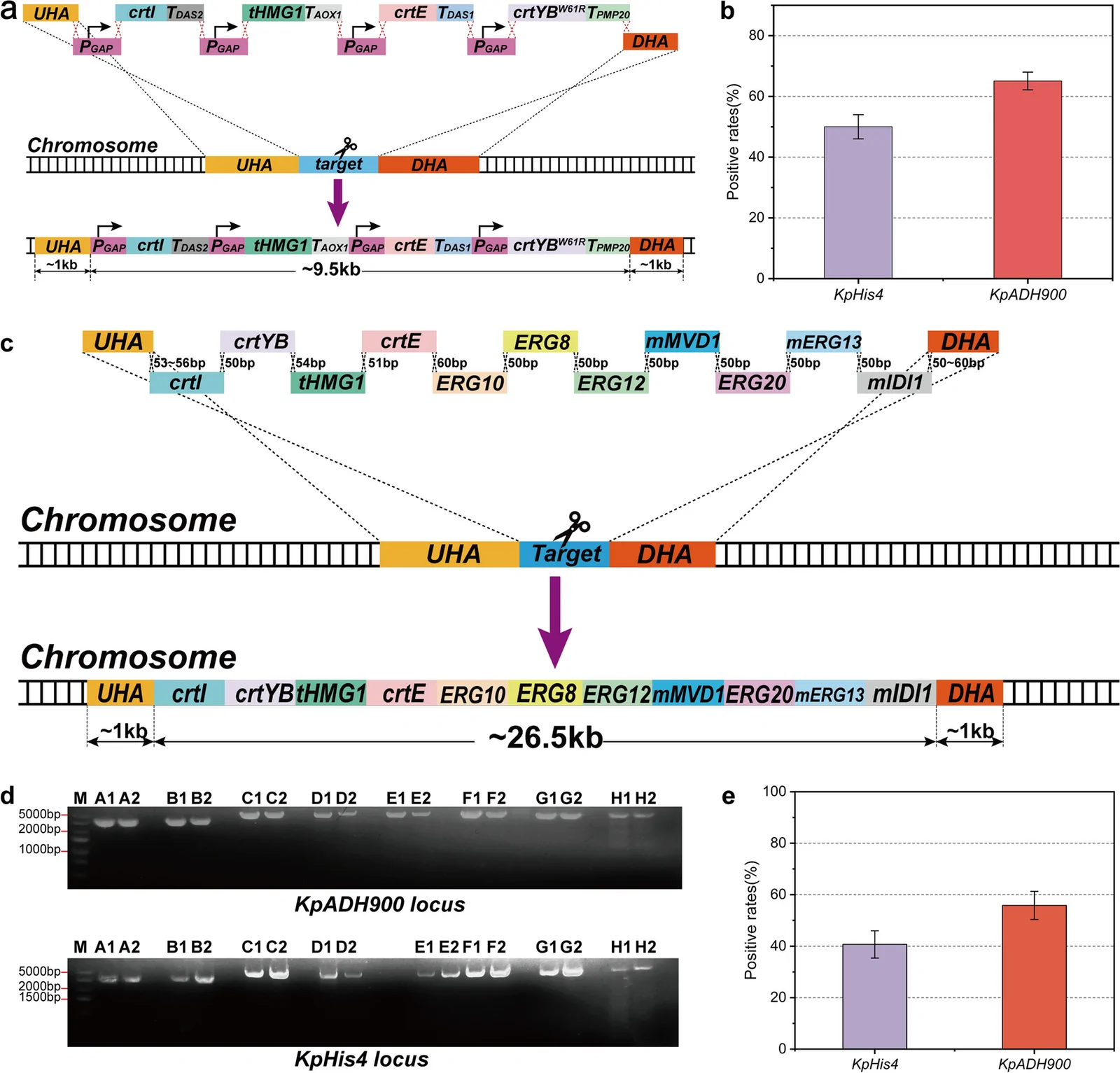
One-step multi-fragment integration at a single target. **a** Sketch of four expression cassettes integrated into a single locus in one step. The scissor in **a** and **c** means the cleavage site of CRISPR/Cas9. *UHA* and *DHA* are the upstream and downstream homologous arms of *ADH900* loci, respectively, and their lengths were both approximately 1000 bp. The length of flanking homologous sequences between the fragments ranged from 50 to 60 bp. **b** Integration efficiency of eight DNA fragments (four expression cassettes) integration at KpHis4 and KpADH900 locus. All samples were run in triplicate. **c** Schematic illustration of 11 expression cassettes integrated into a single locus in one step. **d** Verification of integrating 11 expression cassettes. Since the total length of the integrated fragments was excessively long, the entire long fragment was divided into several smaller segments A to H for verification. The empty lanes are used to separate the different fragments during the experiment to avoid confusion. **e** Integration efficiency of 11 expression cassettes integration at KpHis4 and KpADH900 locus. All samples were run in triplicate

The expression cassettes of *crtI*, *crtYB*^*W61R*^, *crtE*, and tHMG1 and the constitutive promoter *P*_*GAP*_ (*GAPDH* promoter) were chosen as DNA fragments for integration (Fig. 4a). After electrotransformation and incubation for 2–3 days, orange-red colonies grew on plates, indicating that the four genes were successfully integrated into these colonies. The integration efficiency at the *KpHis4* (named *zw106*) and *KpADH900* (named *zw107*) loci reached 50% and 63%, respectively (Fig. 4b). This suggested that efficient integration of eight DNA fragments could be realized in *K. phaffii*. It also demonstrated that the targets influence integration efficiency. To explore whether more fragments could be integrated in one step at a single locus, expression cassettes of the Mevalonate (MVA) pathway and lycopene synthesis pathway (Figure S8) were chosen to evaluate the integration efficiency (Fig. 4c). Each expression cassette carried a complete set of a promoter, gene, and terminator. These expression cassettes function in the lycopene synthesis pathway in *K. phaffii* starting from acetyl coenzyme A. Additionally, *KpHis4* and *KpADH900* were selected as targets. Homologous arms were all amplified from the *CBS7435* genome. The flanking homologous sequences between all fragments were 50–60 bp. After 2–3 days of incubation, orange-red clones were obtained on YPD-G418 plates and verified via colony PCR (Figs. 4d, S6, and S7). As shown in Fig. 4e, the integration efficiency of the *KpHis4* (named *zw109*) and *KpADH900* (named *zw69*) loci reached 40% and 57%, respectively.

### Large DNA fragment deletion

The appropriate simplification of genomes could lower the complexity of microbial systems, contributing to a better understanding of cellular circuitry and providing a solid basis for the optimization of chassis microbes (Leprince et al. 2012). Large DNA fragment deletion, an effective means of genome streamlining, was therefore carried out in this research. First, a specific gRNA targeting *CrtE* was designed in an attempt to eliminate the lycopene synthesis pathway in *zw69*. The 1000 bp upstream and downstream homologous arms, amplified from the *CBS7435* genome and transformed into *zw69* together with the gRNA plasmid, were used to repair the DSB (Figure S3). If the large fragment could be successfully knocked out, colonies unable to synthesize lycopene would appear white. Unfortunately, no white clones were obtained on plates, suggesting that a single gRNA might not be sufficient for large fragment deletion.

**Fig. 5:**
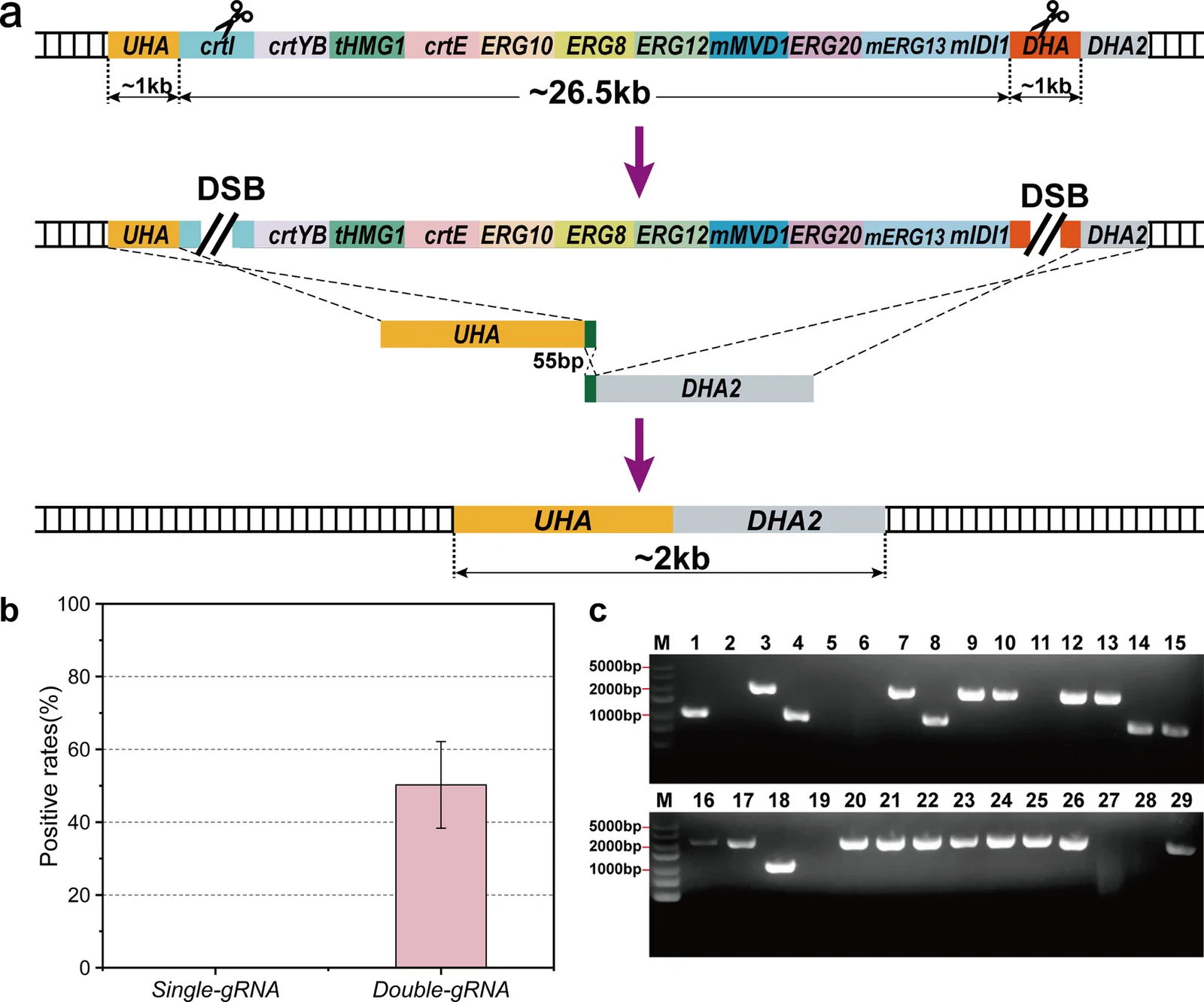
Large DNA fragment knockout mediated by CRISPR/Cas9. **a** Schematic description of double gRNA plasmid-mediated large DNA fragment knockout. The homologous arms were both around 1000 bp. The scissors mean the cleavage sites of CRISPR/Cas9. *UHA* and *DHA* are the upstream and downstream homologous arms of *ADH900* loci, respectively. DHA2 is a nucleic acid sequence about 1000 bp immediately downstream of DHA and acts as the downstream homologous arm for the large DNA fragment knockout. **b** The efficiency of large DNA fragment knockout mediated by single gRNA plasmid and double gRNA plasmids. All samples were run in triplicate. **c** Agarose gel electrophoresis images of large fragment knockout. The clone that was correctly knocked out a large DNA fragment should show a 2000-bp band. The blank channels may be due to unsuccessful knockout of the target fragment, which is too large to be amplified. The fragments around 1000 bp in the gel map may be mis-repaired due to the loss of large DNA fragments

We presumed that the failed deletion might be attributed to inefficient substitution of the fragment through one DSB and that two or more DSBs may be required for deleting large DNA fragments, as in *S. cerevisiae* (Li et al. 2018). Thus, gRNA2 targeting *crtI* and gRNA3 targeting the *KpADH900* downstream homologous arm were synthesized to form two remote DSBs located at opposite ends of the fragment to be knocked out (Fig. 5a). As expected, white clones grew on plates after transformation. The lycopene synthesis pathway, of which the length was 27 kb, was verified to have been precisely eliminated in these clones by colony PCR and Sanger sequencing, and the efficiency reached 50% (Fig. 5b, c, Figure S9).

To further test the feasibility of the CRISPR/Cas9 system for large fragment knockout in *K. phaffii*, three neutral gRNA targeting sites, II-4, II-5, and II-6, were chosen from *Kp12* chromosome II (Cai et al. 2021), where II-4 and II-5 were separated by 39 kb and II-4 and II-6 were separated by 247 kb (Figure S4A). The gRNA plasmids of II-4 and II-5 and the gRNA plasmids of II-4 and II-6 were separately introduced into *Kp12* together with the relevant homologous arms. However, no transformants were obtained on the plates (Figure S4B, C).

### Yeast combinatorial libraries for multi-gene pathways

**Fig. 6:**
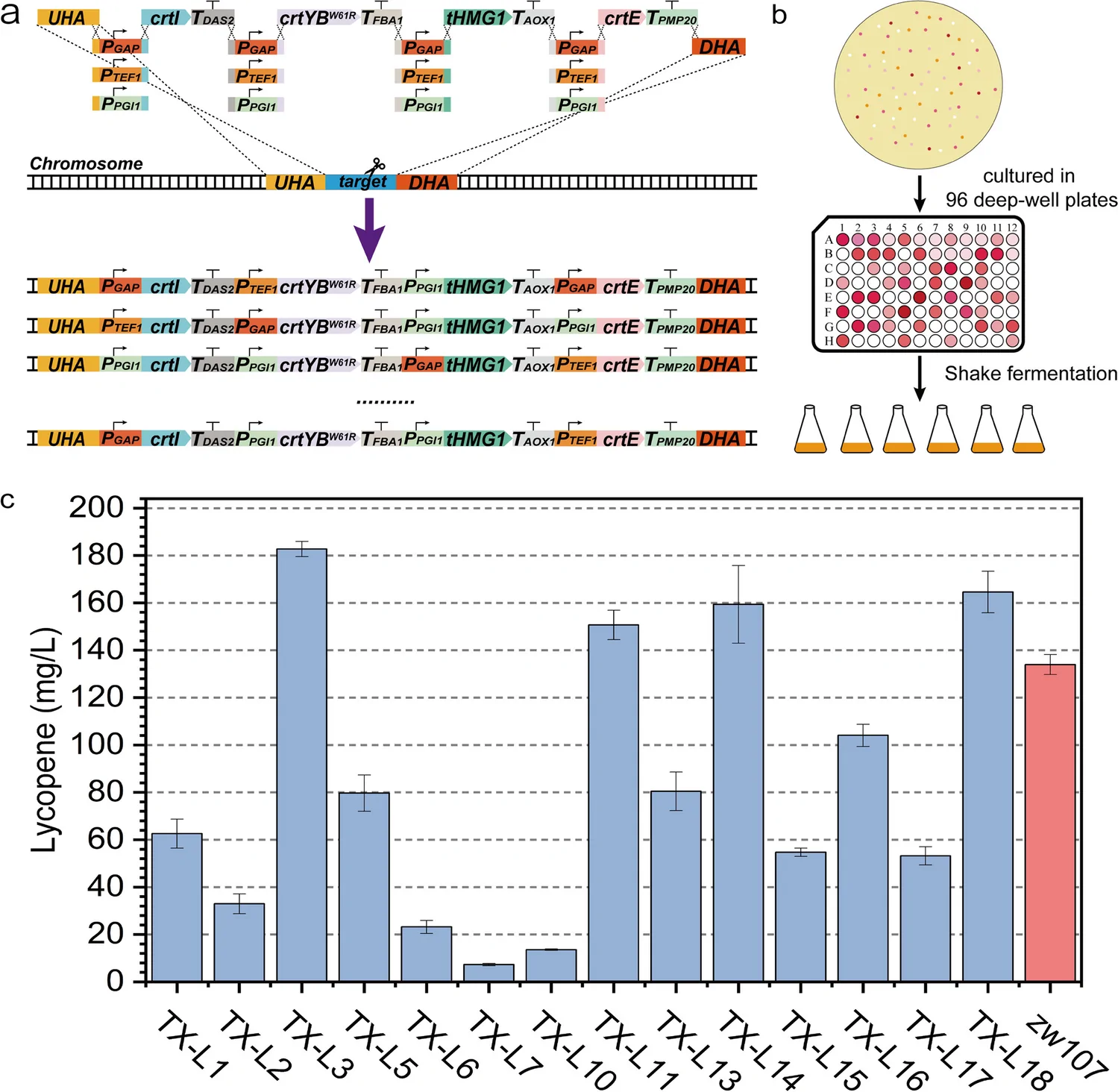
Design and validation of a yeast combinatorial library. **a** Schematic illustration of a yeast combinatorial library. The library consists of three promoters, four genes, and four terminators. The scissor means the cleavage site of CRISPR/Cas9. *UHA* and *DHA* are the upstream and downstream homologous arms of *ADH900* loci, respectively, and their lengths were both approximately 1000 bp. **b** The screening process for yeast libraries. **c** Fermentation results of multiple clones picked from yeast libraries. Results of fermentation show that the maximum yield was 25 times higher than the minimum yield. All samples were run in triplicate

The rearrangement of metabolic networks usually requires the coordination of multiple genes. Rapidly achieving a better coordination of multi-gene expression intensity is one of the most important aspects of metabolic engineering. Therefore, a strategy for constructing yeast combinatorial libraries of multigene metabolic pathways was proposed (EauClaire et al. 2016). In this study, *crtE*, *crtYB*^*W61R*^, *crtI*, and *tHMG1* were used to construct a yeast library of the lycopene synthesis pathway in *K. phaffii*, with the expectation that a better combination could be obtained for lycopene synthesis by optimizing the expression ratios between these genes. As shown in Fig. 6a, three promoters, which were in decreasing order of strength as *P*_*GAP*_ > *P*_*TEF1*_ > *P*_*PGI1*_, were furnished for *crtE*, *crtYB*^*W61R*^, *crtI*, and *tHMG1*. Therefore, a total of 18 DNA fragments were used as elements for the overlap assembly, and the lengths of the overlapping sequences between adjacent fragments were all 55–60 bp. The integration process was mediated by gRNA targeting the *KpADH900* locus and accompanied by spontaneous free-assembly between different promoters and the same gene, and final assembly into a complete pathway to construct a yeast combinatorial library to obtain an optimal solution for metabolic pathway assembly within a certain context. Theoretically, the combinatorial assembly of the three promoters with four genes would yield 81 different combinations, which could be easily screened for transformants with different levels of lycopene production according to the colors of clones (Figure S5).

Next, 288 clones were transferred into 96 deep-well plates and cultured for 2–3 days (Fig. 6b). Then, 14 clones with different color grades were picked for sequencing and fermentation, with *zw107* as a control. As expected, the results of both sequencing and HPLC analysis demonstrated that different combinations were generated in this yeast library and exhibited various phenotypes, and the lycopene yields varied from 7.28 to 182.73 mg/L (Fig. 6c). The maximum production was 25 times higher than the minimum. The sequencing results showed that strain TX-L3, in which *crtI*, *crtYB*^*W61R*^, *tHMG1*, and *crtE* were controlled by *P*_*GAP*_,* P*_*TEF1*_,* P*_*PGI1*_, and *P*_*TEF1*_, respectively, achieved maximum yield. The strain TX-L7, in which *crtI*, *crtYB*^*W61R*^, *tHMG1*, and *crtE* were controlled by *P*_*PGI1*_,* P*_*GAP*_,* P*_*PGI1*_, and *P*_*GAP*_, respectively, achieved minimum production. Moreover, we found that in higher-yielding strains, *crtE* (encoding GGPP synthases) and *crtI* (encoding phytoene desaturase), which catalyze the synthesis of GGPP from FPP and lycopene from phytoene, respectively, were under the control of the stronger promoter *P*_*GAP*_ or *P*_*TEF1*_, suggesting that GGPP synthases and phytoene desaturase are two of the key enzymes in lycopene synthesis, which is consistent with previous reports (Zhang et al. 2020). Thus, it is practical and rapid to coordinate the expression levels of individual genes in the metabolic pathway to achieve a better combination.

## Discussion

The methylotrophic yeast, *K. phaffii*, has been increasingly engineered into cell factories for the synthesis of various secondary metabolites in recent years (Cai et al. 2022; Guo et al. 2021; Zuo et al. 2022). To enable the construction of cell factories, several CRISPR-based gene editing systems have been established in *K. phaffii* (Cai et al. 2021; Liu et al. 2019; Nishi et al. 2022; Weninger et al. 2015, 2016; Zhang et al. 2021); however, compared to *S. cerevisiae*, *K. phaffii* still has potential for improvement in marker recycling, multifragment integration, etc. In this research, *Kp6* (*CBS7435ΔKu70*) and *Kp9* (*CBS7435ΔKu70ΔDNL4*) were constructed, and the *KpHis4* locus was selected as the target to evaluate the homologous recombination efficiency of the two strains. The result showed better homologous recombination efficiency in *Kp9*, which is consistent with previously reported research results (Ito et al. 2018; Nishi et al. 2022). In addition, our previous experiments showed that the growth of the *K. phaffii* ΔURA3 strain was impaired, which is consistent with previously reported research results (Lin Cereghino et al. 2001); therefore, rapid marker recycling could not be performed similarly to that in *S. cerevisiae* on screening plates supplemented with 5-fluoroorotic acid (Kotaka et al. 2009; Moon et al. 2022). To simplify the marker recycling process, we redesigned the gRNA plasmid to determine whether plasmid elimination had been achieved through the visualization of green fluorescent protein. Compared to plasmid elimination methods in other systems (Liao et al. 2021; Liu et al. 2019; Weninger et al. 2018; Yang et al. 2020), the method we designed takes less time and may be more conducive to iterative gene editing. In addition, the *GFP* in the plasmid could assist in screening positive transformants.

When constructing cell factories, the number of genes integrated in one step is one of the factors that affect the efficiency of the construction. Although Nishi et al. (2022) realized the integration of four heterologous gene expression cassettes totaling 7.8 kb, which were divided into 10 fragments, in a single transformation in *K. phaffii*, there is still a gap compared to *S. cerevisiae* in which up to 15 fragments can be integrated in a one-step transformation at a single locus (Jakociunas et al. 2015). In this study, the one-step integration of 11 expression cassettes for a complete lycopene synthesis pathway with a total length of 26.5 kb at a single locus was achieved in *K. phaffii* for the first time. To the best of our knowledge, both the number of fragments and the total length of the fragments are by far the highest integrated in *K. phaffii* to date. Previously, NHEJ-based knockout of large fragments was performed by introducing DSBs via CRISPR/Cas9 in *K. phaffii*, and a positive rate of ~ 40% was achieved for a 4000-bp DNA fragment, but only ~ 2% of transformants were cleanly eliminated (Schusterbauer et al. 2022). Additionally, although Schusterbauer et al. (2022) achieved a “nonclean knockout” of a 100-kb DNA fragment in *K. phaffii*, the transformants lost the complete 3′ end of a chromosome rather than showing precise knockout of a large fragment inside a chromosome, as in our work. In addition to CRISPR/Cas9-based large fragment knockout, Zhang et al. (2021) realized CRISPR/Cpf1-based large fragment knockout with 11% efficiency for 20 kb. In this study, we first precisely knocked out a 27-kb DNA fragment inside a chromosome by introducing two DSBs at both ends of the fragment to be knocked out, thereby completely eliminating the lycopene synthesis pathway with 50% efficiency, which represented superior efficiency and length to those of the knockout achieved in the CRISPR/Cpf1 system (Zhang et al. 2021). Subsequently, three neutral targets, II-4, II-5, and II-6, were selected for testing larger DNA fragment knockouts in this study. However, in several repetitions of the experiment, no transformants successfully grew. The results of sequence alignment of proteins in the NCBI database indicated that the genes encoding the ribosomal biosynthesis protein *RLP24* and the large ribosomal subunit biogenesis protein *JIP5* were included in the region between II-4 and II-5, both of which are homologous analogs that are essential in *S. cerevisiae* for biogenesis of the large ribosomal subunit. Therefore, we speculated that the lack of transformants might be due to gene loss preventing the growth of cells. Further studies may be needed to determine the deletion of larger fragments. These results show that our tool could be used to efficiently and rapidly edit genomes, accelerating metabolic network rearrangements and facilitating the construction of cell factories.

We first constructed yeast combinatorial libraries in *K. phaffii* for the fast balancing of metabolic pathways, which has been demonstrated previously in *S. cerevisiae* (EauClaire et al. 2016). Through the yeast combinatorial library, we successfully obtained different lycopene synthesis strains with yields up to 182.73 mg/L in shake flask fermentation. This result suggests that in constructing metabolic pathways, not all genes need to be under the control of a strong promoter. Instead, the metabolic pathway should be optimized by combining the demands of the metabolic flux and the catalytic efficiency (specific activity) of the enzyme itself. In previous reports, the ratio of multiple enzymes associated with product synthesis was determined by individually overexpressing each protein and measuring the resulting catalytic capacity in vitro, which in turn informed the optimization of the metabolic pathway in vivo (Liu et al. 2017). This is an effective strategy to improve product synthesis. However, in many cases, the catalytic capacity of each individual enzyme is not easily determined. For example, some enzymes may be difficult to heterologously express or purify, or their substrates/products may not be easily detected, which makes the implementation of this strategy difficult. Other researchers optimized the copy number of each gene to determine the rate-limiting step and enhance the yield of the product (Lv et al. 2019), which involves an extensive and time-consuming workload when the metabolic pathway involves a large number of genes. In contrast, yeast combinatorial libraries could effectively facilitate the regulation and assembly of metabolic pathways. Additionally, this strategy could be extended to the free assembly of other elements, such as different genes, terminators, and transcriptional regulators, making the construction of cell factories more efficient and more time-saving.

In conclusion, this work further expands the application of CRISPR/Cas9 in *K. phaffii*; provides newly developed rapid, efficient, and continuous genome editing tools; and establishes a flexible and concise genome editing method. In this research, marker recycling, multifragment integration, and large fragment knockout were optimized, and yeast combinatorial libraries were introduced for the first time. In summary, the CRISPR/Cas9 system described in this study provides a more efficient tool for the construction of *K. phaffii* cell factories.

## Supplementary Information

Below is the link to the electronic supplementary material.

Supplementary file1 (PDF 14283 KB)

## Data Availability

All data generated or analyzed during this study are included in this published article (and its supplementary information files).
