# Supplementary material for: CRISPR/Cas9-based toolkit for rapid marker recycling and combinatorial libraries in Komagataella phaffii
Source: Appl Microbiol Biotechnol. 2024 Feb 7;108(1):197. doi: 10.1007/s00253-024-13037-1 (PMC10850205; doi:10.1007/s00253-024-13037-1)
Supplement: Supplementary file 1 — Supplementary file1 (PDF 14283 KB) [file 253_2024_13037_MOESM1_ESM.pdf]

APPLIED MICROBIOLOGY AND BIOTECHNOLOGY

CRISPR/Cas9-based toolkit for rapid marker recycling and combinatorial libraries in *Komagataella phaffii*

Wei Zhou[a], Yuanyi Li[a], Guosong Liu[a], Weichuang Qin[a], Dongzhi Wei[a], Fengqing Wang[a]*, Bei Gao[a]*

[a] State Key Laboratory of Bioreactor Engineering, New world Institute of Biotechnology, East China University of Science and Technology, Shanghai, 200237, China

*Corresponding authors. Address: East China University of Science and Technology, P.O.B.311, 130 Meilong Road, Shanghai 200237, China. Fax: +8621 64250068

Email address: gaobei@ecust.edu.cn (B. Gao); fqwang@ecust.edu.cn (F. Wang)

Telephone: 18019078882 (B. Gao)

Table S1. Plasmids list

Name	Description	Source	GenBank
pGS124	P_{GAP}-*MEL1*-T_{AOX1}	This study	OR763072
pGS170	*KpHis4* upstream and downstream homologous arms	This study	OR763072
pGS178	used for single-crossover to knock out *Ku70*	This study	OR763070
pGS188	empty gRNA plasmid with an *SpCas9* expression cassette	This study	OR298115
pGS188-KpDNL4	gRNA plasmid targeting DNA ligase IV with an *SpCas9* expression cassette	This study	OR763068
pGS188-KanMX	gRNA plasmid targeting KanMX with an *SpCas9* expression cassette	This study	OR763069
pGS188-KpHis4	gRNA plasmid targeting KpHis4 with an *SpCas9* expression cassette	This study	OR763067
pGS327	empty gRNA plasmid,no *SpCas9* expression cassette,derived from pGS188	This study	OR298114
pCrtE	P_{ADH2}-*crtE*-T_{DAS1}	This study	OR763076
pCrtI	P_{GAP}-*crtI*-T_{DAS2}	This study	OR763075
PCrtYB-1	P_{FBP1}-*crtYB*-T_{FBA1}	This study	OR763073
pCrtYB	P_{FBP1}-*crtYB*^{W61R}-T_{FBA1}	This study	OR763074
ptHMG1	P_{FBA1}-*tHMG1*-T_{AOX1}	This study	OR763077
pGS287	P_{GAP}-*ERG10*-T_{PMP20}	This study	OR763066
pGS288	P_{PGI1}-*ERG8*-T_{TPI1}	This study	OR763065
pGS289	P_{PGD}-*ERG12*-T_{CAT1}	This study	OR763064
pGS290	P_{TAL1}-*MVD1*-T_{FDH1}	This study	OR763063
pGS291	P_{MSR1c3}-*ERG20*-T_{ADH2}	This study	OR763062

pGS292	P_{GPM1}-*mERG13*-T_{FLD1}	This study	OR763061
pGS293	P_{TPI1}-*mIDI1*-T_{Gcw14}	This study	OR763060
pGS332	gRNA expression cassette for targeting *KpHis4* derived from pGS327	This study	OR763059
pGS492	gRNA expression cassette for targeting *KpADH900* derived from pGS327	This study	OR763058
pGS575	gRNA expression cassette for targeting *KpADH900-2* derived from pGS327	This study	OR763055
pGS566	gRNA expression cassette for targeting *II-4* derived from pGS327	This study	OR763057
pGS567	gRNA expression cassette for targeting *II-5* derived from pGS327	This study	OR763056
pGS568	gRNA expression cassette for targeting *II-6* derived from pGS327	This study	OR763054

Table S2. Strains list

**Strain**	**Description**	**Source**
CBS7435	*Komagataella phaffii* Wild type	Purchased from Invitrogen
Kp6	*CBS7435 Ku70△*::*KanMX*	This study
Kp9	*CBS7435 DNL4△Ku70△*::*KanMX*	This study
Kp12	*CBS7435 DNL4△Ku70△*::P_{HXT1}-*SpCas9*-T_{DAS1}	This study
zw69	*CBS7435 DNL4△Ku70△*::P_{HXT1}-*SpCas9*-T_{DAS1} *ADH900*:: P_{GAP}-*crtI*-T_{DAS2}-P_{FBP1}-*crtYB*-T_{FBA1}-P_{FBA1}-*tHMG1*-T_{AOX1}-P_{ADH2}-*crtE*-T_{DAS1}-P_{GAP}-*ERG10*-T_{PMP20}-P_{PGI1}-*ERG8*-T_{TPI1}-P_{PGD1}-*ERG12*-T_{CAT1}-P_{TAL1}-*mMVD1*-T_{FDH1}-P_{MSR1c3}-*ERG20*-T_{ADH2}-P_{GPM1}-*mERG13*-T_{FLD1}-P_{TPI1}-*mIDI1*-T_{Gcw14}	This study
Zw106	*CBS7435 DNL4△Ku70△*::P_{HXT1}-*SpCas9*-T_{DAS1} *ADH900*:: P_{GAP}-*crtI*-T_{DAS2}-P_{GAP}-*tHMG1*-T_{AOX1}-P_{GAP}-*crtE*-T_{DAS1}-P_{GAP}-$crtYB^{W61R}$-T_{PMP20}	This study
zw107	*CBS7435 DNL4△Ku70△*::P_{HXT1}-*SpCas9*-T_{DAS1} *ADH900*:: P_{GAP}-*crtI*-T_{DAS2}-P_{GAP}-*tHMG1*-T_{AOX1}-P_{GAP}-*crtE*-T_{DAS1}-P_{GAP}-$crtYB^{W61R}$-T_{PMP20}	This study
Zw109	*CBS7435 DNL4△Ku70△*::P_{HXT1}-*SpCas9*-T_{DAS1} *ADH900*:: P_{GAP}-*crtI*-T_{DAS2}-P_{FBP1}-*crtYB*-T_{FBA1}-P_{FBA1}-*tHMG1*-T_{AOX1}-P_{ADH2}-*crtE*-T_{DAS1}-P_{GAP}-*ERG10*-T_{PMP20}-P_{PGI1}-*ERG8*-T_{TPI1}-P_{PGD1}-*ERG12*-T_{CAT1}-P_{TAL1}-*mMVD1*-T_{FDH1}-P_{MSR1c3}-*ERG20*-T_{ADH2}-P_{GPM1}-*mERG13*-T_{FLD1}-P_{TPI1}-*mIDI1*-T_{Gcw14}	This study

Table S3. Primer list

**Name**	**sequence**	**Application**
KpDNL4-DHA-F	AGTGGATCAATGCGAAGGATAGGGCTGGGTGATGATCTACCAATC	used for knock out *KpDNL4*
KpDNL4-DHA-R	GACGAGGACTTCGCTAACCGTCACG	
KpDNL4-UHA-F	ACGCGCGCGTGGTAGAAGTTTATCT	
KpDNL4-UHA-R	ATCCTTCGCATTGATCCACTGTTGGAGCGCATTCTATTCTCATAC	
kpHis4-UHA-F	GCAAAGTTGGTAGATGTGACTTCCACT	

kpHis4-UHA-R-pGAP	GGACGAGGACACCAAGACATTTCTACAAAA ACTGGGAAAGGAGCTTCTAACACAGG	used for integration of *MEL1* at KpHis4 locus
PGAP-F-KpHis4-UHA	CCTGTGTTAGAAGCTCCTTTCCCAGTTTTTG TAGAAATGTCTTGGTGTCCTCGTCC	
TAOX1-R-KpHis4-DHA	GACTACACCAGGGCAAGTCTCAACTTTTCA CTTAATCTTCTGTACTCTGA	
KpHis4-DHA-F-TAOX1	TCAGAGTACAGAAGATTAAGTGAAAAGTTGA GACTTGCCCTGGTGTAGTC	
KpHis4-DHA-R1	TTAAATAAGTCCCAGTTTCTCCATACGAACC	
kpKu70-DHA-F	AATACATCCAGTTCAAGTTACCTAAACAAAT CAAAGCCTCCAAAAAGGCAGATCTCATCG	used for integration of *SpCas9* at *KpKu70* locus
KpKu70-DHA-R	CGTTAATCTACAGTTAGCAGAGTGC	
KpKu70-UHA-F	CCAGATGTAATCACCGCCGCTGAAT	
kpKu70-UHA-R	ACTCCCAAAGTGTCAGTCACAAGGGTCTCC TCACTGACATTCTCTAAGATGATCTGGAGC	
pHTX1-F-Ku70-DHA	CGATGAGATCTGCCTTTTTGGAGGCTTTGAT TTGTTTAGGTAACTTGAACTGGATGTATT	
TDAS1-R-KpKu70	GATCATCTTAGAGAATGTCAGTGAGGAGAC CCTTGTGACTGACACTTTGGGAGTCCCTAT	
KpADH900-DHA-F-TPMP20	AGACACCAGGTCATTGAACTAACGACAAGG TTAAGACGGTGGCTACTACCATC	used for multi-fragment integration
KpADH900-DHA-R	ATTATTGACTATACACTTAATCGGCAGATAA GAAAATCTTG	
KpADH900-UHA-F	TCAAATGTAACATTGGAATGATTCGATTTGA	
KpADH900-UHA-R-pGAP	TGGACGAGGACACCAAGACATTTCTACAAA AAGGGTGAGGAACGTTCTCAACCATA	
pGAP-F-ADH900-UHA	CAATATGGTTGAGAACGTTCCTCACCCTTTT TGTAGAAATGTCTTGGTGTCCTCGT	
pGAP-F-TAOX1	CTCTTCAGAGTACAGAAGATTAAGTGAAATT TTTGTAGAAATGTCTTGGTGTCCTCGTCC	
pGAP-F-TDAS1	GGACTCCCAAAGTGTCAGTCACAAGGGTCT TTTTGTAGAAATGTCTTGGTGTCCTCGTCC	
pGAP-F-TDAS2	AAAGGAGGAACCAGTTTACGAACCCCGTCT TTTTGTAGAAATGTCTTGGTGTCCTCGTCC	
TAOX1-R-pGAP	GACGAGGACACCAAGACATTTCTACAAAAAT TTCACTTAATCTTCTGTACTCTGAAGAG	
TDAS1-R-pGAP	GGACGAGGACACCAAGACATTTCTACAAAA AGACCCTTGTGACTGACACTTTGGGAGTCC	
TDAS2-R-pGAP	GGACGAGGACACCAAGACATTTCTACAAAA AGACGGGGTTCGTAAACTGGTTCCTCCTTT	
TPMP20-R-KpADH900-DHA	GCAGATGGTAGTAGCCACCGTCTTAACCTT GTCGTTAGTTCAATGACCTGGTG	
pGAP-F-TPMP20	TCCAGACACCAGGTCATTGAACTAACGACA AGTTTTTGTAGAAATGTCTTGGTGTCCTCG	
TPMP20-R-pGAP	CGAGGACACCAAGACATTTCTACAAAAACTT GTCGTTAGTTCAATGACCTGGTGTCTGGA	
KpHis4-DHA-F-TGcw14	GACTAGTATATACGCATCCTTCCCGTCGTTA GTTGAGACTTGCCCTGGTGTAGTC	
pADH2-F-TAOX1	TCCTCTTCAGAGTACAGAAGATTAAGTGAAA CGCAGCGTTTTCTGACGGTACTAGA	
pFBA1-F-TFBA1	GCTCACAGCGTACACATCACCACTCATTTCT CTATTGAACGGcttgaaatttgg	
pFBP1-F-TDAS2	AGGAACCAGTTTACGAACCCCGTCTGCTCA AACGAGTGGAGAGGGAAATC	
pGPM1-F-TADH2	ATCTAGCGACTCTGGCAGATAAGATATCAAT ACCTTGGGTTATTAGTAGTGTCCG	
pMSR1c3-F-TFDH1	gtttctccttcaaactTCTCACCTCCTTTTAGTGCAG TTGCGCCGTGATTTC	

pPGD1-F-TTPI1	CTGTATCTGTAACCCAAGGGGTGGATTATGGTAGaatcatcaattggaatGACCCTATCG	
pPGI1-F-TPMP20	ACCAGGTCATTGAACTAACGACAAGTAGGTCACCTGTGTTACTAGATGTC	
pTAL1-F-TCAT1	TCGAaacatcatcagaaaaCTAAACCAGAGATCGTGTTTTGATTAAGATTGCTGCTAC	
pTPI1-F-TFLD1	TCGCGGTATTGATCGACGCTCTgtgaaaTCAACGAGACACTCTTCCGTCAGT	
TADH2-R-pGPM1	ACTACTAATAACCCAAGGTATTGATATCTTATCTGCCAGAGTCGCTAGATctgga	
TAOX1-R-pADH2	TACCGTCAGAAAACGCTGCGTTTCACTTAATCTTCTGTACTCTGAAGAGGAGTGG	
TCAT1-R-pTAL1	AATCTTAATCAAAACACGATCTCTGGTTTAGttttctgatgatgttTCGATCATCG	
TDAS2-R-pFBP1	GATTTCCCTCTCCACTCGTTTGAGCAGACGGGGTTCGTAAACTGGTTCCT	
TFBA1-R-pFBA1	ccaaatttcaagCCGTTCAATAGAGAAATGAGTGGTGATGTGTACGCTGTGAGC	
TFDH1-R-pMSR1c3	AATCACGGCGCAACTGCACTAAAAGGAGGTGAGAagtttgaaggagaaactC	
TFLD1-R-pTPI1	TGACGGAAGAGTGTCTCGTTGAtttcacAGAGCGTCGATCAATACCGCGAAG	
TGcw14-R-KpHis4-DHA	GACTACACCAGGGCAAGTCTCAACTAACGACGGGAAGGATGCGTATATAC	
TPMP20-R-pPGI1	GACATCTAGTAACACAGGTGACCTACTTGTCGTTAGTTCAATGACCTGGT	
TTPI1-R-pPGD1	attccaattgatgattCTACCATAATCCACCCCTTGGGTTACAGATACAG	
KpADH900-DHA-F-TGcw14	GACTAGTATATACGCATCCTTCCCGTCGTTGTTAAGACGGTGGCTACTACCATCT	
TGcw14-R-KpADH900-DHA	TCCGCAGATGGTAGTAGCCACCGTCTTAACAACGACGGGAAGGATGCGTATATAC	
KpADH900-DHA-2-F-KpADH900-UHA	ATATGGTTGAGAACGTTCCTCACCCGGATCCTGGATTTACAACTGCAAGACCTATGGA	Large DNA fragment knockout
KpADH900-DHA-2-R	ATAGCCATGGTTGAGTCCAATCTATGTG	
KpADH900-UHA-R-KpADH900-DAH2	ATCCATAGGTCTTGCAGTTGTAAATCCAGGATCCGGGTGAGGAACGTTCTCAACCATA	
CrtE-F	ATGGATTACGCGAACATCCTCACAGC	Construction of yeast libraries
CrtI-F	ATGGGAAAAGAACAAGATCAGG	
CrtYB-F	ATGACGGCTCTCGCATATTACC	
DAS2TT-R	GACGGGGTTCGTAAACTGGTTCCTC	
FBA1TT-R	ATGAGTGGTGATGTGTACGCTGTGAGC	
KpADH900-UHA-R	GGGTGAGGAACGTTCTCAACCATA	
pGAP-F-CrtE	GCTGTGAGGATGTTCGCGTAATCCATCTGTGTTTTGATAGttgttcaattgattg	
pGAP-F-TAOX1-2	tttgatgtttttcttgGTATTTCCCACTCCTCTTCAGAGTACAGAAGATTAAGTGAAA	
pGAP-F-TDAS2-2	TTTTTTGACCAATAAATGGAACAGGAAGGAAAGGAGGAACCAGTTTACGAACCCCGTC	
pGAP-F-TFBA1-2	ATAACACTTTGTTGTGAGACAGTAATAAAAAGCTCACAGCGTACACATCACCACTCAT	
pGAP-F2-KpADH900-UHA	GACTCTTGCGCAGGCCGTCGTACGATAAGGGCAATATGGTTGAGAACGTTCCTCACCC	
pGAP-R-CrtI	CCTGATCTTGTTCTTTTCCCATcTGTGTTTTGATAGttgttcaattgattg	
pGAP-R-CrtE-2	TATCATCCTGAGGAGTAAACTCGAGTGGAATTGCTGTGAGGATGTTCGCGTAATCCAT	

pGAP-R-CrtI-2	CGATACCACATCCCACGATGATAGCTGTGG GTTTATCCTGATCTTGTTCTTTTCCCAT	
pGAP-R-CrtYB	GGTAATATGCGAGAGCCGTCATcTGTGTTTT GATAGttgttcaattgattg	
pGAP-R-CrtYB-2	GACCAAGAATTGGGAGAGTATAGATCAGAT GGATCTGGTAATATGCGAGAGCCGTCAT	
pGAP-R-tHMG1	GACTTCAGTTTTCACCAATTGGTCCATcTGT GTTTTGATAGttgttcaattgattg	
pGAP-R-tHMG1-2	TTTGTACAGGAGCAGTAAAAGACTTCTTGGT GACTTCAGTTTTCACCAATTGGTCCAT	
TAOX1-R	TTTCACTTAATCTTCTGTACTCTGAAGAGGA GTGG	
tHMG1-F	ATGGACCAATTGGTGAAAACTGAAGTC	
pTEF1-F-KpADH900-UHA	TATGGTTGAGAACGTTCCTCACCCATAACTG TCGCCTCTTTTATCTGC	
pTEF1-F-TAOX1	CTCTTCAGAGTACAGAAGATTAAGTGAAAAT AACTGTCGCCTCTTTTATCTGC	
pTEF1-F-TDAS2	GAGGAACCAGTTTACGAACCCCGTCATAAC TGTCGCCTCTTTTATCTGC	
pTEF1-F-TFBA1	GCTCACAGCGTACACATCACCACTCATATAA CTGTCGCCTCTTTTATCTGC	
pTEF1-R-CrtI	CCTGATCTTGTTCTTTTCCCATcGTTGGCGA ATAACTAAAATGTATGTAGTGAg	
pTEF1-R-CrtE	GCTGTGAGGATGTTCGCGTAATCCATCGTT GGCGAATAACTAAAATGTATGTAGTGAg	
pTEF1-R-CrtYB	GGTAATATGCGAGAGCCGTCATcGTTGGCG AATAACTAAAATGTATGTAGTGAg	
pTEF1-R-tHMG1	GACTTCAGTTTTCACCAATTGGTCCATcGTT GGCGAATAACTAAAATGTATGTAGTGAg	
pPGI1-F-KpADH900-UHA	TATGGTTGAGAACGTTCCTCACCCTAGGTC ACCTGTGTTACTAGATGTCGGATG	
pPGI1-F-TAOX1	CCTCTTCAGAGTACAGAAGATTAAGTGAAAT AGGTCACCTGTGTTACTAGATGTCGGATG	
pPGI1-F-TDAS2	GAGGAACCAGTTTACGAACCCCGTCTAGGT CACCTGTGTTACTAGATGTCGGATG	
pPGI1-F-TFBA1	GCTCACAGCGTACACATCACCACTCATTAG GTCACCTGTGTTACTAGATGTCGGATG	
pPGI1-R-CrtI	CCTGATCTTGTTCTTTTCCCATcTTTATCAAT TGGTTGGAGTTGAATTATTCCGGAAG	
pPGI1-R-CrtE	GCTGTGAGGATGTTCGCGTAATCCATCTTTA TCAATTGGTTGGAGTTGAATTATTCCGGAAG	
pPGI1-R-CrtYB	GGTAATATGCGAGAGCCGTCATcTTTATCAA TTGGTTGGAGTTGAATTATTCCGGAAG	
pPGI1-R-tHMG1	GACTTCAGTTTTCACCAATTGGTCCATcTTTA TCAATTGGTTGGAGTTGAATTATTCCGGAAG	

Table S4. Gene list and sequence

SpCas9 DNA sequence.
ATGGACAAGAAGTACTCCATTGGGCTCGATATCGGCACAAACAGCGTCGGCTGGGC CGTCATTACGGACGAGTACAAGGTGCCGAGCAAAAAATTCAAAGTTCTGGGCAATAC CGATCGCCACAGCATAAAGAAGAACCTCATTGGCGCCCTCCTGTTCGACTCCGGGG AGACGGCCGAAGCCACGCGGCTCAAAAGAACAGCACGGCGCAGATATACCCGCAG AAAGAATCGGATCTGCTACCTGCAGGAGATCTTTAGTAATGAGATGGCTAAGGTGGAT GACTCTTTCTTCCATAGGCTGGAGGAGTCCTTTTTGGTGGAGGAGGATAAAAAGCAC GAGCGCCACCCAATCTTTGGCAATATCGTGGACGAGGTGGCGTACCATGAAAAGTAC CCAACCATATATCATCTGAGGAAGAAGCTTGTAGACAGTACTGATAAGGCTGACTTGC GGTTGATCTATCTCGCGCTGGCGCATATGATCAAATTTCGGGGACACTTCCTCATCGA

GGGGGACCTGAACCCAGACAACAGCGATGTCGACAAACTCTTTATCCAACTGGTTCA
GACTTACAATCAGCTTTTCGAAGAGAACCCGATCAACGCATCCGGAGTTGACGCCAA
AGCAATCCTGAGCGCTAGGCTGTCCAAATCCCGGCGGCTCGAAAACCTCATCGCAC
AGCTCCCTGGGGAGAAGAAGAACGGCCTGTTTGGTAATCTTATCGCCCTGTCACTCG
GGCTGACCCCCAACTTTAAATCTAACTTCGACCTGGCCGAAGATGCCAAGCTTCAAC
TGAGCAAAGACACCTACGATGATGATCTCGACAATCTGCTGGCCCAGATCGGCGACC
AGTACGCAGACCTTTTTTTGGCGGCAAAGAACCTGTCAGACGCCATTCTGCTGAGTG
ATATTCTGCGAGTGAACACGGAGATCACCAAAGCTCCGCTGAGCGCTAGTATGATCA
AGCGCTATGATGAGCACCACCAAGACTTGACTTTGCTGAAGGCCCTTGTCAGACAG
CAACTGCCTGAGAAGTACAAGGAAATTTTCTTCGATCAGTCTAAAAATGGCTACGCCG
GATACATTGACGGCGGAGCAAGCCAGGAGGAATTTTACAAATTTATTAAGCCCATCTT
GGAAAAAATGGACGGCACCGAGGAGCTGCTGGTAAAGCTTAACAGAGAAGATCTGT
TGCGCAAACAGCGCACTTTCGACAATGGAAGCATCCCCCACCAGATTCACCTGGGC
GAACTGCACGCTATCCTCAGGCGGCAAGAGGATTTCTACCCCTTTTTGAAAGATAAC
AGGGAAAAGATTGAGAAAATCCTCACATTTCGGATACCCTACTATGTAGGCCCCCTCG
CCCGGGGAAATTCCAGATTCGCGTGGATGACTCGCAAATCAGAAGAAACCATCACTC
CCTGGAACTTCGAGGAAGTCGTGGATAAGGGGGCCTCTGCCCAGTCCTTCATCGAA
AGGATGACTAACTTTGATAAAAATCTGCCTAACGAAAAGGTGCTTCCTAAACACTCTC
TGCTGTACGAGTACTTCACAGTTTATAACGAGCTCACCAAGGTCAAATACGTCACAGA
AGGGATGAGAAAGCCAGCATTCCTGTCTGGAGAGCAGAAGAAAGCTATCGTGGACC
TCCTCTTCAAGACGAACCGGAAAGTTACCGTGAAACAGCTCAAAGAAGACTATTTCA
AAAAGATTGAATGTTTCGACTCTGTTGAAATCAGCGGAGTGGAGGATCGCTTCAACG
CATCCCTGGGAACGTATCACGATCTCCTGAAAATCATTAAAGACAAGGACTTCCTGGA
CAATGAGGAGAACGAGGACATTCTTGAGGACATTGTCCTCACCCTTACGTTGTTTGA
AGATAGGGAGATGATTGAAGAACGCTTGAAAACTTACGCTCATCTCTTCGACGACAA
AGTCATGAAACAGCTCAAGAGGCGCCGATATACAGGATGGGGGCGGCTGTCAAGAA
AACTGATCAATGGGATCCGAGACAAGCAGAGTGGAAAGACAATCCTGGATTTTCTTA
AGTCCGATGGATTTGCCAACCGGAACTTCATGCAGTTGATCCATGATGACTCTCTCA
CCTTTAAGGAGGACATCCAGAAAGCACAAGTTTCTGGCCAGGGGGACAGTCTTCAC
GAGCACATCGCTAATCTTGCAGGTAGCCCAGCTATCAAAAAGGGAATACTGCAGACC
GTTAAGGTCGTGGATGAACTCGTCAAAGTAATGGGAAGGCATAAGCCCGAGAATATC
GTTATCGAGATGGCCCGAGAGAACCAAACTACCCAGAAGGGACAGAAGAACAGTAG
GGAAAGGATGAAGAGGATTGAAGAGGGTATAAAAGAACTGGGGTCCCAAATCCTTAA
GGAACACCCAGTTGAAAACACCCAGCTTCAGAATGAGAAGCTCTACCTGTACTACCT
GCAGAACGGCAGGGACATGTACGTGGATCAGGAACTGGACATCAATCGGCTCTCCG
ACTACGACGTGGATCATATCGTGCCCCAGTCTTTTCTCAAAGATGATTCTATTGATAAT
AAAGTGTTGACAAGATCCGATAAAAATAGAGGGAAGAGTGATAACGTCCCCTCAGAA
GAAGTTGTCAAGAAAATGAAAAATTATTGGCGGCAGCTGCTGAACGCCAAACTGATC
ACACAACGGAAGTTCGATAATCTGACTAAGGCTGAACGAGGTGGCCTGTCTGAGTTG
GATAAAGCCGGCTTCATCAAAAGGCAGCTTGTTGAGACACGCCAGATCACCAAGCAC
GTGGCCCAAATTCTCGATTCACGCATGAACACCAAGTACGATGAAAATGACAAACTGA
TTCGAGAGGTGAAAGTTATTACTCTGAAGTCTAAGCTGGTTTCAGATTTCAGAAAGGA
CTTTCAGTTTTATAAGGTGAGAGAGATCAACAATTACCACCATGCGCATGATGCCTAC
CTGAATGCAGTGGTAGGCACTGCACTTATCAAAAAATATCCCAAGCTTGAATCTGAAT
TTGTTTACGGAGACTATAAAGTGTACGATGTTAGGAAAATGATCGCAAAGTCTGAGCA
GGAAATAGGCAAGGCCACCGCTAAGTACTTCTTTTACAGCAATATTATGAATTTTTTCA
AGACCGAGATTACACTGGCCAATGGAGAGATTCGGAAGCGACCACTTATCGAAACAA
ACGGAGAAACAGGAGAAATCGTGTGGGACAAGGGTAGGGATTTCGCGACAGTCCG
GAAGGTCCTGTCCATGCCGCAGGTGAACATCGTTAAAAAGACCGAAGTACAGACCG
GAGGCTTCTCCAAGGAAAGTATCCTCCCGAAAAGGAACAGCGACAAGCTGATCGCA
CGCAAAAAAGATTGGGACCCCAAGAAATACGGCGGATTCGATTCTCCTACAGTCGCT
TACAGTGTACTGGTTGTGGCCAAAGTGGAGAAAGGGAAGTCTAAAAAACTCAAAAGC
GTCAAGGAACTGCTGGGCATCACAATCATGGAGCGATCAAGCTTCGAAAAAAACCCC
ATCGACTTTCTCGAGGCGAAAGGATATAAAGAGGTCAAAAAAGACCTCATCATTAAGC
TTCCCAAGTACTCTCTCTTTGAGCTTGAAAACGGCCGGAAACGAATGCTCGCTAGTG
CGGGCGAGCTGCAGAAAGGTAACGAGCTGGCACTGCCCTCTAAATACGTTAATTTCT
TGTATCTGGCCAGCCACTATGAAAAGCTCAAAGGGTCCCCCGAAGATAATGAGCAGA

AGCAGCTGTTCGTGGAACAACACAAACACTACCTTGATGAGATCATCGAGCAAATAA
GCGAATTCTCCAAAAGAGTGATCCTCGCCGACGCTAACCTCGATAAGGTGCTTTCTG
CTTACAATAAGCACAGGGATAAGCCCATCAGGGAGCAGGCAGAAAACATTATCCACT
TGTTTACTCTGACCAACTTGGGCGCGCCTGCAGCCTTCAAGTACTTCGACACCACCA
TAGACAGAAAGCGGTACACCTCTACAAAGGAGGTCCTGGACGCCACACTGATTCATC
AGTCAATTACGGGGCTCTATGAAACAAGAATCGACCTCTCTCAGCTCGGTGGAGATC
CAAAGAAGAAAAGAAAAGTTTAA

The blue bases encode the *SpCas9* protein sequence, the red bases encode the nuclear localization signal peptide *SV40*, and the black encodes the stop codon

GFP DNA sequence.

ATGAGTAAAGGAGAAGAACTTTTCACTGGAGTTGTCCCAATTCTTGTTGAATTAGATG
GTGATGTTAATGGGCACAAATTTTCTGTCAGTGGAGAGGGTGAAGGTGATGCTACAT
ACGGAAAGCTTACCCTTAAATTTATTTGCACTACTGGAAAACTACCAGTTCCATGGCC
AACACTTGTCACTACTTTCTCTTATGGTGTTCAATGCTTTTCCCGTTATCCGGATCATA
TGAAACGGCATGACTTTTTCAAGAGTGCCATGCCCGAAGGTTATGTACAGGAACGCA
CTATATCTTTCAAAGATGACGGGAACTACAAGACGCGTGCTGAAGTCAAGTTTGAAG
GTGATACCCTTGTTAATCGTATCGAGTTAAAAGGTATTGATTTTAAAGAAGATGGAAAC
ATTCTCGGACACAAACTCGAGTACAACTATAACTCACACAATGTATACATCACGGCAG
ACAAACAAAAGAATGGAATCAAAGCTAACTTCAAAATTCGCCACAACATTGAAGATGG
CTCAGTTCAACTAGCAGACCATTATCAACAAAATACTCCAATTGGCGATGGCCCTGTC
CTTTTACCAGACAACCATTACCTGTCGACACAATCTGCCCTTTCGAAAGATCCCAACG
AAAAGCGTGACCACATGGTCCTTCTTGAGTTTGTAACTGCTGCTGGGATTACACATG
GCATGGATGAACTATACAAATAG

CrtI DNA sequence.

ATGGGAAAAGAACAAGATCAGGATAAACCCACAGCTATCATCGTGGGATGTGGTATC
GGTGGAATCGCCACTGCCGCTCGTCTTGCTAAAGAAGGTTTCCAGGTCACGGTGTT
CGAGAAGAACGACTACTCCGGAGGTCGATGCTCTTTAATCGAGCGAGATGGTTATCG
ATTCGATCAGGGGCCCAGTTTGCTGCTCTTGCCAGATCTCTCCAAGCAGACATTCGA
AGATTTGGGAGAGAAGATGGAAGATTGGGTCGATCTCATCAAGTGTGAACCCAACTA
TGTTTGCCACTTCCACGATGAAGAGACTTTCACTCTTTCAACCGACATGGCGTTGCT
CAAGCGGGAAGTCGAGCGTTTTGAAGGCAAAGATGGATTTGATCGGTTCTTGTCGTT
TATCCAAGAAGCCCACAGACATTACGAGCTTGCTGTCGTTCACGTCCTGCAGAAGAA
CTTCCCTGGCTTCGCAGCATTCTTACGGCTACAGTTCATTGGCCAAATCCTGGCTCTT
CACCCCTTCGAGTCTATCTGGACAAGAGTTTGTCGATATTTCAAGACCGACAGATTAC
GAAGAGTCTTCTCGTTTGCAGTGATGTACATGGGTCAAAGCCCATACAGTGCGCCCG
GAACATATTCCTTGCTCCAATACACCGAATTGACCGAGGGCATCTGGTATCCGAGAG
GAGGCTTTTGGCAGGTTCCTAATACTCTTCTTCAGATCATCAAGCGCAACAATCCCTC
AGCCAAGTTCAATTTCAACGCTCCAGTTTCCCAGGTTCTTCTCTCTCCTGCCAAGGA
CCGAGCGACTGGTGTTCGACTTGAATCCGGCGAGGAACATCACGCCGATGTTGTGA
TTGTCAATGCTGACCTCGTTTACGCCTCCGAGCACTTGATTCCTGACGATGCCAGAA
ACAAGATTGGCCAACTGGGTGAAGTCAAGAGAAGTTGGTGGGCTGACTTAGTTGGT
GGAAAGAAGCTCAAGGGAAGTTGCAGTAGTTTGAGCTTCTACTGGAGCATGGACCG
AATCGTGGACGGTCTGGGCGGACACAATATCTTCTTGGCCGAGGACTTCAAGGGAT
CATTCGACACAATCTTCGAGGAGTTGGGTCTCCCAGCCGATCCTTCCTTTTACGTGA
ACGTTCCCTCGCGAATCGATCCTTCTGCCGCTCCCGAAGGCAAAGATGCTATCGTCA
TTCTTGTGCCGTGTGGCCATATCGACGCTTCGAACCCTCAAGATTACAACAAGCTTG
TTGCTCGGGCAATGAAGTTTGTGATCCACACGCTTTCCGCCAAGCTTGGACTTCCCG
ACTTTGAAAAAATGATTGTGGCAGAGAAGGTTCACGATGCTCCCTCTTGGGAGAAAG
AATTCAACCTCAAGGACGGAAGCATCTTGGGACTGGCTCACAACTTTATGCAAGTTC
TTGGTTTCAGGCCGAGCACCAGACATCCCAAGTATGACAAGTTGTTCTTTGTCGGGG
CTTCGACTCATCCCGGAACTGGGGTTCCCATCGTCTTGGCTGGAGCCAAGTTAACT
GCCAACCAAGTTCTCGAATCCTTTGACCGATCCCCAGCTCCAGATCCCAATATGTCA
CTCTCCGTACCATATGGAAAACCTCTCAAATCAAATGGAACGGGTATCGATTCTCAGG
TCCAGCTGAAGTTCATGGATTTGGAGAGATGGGTATACCTTTTGGTATTGTTGATTGG
GGCCGTGATCGCTCGATCCGTTGGTGTTCTTGCTTTCTGA

CrtYB DNA sequence.

ATGACGGCTCTCGCATATTACCAGATCCATCTGATCTATACTCTCCCAATTCTTGGTCT
TCTCGGTCTGCTCACTTCCCCGATTTTGACAAAATTTGACATCTACAAAATATCGATCC
TCGTATTTATTGCGTTTAGTGCAACCACACCATGGGACTCATGGATCATCAGAAATGG
CGCATGGACATATCCATCAGCGGAGAGTGGCCAAGGCGTGTTTGGAACGTTTCTAGA
TGTTCCATATGAAGAGTACGCTTTCTTTGTCATTCAAACCGTAATCACCGGCTTGGTC
TACGTCTTGGCAACTAGGCACCTTCTCCCATCTCTCGCGCTTCCCAAGACTAGATCG
TCCGCCCTTTCTCTCGCGCTCAAGGCGCTCATCCCTCTGCCCATTATCTACCTATTTA
CCGCTCACCCCAGCCCATCGCCCGACCCGCTCGTGACAGATCACTACTTCTACATG
CGGGCACTCTCCTTACTCATCACCCCACCTACCATGCTCTTGGCAGCATTATCAGGC
GAATATGCTTTCGATTGGAAAAGTGGCCGAGCAAAGTCAACTATTGCAGCAATCATGA
TCCCGACGGTGTATCTGATTTGGGTAGATTATGTTGCTGTCGGTCAAGACTCTTGGTC
GATCAACGATGAGAAGATTGTAGGGTGGAGGCTTGGAGGTGTACTACCCATTGAGGA
AGCTATGTTCTTCTTACTGACGAATCTAATGATTGTTCTGGGTCTGTCTGCCTGCGAT
CATACTCAGGCCCTATACCTGCTACACGGTCGAACTATTTATGGCAACAAAAAGATGC
CATCTTCATTTCCCCTCATTACACCGCCTGTGCTCTCCCTGTTTTTTAGCAGCCGACC
ATACTCTTCTCAGCCAAAACGTGACTTGGAACTGGCAGTCAAGTTGTTGGAGGAAAA
GAGCCGGAGCTTTTTTGTTGCCTCGGCTGGATTTCCTAGCGAAGTTAGGGAGAGGC
TGGTTGGACTATACGCATTCTGCCGGGTGACTGATGATCTTATCGACTCTCCTGAAGT
ATCTTCCAACCCGCATGCCACAATTGACATGGTCTCCGATTTTCTTACCCTACTATTTG
GGCCCCCGCTACACCCTTCGCAACCTGACAAGATCCTTTCTTCGCCTTTACTTCCTC
CTTCGCACCCTTCCCGACCCACGGGAATGTATCCCCTCCCGCCTCCTCCTTCGCTCT
CGCCTGCCGAGCTCGTTCAATTCCTTACCGAAAGGGTTCCCGTTCAATACCATTTCG
CCTTCAGGTTGCTCGCTAAGTTGCAAGGGCTGATCCCTCGATACCCACTCGACGAAC
TCCTTAGAGGATACACCACTGATCTTATCTTTCCTTTATCGACAGAGGCAGTCCAGGC
TCGGAAGACGCCTATCGAGACCACAGCTGACTTGCTGGACTATGGTCTATGTGTAGC
AGGCTCAGTCGCCGAGCTATTGGTCTATGTCTCTTGGGCAAGTGCACCAAGTCAGGT
CCCTGCCACCATAGAAGAAAGAGAAGCTGTGTTAGTGGCAAGCCGAGAGATGGGAA
CTGCCCTTCAGTTGGTGAACATTGCTAGGGACATTAAAGGGGACGCAACAGAAGGG
AGATTTTACCTACCACTCTCATTCTTTGGTCTTCGGGATGAATCAAAGCTTGCGATCC
CGACTGATTGGACGGAACCTCGGCCTCAAGATTTCGACAAACTCCTCAGTCTATCTC
CTTCGTCCACATTACCATCTTCAAACGCCTCAGAAAGCTTCCGGTTCGAATGGAAGA
CGTACTCGCTTCCATTAGTCGCCTACGCAGAGGATCTTGCCAAACATTCTTATAAGGG
AATTGACCGACTTCCTACCGAGGTTCAAGCGGGAATGCGAGCGGCTTGCGCGAGCT
ACCTACTGATCGGCCGAGAGATCAAAGTCGTTTGGAAAGGAGACGTCGGAGAGAGA
AGGACAGTTGCCGGATGGAGGAGAGTACGGAAAGTCTTGAGTGTGGTCATGAGCG
GATGGGAAGGGCAGTAA

CrtYB^{W61R} DNA sequence.

ATGACGGCTCTCGCATATTACCAGATCCATCTGATCTATACTCTCCCAATTCTTGGTCT
TCTCGGTCTGCTCACTTCCCCGATTTTGACAAAATTTGACATCTACAAAATATCGATCC
TCGTATTTATTGCGTTTAGTGCAACCACACCATGGGACTCATGGATCATCAGAAATGG
CGCAAGAACATATCCATCAGCGGAGAGTGGCCAAGGCGTGTTTGGAACGTTTCTAGA
TGTTCCATATGAAGAGTACGCTTTCTTTGTCATTCAAACCGTAATCACCGGCTTGGTC
TACGTCTTGGCAACTAGGCACCTTCTCCCATCTCTCGCGCTTCCCAAGACTAGATCG
TCCGCCCTTTCTCTCGCGCTCAAGGCGCTCATCCCTCTGCCCATTATCTACCTATTTA
CCGCTCACCCCAGCCCATCGCCCGACCCGCTCGTGACAGATCACTACTTCTACATG
CGGGCACTCTCCTTACTCATCACCCCACCTACCATGCTCTTGGCAGCATTATCAGGC
GAATATGCTTTCGATTGGAAAAGTGGCCGAGCAAAGTCAACTATTGCAGCAATCATGA
TCCCGACGGTGTATCTGATTTGGGTAGATTATGTTGCTGTCGGTCAAGACTCTTGGTC
GATCAACGATGAGAAGATTGTAGGGTGGAGGCTTGGAGGTGTACTACCCATTGAGGA
AGCTATGTTCTTCTTACTGACGAATCTAATGATTGTTCTGGGTCTGTCTGCCTGCGAT
CATACTCAGGCCCTATACCTGCTACACGGTCGAACTATTTATGGCAACAAAAAGATGC
CATCTTCATTTCCCCTCATTACACCGCCTGTGCTCTCCCTGTTTTTTAGCAGCCGACC
ATACTCTTCTCAGCCAAAACGTGACTTGGAACTGGCAGTCAAGTTGTTGGAGGAAAA
GAGCCGGAGCTTTTTTGTTGCCTCGGCTGGATTTCCTAGCGAAGTTAGGGAGAGGC
TGGTTGGACTATACGCATTCTGCCGGGTGACTGATGATCTTATCGACTCTCCTGAAGT
ATCTTCCAACCCGCATGCCACAATTGACATGGTCTCCGATTTTCTTACCCTACTATTTG
GGCCCCCGCTACACCCTTCGCAACCTGACAAGATCCTTTCTTCGCCTTTACTTCCTC

CTTCGCACCCTTCCCGACCCACGGGAATGTATCCCCTCCCGCCTCCTCCTTCGCTCT
CGCCTGCCGAGCTCGTTCAATTCCTTACCGAAAGGGTTCCCGTTCAATACCATTTCG
CCTTCAGGTTGCTCGCTAAGTTGCAAGGGCTGATCCCTCGATACCCACTCGACGAAC
TCCTTAGAGGATACACCACTGATCTTATCTTTCCTTTATCGACAGAGGCAGTCCAGGC
TCGGAAGACGCCTATCGAGACCACAGCTGACTTGCTGGACTATGGTCTATGTGTAGC
AGGCTCAGTCGCCGAGCTATTGGTCTATGTCTCTTGGGCAAGTGCACCAAGTCAGGT
CCCTGCCACCATAGAAGAAAGAGAAGCTGTGTTAGTGGCAAGCCGAGAGATGGGAA
CTGCCCTTCAGTTGGTGAACATTGCTAGGGACATTAAAGGGGACGCAACAGAAGGG
AGATTTTACCTACCACTCTCATTCTTTGGTCTTCGGGATGAATCAAAGCTTGCGATCC
CGACTGATTGGACGGAACCTCGGCCTCAAGATTTCGACAAACTCCTCAGTCTATCTC
CTTCGTCCACATTACCATCTTCAAACGCCTCAGAAAGCTTCCGGTTCGAATGGAAGA
CGTACTCGCTTCCATTAGTCGCCTACGCAGAGGATCTTGCCAAACATTCTTATAAGGG
AATTGACCGACTTCCTACCGAGGTTCAAGCGGGAATGCGAGCGGCTTGCGCGAGCT
ACCTACTGATCGGCCGAGAGATCAAAGTCGTTTGGAAAGGAGACGTCGGAGAGAGA
AGGACAGTTGCCGGATGGAGGAGAGTACGGAAAGTCTTGAGTGTGGTCATGAGCG
GATGGGAAGGGCAGTAA

CrtE DNA sequence.

ATGGATTACGCGAACATCCTCACAGCAATTCCACTCGAGTTTACTCCTCAGGATGATA
TCGTGCTCCTTGAACCGTATCACTACCTAGGAAAGAACCCTGGAAAAGAAATTCGAT
CACAACTCATCGAGGCTTTCAACTATTGGTTGGATGTCAAGAAGGAGGATCTCGAGG
TCATCCAGAACGTTGTTGGCATGCTACATACCGCTAGCTTATTAATGGACGATGTGGA
GGATTCATCGGTCCTCAGGCGTGGGTCGCCTGTGGCCCATCTAATTTACGGGATTCC
GCAGACAATAAACACTGCAAACTACGTCTACTTTCTGGCTTATCAAGAGATCTTCAAG
CTTCGCCCAACACCGATACCCATGCCTGTAATTCCTCCTTCATCTGCTTCGCTTCAAT
CATCCGTCTCCTCTGCATCCTCCTCCTCCTCGGCCTCGTCTGAAAACGGGGGCACG
TCAACTCCTAATTCGCAGATTCCGTTCTCGAAAGATACGTATCTTGATAAAGTGATCAC
AGACGAGATGCTTTCCCTCCATAGAGGGCAAGGCCTGGAGCTATTCTGGAGAGATAG
TCTGACGTGTCCTAGCGAAGAGGAATATGTGAAAATGGTTCTTGGAAAGACGGGAGG
TTTGTTCCGTATAGCGGTCAGATTGATGATGGCAAAGTCAGAATGTGACATAGACTTT
GTCCAGCTTGTCAACTTGATCTCAATATACTTCCAGATCAGGGATGACTATATGAACCT
TCAGTCTTCTGAGTATGCCCATAATAAGAATTTTGCAGAGGACCTCACAGAAGGAAAA
TTCAGTTTTCCCACTATCCACTCGATTCATGCCAACCCCTCATCGAGACTCGTCATCA
ATACGTTGCAGAAGAAATCGACCTCTCCTGAGATCCTTCACCACTGTGTAAACTACAT
GCGCACAGAAACCCACTCATTCGAATATACTCAGGAAGTCCTCAACACCTTGTCAGG
TGCACTCGAGAGAGAACTAGGAAGGCTTCAAGGAGAGTTCGCAGAAGCTAACTCAA
AGATTGATCTTGGAGACGTAGAGTCGGAAGGAAGAACGGGGAAGAACGTCAAATTG
GAAGCGATCCTGAAAAAGCTAGCCGATATCCCTCTGTGA

tHMG1 DNA sequence.

ATGGACCAATTGGTGAAAACTGAAGTCACCAAGAAGTCTTTTACTGCTCCTGTACAAA
AGGCTTCTACACCAGTTTTAACCAATAAAACAGTCATTTCTGGATCGAAAGTCAAAAG
TTTATCATCTGCGCAATCGAGCTCATCAGGACCTTCATCATCTAGTGAGGAAGATGAT
TCCCGCGATATTGAAAGCTTGGATAAGAAAATACGTCCTTTAGAAGAATTAGAAGCATT
ATTAAGTAGTGGAAATACAAAACAATTGAAGAACAAAGAGGTCGCTGCCTTGGTTATT
CACGGTAAGTTACCTTTGTACGCTTTGGAGAAAAAATTAGGTGATACTACGAGAGCGG
TTGCGGTACGTAGGAAGGCTCTTTCAATTTTGGCAGAAGCTCCTGTATTAGCATCTGA
TCGTTTACCATATAAAAATTATGACTACGACCGCGTATTTGGCGCTTGTTGTGAAAATG
TTATAGGTTACATGCCTTTGCCCGTTGGTGTTATAGGCCCCTTGGTTATCGATGGTACA
TCTTATCATATACCAATGGCAACTACAGAGGGTTGTTTGGTAGCTTCTGCCATGCGTG
GCTGTAAGGCAATCAATGCTGGCGGTGGTGCAACAACTGTTTTAACTAAGGATGGTA
TGACAAGAGGCCCAGTAGTCCGTTTCCCAACTTTGAAAAGATCTGGTGCCTGTAAGA
TATGGTTAGACTCAGAAGAGGGACAAAACGCAATTAAAAAAGCTTTTAACTCTACATC
AAGATTTGCACGTCTGCAACATATTCAAACTTGTCTAGCAGGAGATTTACTCTTCATGA
GATTTAGAACAACTACTGGTGACGCAATGGGTATGAATATGATTTCTAAAGGTGTCGA
ATACTCATTAAAGCAAATGGTAGAAGAGTATGGCTGGGAAGATATGGAGGTTGTCTCC
GTTTCTGGTAACTACTGTACCGACAAAAAACCAGCTGCCATCAACTGGATCGAAGGT
CGTGGTAAGAGTGTCGTCGCAGAAGCTACTATTCCTGGTGATGTTGTCAGAAAAGTG

TTAAAAAGTGATGTTTCCGCATTGGTTGAGTTGAACATTGCTAAGAATTTGGTTGGAT
CTGCAATGGCTGGGTCTGTTGGTGGATTTAACGCACATGCAGCTAATTTAGTGACAG
CTGTTTTCTTGGCATTAGGACAAGATCCTGCACAAAATGTTGAAAGTTCCAACTGTAT
AACATTGATGAAAGAAGTGGACGGTGATTTGAGAATTTCCGTATCCATGCCATCCATC
GAAGTAGGTACCATCGGTGGTGGTACTGTTCTAGAACCACAAGGTGCCATGTTGGAC
TTATTAGGTGTAAGAGGCCCGCATGCTACCGCTCCTGGTACCAACGCACGTCAATTA
GCAAGAATAGTTGCCTGTGCCGTCTTGGCAGGTGAATTATCCTTATGTGCTGCCCTA
GCAGCCGGCCATTTGGTTCAAAGTCATATGACCCACAACAGGAAACCTGCTGAACCA
ACAAAACCTAACAATTTGGACGCCACTGATATAAATCGTTTGAAAGATGGGTCCGTCA
CCTGCATTAAATCCTAA

ERG10 DNA sequence.

ATGTCTCAGAACGTTTACATTGTATCGACTGCCAGAACCCCAATTGGTTCATTCCAGG
GTTCTCTATCCTCCAAGACAGCAGTGGAATTGGGTGCTGTTGCTTTAAAAGGCGCCT
TGGCTAAGGTTCCAGAATTGGATGCATCCAAGGATTTTGACGAAATTATTTTTGGTAA
CGTTCTTTCTGCCAATTTGGGCCAAGCTCCGGCCAGACAAGTTGCTTTGGCTGCCG
GTTTGAGTAATCATATCGTTGCAAGCACAGTTAACAAGGTCTGTGCATCCGCTATGAA
GGCAATCATTTTGGGTGCTCAATCCATCAAATGTGGTAATGCTGATGTTGTCGTAGCT
GGTGGTTGTGAATCTATGACTAACGCACCATACTACATGCCAGCAGCCCGTGCGGGT
GCCAAATTTGGCCAAACTGTTCTTGTTGATGGTGTCGAAAGAGATGGGTTGAACGAT
GCGTACGATGGTCTAGCCATGGGTGTACACGCAGAAAAGTGTGCCCGTGATTGGGAT
ATTACTAGAGAACAACAAGACAATTTTGCCATCGAATCCTACCAAAAATCTCAAAAATC
TCAAAAGGAAGGTAAATTCGACAATGAAATTGTACCTGTTACCATTAAGGGATTTAGAG
GTAAGCCTGATACTCAAGTCACGAAGGACGAGGAACCTGCTAGATTACACGTTGAAA
AATTGAGATCTGCAAGGACTGTTTTCCAAAAAGAAAACGGTACTGTTACTGCCGCTAA
CGCTTCTCCAATCAACGATGGTGCTGCAGCCGTCATCTTGGTTTCCGAAAAAGTTTT
GAAGGAAAAGAATTTGAAGCCTTTGGCTATTATCAAAGGTTGGGGTGAGGCCGCTCA
TCAACCAGCTGATTTTACATGGGCTCCATCTCTTGCAGTTCCAAAGGCTTTGAAACAT
GCTGGCATCGAAGACATCAATTCTGTTGATTACTTTGAATTCAATGAAGCCTTTTCGG
TTGTCGGTTTGGTGAACACTAAGATTTTGAAGCTAGACCCATCTAAGGTTAATGTATAT
GGTGGTGCTGTTGCTCTAGGTCACCCATTGGGTTGTTCTGGTGCTAGAGTGGTTGTT
ACACTGCTATCCATCTTACAGCAAGAAGGAGGTAAGATCGGTGTTGCCGCCATTTGTA
ATGGTGGTGGTGGTGCTTCCTCTATTGTCATTGAAAAGATATGA

ERG8 DNA sequence.

ATGTCAGAGTTGAGAGCCTTCAGTGCCCCAGGGAAAGCGTTACTAGCTGGTGGATAT
TTAGTTTTAGATACAAAATATGAAGCATTTGTAGTCGGATTATCGGCAAGAATGCATGC
TGTAGCCCATCCTTACGGTTCATTGCAAGGGTCTGATAAGTTTGAAGTGCGTGTGAA
AAGTAAACAATTTAAAGATGGGGAGTGGCTGTACCATATAAGTCCTAAAAGTGGCTTC
ATTCCTGTTTCGATAGGCGGATCTAAGAACCCTTTCATTGAAAAAGTTATCGCTAACGT
ATTTAGCTACTTTAAACCTAACATGGACGACTACTGCAATAGAAACTTGTTCGTTATTG
ATATTTTCTCTGATGATGCCTACCATTCTCAGGAGGATAGCGTTACCGAACATCGTGG
CAACAGAAGATTGAGTTTTCATTCGCACAGAATTGAAGAAGTTCCCAAAACAGGGCT
GGGCTCCTCGGCAGGTTTAGTCACAGTTTTAACTACAGCTTTGGCCTCCTTTTTTGTA
TCGGACCTGGAAAATAATGTAGACAAATATAGAGAAGTTATTCATAATTTAGCACAAGT
TGCTCATTGTCAAGCTCAGGGTAAAATTGGAAGCGGGTTTGATGTAGCGGCGGCAG
CATATGGATCTATCAGATATAGAAGATTCCCACCCGCATTAATCTCTAATTTGCCAGATA
TTGGAAGTGCTACTTACGGCAGTAAACTGGCGCATTTGGTTGATGAAGAAGACTGGA
ATATTACGATTAAAAGTAACCATTTACCTTCGGGATTAACTTTATGGATGGGCGATATTA
AGAATGGTTCAGAAACAGTAAAACTGGTCCAGAAGGTAAAAAATTGGTATGATTCGCA
TATGCCAGAAAGCTTGAAAATATATACAGAACTCGATCATGCAAATTCTAGATTTATGGA
TGGACTATCTAAACTAGATCGCTTACACGAGACTCATGACGATTACAGCGATCAGATAT
TTGAGTCTCTTGAGAGGAATGACTGTACCTGTCAAAAGTATCCTGAAATCACAGAAGT
TAGAGATGCAGTTGCCACAATTAGACGTTCCTTTAGAAAAATAACTAAAGAATCTGGT
GCCGATATCGAACCTCCCGTACAAACTAGCTTATTGGATGATTGCCAGACCTTAAAAG
GAGTTCTTACTTGCTTAATACCTGGTGCTGGTGGTTATGACGCCATTGCAGTGATTAC
TAAGCAAGATGTTGATCTTAGGGCTCAAACCGCTAATGACAAAAGATTTTCTAAGGTT
CAATGGCTGGATGTAACTCAGGCTGACTGGGGTGTTAGGAAAGAAAAAGATCCGGA

AACTTATCTTGATAAATAA

ERG12 DNA sequence.

ATGTCATTACCGTTCTTAACTTCTGCACCGGGAAAGGTTATTATTTTTGGTGAACACTC
TGCTGTGTACAACAAGCCTGCCGTCGCTGCTAGTGTGTCTGCGTTGAGAACCTACCT
GCTAATAAGCGAGTCATCTGCACCAGATACTATTGAATTGGACTTCCCGGACATTAGC
TTTAATCATAAGTGGTCCATCAATGATTTCAATGCCATCACCGAGGATCAAGTAAACTC
CCAAAAATTGGCCAAGGCTCAACAAGCCACCGATGGCTTGTCTCAGGAACTCGTTAG
TCTTTTGGATCCGTTGTTAGCTCAACTATCCGAATCCTTCCACTACCATGCAGCGTTTT
GTTTCCTGTATATGTTTGTTTGCCTATGCCCCCATGCCAAGAATATTAAGTTTTCTTTAA
AGTCTACTTTACCCATCGGTGCTGGGTTGGGCTCAAGCGCCTCTATTTCTGTATCACT
GGCCTTAGCTATGGCCTACTTGGGGGGGTTAATAGGATCTAATGACTTGGAAAAGCT
GTCAGAAAACGATAAGCATATAGTGAATCAATGGGCCTTCATAGGTGAAAAGTGTATT
CACGGTACCCCTTCAGGAATAGATAACGCTGTGGCCACTTATGGTAATGCCCTGCTAT
TTGAAAAAGACTCACATAATGGAACAATAAACACAAACAATTTTAAGTTCTTAGATGATT
TCCCAGCCATTCCAATGATCCTAACCTATACTAGAATTCCAAGGTCTACAAAAGATCTT
GTTGCTCGCGTTCGTGTGTTGGTCACCGAGAAATTTCCTGAAGTTATGAAGCCAATT
CTAGATGCCATGGGTGAATGTGCCCTACAAGGCTTAGAGATCATGACTAAGTTAAGTA
AATGTAAAGGCACCGATGACGAGGCTGTAGAAACTAATAATGAACTGTATGAACAACT
ATTGGAATTGATAAGAATAAATCATGGACTGCTTGTCTCAATCGGTGTTTCTCATCCTG
GATTAGAACTTATTAAAAATCTGAGCGATGATTTGAGAATTGGCTCCACAAAACTTACC
GGTGCTGGTGGCGGCGGTTGCTCTTTGACTTTGTTACGAAGAGACATTACTCAAGAG
CAAATTGACAGCTTCAAAAAGAAATTGCAAGATGATTTTAGTTACGAGACATTTGAAAC
AGACTTGGGTGGGACTGGCTGCTGTTTGTTAAGCGCAAAAAATTTGAATAAAGATCTT
AAAATCAAATCCCTAGTATTCCAATTATTTGAAAATAAAACTACCACAAAGCAACAAATT
GACGATCTATTATTGCCAGGAAACACGAATTTACCATGGACTTCATAA

mMVD1 DNA sequence.

ATGACCGTTTACACAGCATCCGTTACCGCACCCGTCAACATCGCAACCCTTAAGTATT
GGGGGAAAAGGGACACGAAGTTGAATCTGCCCACCAATTCGTCCATATCAGTGACTT
TATCGCAAGATGACCTCAGAACGTTGACCTCTGCGGCTACTGCACCTGAGTTTGAAC
GCGACACTTTGTGGTTAAATGGAGAACCACACAGCATCGACAATGAAAGAACTCAAA
ATTGTCTGCGCGACCTACGCCAATTAAGAAAGGAAATGGAATCGAAGGACGCCTCAT
TGCCCACATTATCTCAATGGAAACTCCACATTGTCTCCGAAAATAACTTTCCTACAGCA
GCTGGTTTAGCTTCCTCCGCTGCTGGCTTTGCTGCATTGGTTTCTGCAATTGCTAAGT
TATACCAATTACCACAGTCAACTTCAGAAATATCTAGAATAGCAAGAAAGGGGTCTGG
TTCAGCTTGTAGATCGTTGTTTGGCGGATACGTGGCCTGGGAAATGGGAAAAGCTGA
AGATGGTCATGATTCCATGGCAGTACAAATCGCAGACAGCTCTGACTGGCCTCAGAT
GAAAGCTTGTGTCCTAGTTGTCAGCGATATTAAAAAGGATGTGAGTTCCACTCAGGGT
ATGCAATTGACCGTGGCAACCTCCGAACTATTTAAAGAAAGAATTGAACATGTCGTAC
CAAAGAGATTTGAAGTCATGCGTAAAGCCATTGTTGAAAAAGATTTCGCCACCTTTGC
AAAGGAAACAATGATGGATTCCAACTCTTTCCATGCCACATGTTTGGACTCTTTCCCT
CCAATATTCTACATGAATGACACTTCCAAGCGTATCATCAGTTGGTGCCACACCATTAA
TCAGTTTTACGGAGAAACAATCGTTGCATACACGTTTGATGCAGGTCCAAATGCTGTG
TTGTACTACTTAGCTGAAAATGAGTCGAAACTCTTTGCATTTATCTATAAATTGTTTGGC
TCTGTTCCTGGATGGGACAAGAAATTTACTACTGAGCAGCTTGAGGCTTTCAACCATC
AATTTGAATCATCTAACTTTACTGCACGTGAATTGGATCTTGAGTTGCAAAAGGATGTT
GCCAGAGTGATTTTAACTCAAGTCGGTTCAGGCCCACAAGAAACAAACGAATCTTTG
ATTGACGCAAAGACTGGTCTACCAAAGGAATAA

ERG20 DNA sequence.

ATGGCTTCAGAAAAAGAAATTAGGAGAGAGAGATTCTTGAACGTTTTCCCTAAATTAG
TAGAGGAATTGAACGCATCGCTTTTGGCTTACGGTATGCCTAAGGAAGCATGTGACT
GGTATGCCCACTCATTGAACTACAACACTCCAGGCGGTAAGCTAAATAGAGGTTTGTC
CGTTGTGGACACGTATGCTATTCTCTCCAACAAGACCGTTGAACAATTGGGGCAAGA
AGAATACGAAAAGGTTGCCATTCTAGGTTGGTGCATTGAGTTGTTGCAGGCTTACTTC
TTGGTCGCCGATGATATGATGGACAAGTCCATTACCAGAAGAGGCCAACCATGTTGG
TACAAGGTTCCTGAAGTTGGGGAAATTGCCATCAATGACGCATTCATGTTAGAGGCT
GCTATCTACAAGCTTTTGAAATCTCACTTCAGAAACGAAAAATACTACATAGATATCAC

CGAATTGTTCCATGAGGTCACCTTCCAAACCGAATTGGGCCAATTGATGGACTTAATC
ACTGCACCTGAAGACAAAGTCGACTTGAGTAAGTTCTCCCTAAAGAAGCACTCCTTC
ATAGTTACTTTCAAGACTGCTTACTATTCTTTCTACTTGCCTGTCGCATTGGCCATGTA
CGTTGCCGGTATCACGGATGAAAAGGATTTGAAACAAGCCAGAGATGTCTTGATTCC
ATTGGGTGAATACTTCCAAATTCAAGATGACTACTTAGACTGCTTCGGTACCCCAGAA
CAGATCGGTAAGATCGGTACAGATATCCAAGATAACAAATGTTCTTGGGTAATCAACA
AGGCATTGGAACTTGCTTCCGCAGAACAAAGAAAGACTTTAGACGAAAATTACGGTA
AGAAGGACTCAGTCGCAGAAGCCAAATGCAAAAAGATTTTCAATGACTTGAAAATTGA
ACAGCTATACCACGAATATGAAGAGTCTATTGCCAAGGATTTGAAGGCCAAAATTTCT
CAGGTCGATGAGTCTCGTGGCTTCAAAGCTGATGTCTTAACTGCGTTCTTGAACAAA
GTTTACAAGAGAAGCAAATAG

mERG13 DNA sequence.

ATGAAACTCTCAACTAAACTTTGTTGGTGTGGTATTAAAGGAAGACTTAGGCCGCAAA
AGCAACAACAATTACACAATACAAACTTGCAAATGACTGAACTAAAAAAACAAAAGAC
CGCTGAACAAAAAACCAGACCTCAAAATGTCGGTATTAAAGGTATCCAAATTTACATC
CCAACTCAATGTGTCAACCAATCTGAGCTAGAGAAATTTGATGGCGTTTCTCAAGGTA
AATACACAATTGGTCTGGGCCAAACCAACATGTCTTTTGTCAATGACAGAGAAGATAT
CTACTCGATGTCCCTAACTGTTTTGTCTAAGTTGATCAAGAGTTACAACATCGACACC
AACAAAATTGGTAGATTAGAAGTCGGTACTGAAACTCTGATTGACAAGTCCAAGTCTG
TCAAGTCTGTCTTGATGCAATTGTTTGGTGAAAACACTGACGTCGAAGGTATTGACAC
GCTTAATGCCTGTTACGGTGGTACCAACGCGTTGTTCAACTCTTTGAACTGGATTGAA
TCTAACGCATGGGATGGTAGAGATGCCATTGTAGTTTGCGGTGATATTGCCATCTACG
ATAAGGGTGCCGCAAGACCAACCGGTGGTGCCGGTACTGTTGCTATGTGGATCGGT
CCTGATGCTCCAATTGTATTTGACTCTGTAAGAGCTTCTTACATGGAACACGCCTACG
ATTTTTACAAGCCAGATTTCACCAGCGAATATCCTTACGTCGATGGTCATTTTTCATTA
ACTTGTTACGTCAAGGCTCTTGATCAAGTTTACAAGAGTTATTCCAAGAAGGCTATTTC
TAAAGGGTTGGTTAGCGATCCCGCTGGTTCGGATGCTTTGAACGTTTTGAAATATTTC
GACTACAACGTTTTCCATGTTCCAACCTGTAAATTGGTCACAAAATCATACGGTAGATT
ACTATATAACGATTTCAGAGCCAATCCTCAATTGTTCCCAGAAGTTGACGCCGAATTA
GCTACTCGCGATTATGACGAATCTTTAACCGATAAGAACATTGAAAAAACTTTTGTTAA
TGTTGCTAAGCCATTCCACAAAGAGAGAGTTGCCCAATCTTTGATTGTTCCAACAAAC
ACAGGTAACATGTACACCGCATCTGTTTATGCCGCCTTTGCATCTCTATTAAACTATGT
TGGATCTGACGACTTACAAGGCAAGCGTGTTGGTTTATTTTCTTACGGTTCCGGTTTA
GCTGCATCTCTATATTCTTGCAAAATTGTTGGTGACGTCCAACATATTATCAAGGAATT
AGATATTACTAACAAATTAGCCAAGAGAATCACCGAAACTCCAAAGGATTACGAAGCT
GCCATCGAATTGAGAGAAAATGCCCATTTGAAGAAGAACTTCAAACCTCAAGGTTCC
ATTGAGCATTTGCAAAGTGGTGTTTACTACTTGACCAACATCGATGACAAATTTAGAA
GATCTTACGATGTTAAAAAATAA

mIDI1 DNA sequence.

ATGACTGCCGACAACAATAGTATGCCCCATGGTGCAGTATCTAGTTACGCCAAATTAG
TGCAAAACCAAACACCTGAAGACATTTTGGAAGAGTTTCCTGAAATTATTCCATTACA
ACAAAGACCTAATACCCGATCTAGTGAAACGTCAAATGACGAAAGCGGAGAAACATG
TTTTTCTGGTCATGATGAGGAGCAAATTAAGTTAATGAATGAAAATTGTATTGTTTTGG
ATTGGGACGATAATGCTATTGGTGCCGGTACCAAGAAAGTTTGTCATTTAATGGAAAAT
ATTGAAAAGGGTTTACTACATCGTGCATTCTCCGTCTTTATTTTCAATGAACAAGGTGA
ATTACTTTTACAACAAAGAGCCACTGAAAAAATAACTTTCCCTGATCTTTGGACTAACA
CATGCTGCTCTCATCCACTATGTATTGATGACGAATTAGGTTTGAAGGGTAAGCTAGA
CGATAAGATTAAGGGCGCTATTACTGCGGCGGTGAGAAAACTAGATCATGAATTAGGT
ATTCCAGAAGATGAAACTAAGACAAGGGGTAAGTTTCACTTTTTAAACAGAATCCATTA
CATGGCACCAAGCAATGAACCATGGGGTGAACATGAAATTGATTACATCCTATTTTATA
AGATCAACGCTAAAGAAAACTTGACTGTCAACCCAAACGTCAATGAAGTTAGAGACTT
CAAATGGGTTTCACCAAATGATTTGAAAACTATGTTTGCTGACCCAAGTTACAAGTTTA
CGCCTTGGTTTAAGATTATTTGCGAGAATTACTTATTCAACTGGTGGGAGCAATTAGAT
GACCTTTCTGAAGTGGAAAATGACAGGCAAATTCATAGAATGCTATAA

MEL1 DNA sequence.

atgtttgctttctactttctcaccgcatgcatcagtttgaagggcgtttttggggtgtctccgagttacaatggccttggtctcactcc

acagatgggttgggacaactggaatacgtttgcctgcgatgtcagtgaacagctacttctagacaccgctgatagaatttctg
acttggggctaaaggatatgggttacaagtatatcattctggatgactgctggtctagcggcagagattccgacggtttcctcgt
tgcagatgaacaaaaatttcccaatggtatgggccatgttgcagaccacctgcataataacagctttcttttcggtatgtattcgt
ctgctggtgagtacacctgtgctggatatcctgggtctctgggtcgtgaggaagaagatgcacagttctttgcaaataaccgc
gttgactacttgaagtacgataattgttacaataagggtcagtttggtacaccggaaatttcttaccaccgttacaaggccatgt
cagatgctttgaataaaactggtaggcctatattctattctctatgtaactggggtcaggatttaacattttactggggctctggtat
cgccaattcttggagaatgagtggagatgttactgctgagttcactcgtccagatagcagatgtccctgtgatggcgatgaata
cgattgcaagtacgccggtttccattgttctattatgaatattcttaacaaggcagctccaatggggcaaaatgcaggtgttggt
ggttggaatgatctggacaatctagaggttggtgtcgggaatttgactgacgatgaggaaaaggcacatttctctatgtgggc
aatggtaaagtctccacttatcattggtgccaatgtgaataacttaaaggcatcttcgtactcaatctatagtcaagcctctgtcat
cgcaattaatcaagattcaaatggtattccagcaacaagagtctggagatattatgtttcagacacagatgaatatggacaa
ggtgaaattcaaatgtggagtggtcctcttgacaatggtgatcaagtggttgctttattgaatggaggaagcgtatctagacca
atgaacacgaccttggaagagattttttttgacagcaatctgggttcaaagaaactgacatcgacttgggatatctacgaccta
tgggccaacagagttgacaactcgacagcgtctgctatccttggacggaataagacagccaccggtattctctacaatgcta
cggagcaatcctacaaagacggtttgtctaagaatgatacaagactgtttggtcagaaaattggtagtctttctccaaatgctat
acttaacacgactgttccagctcacggtatcgccttctataggttgagaccctcttcttga

Table S5. F1~F7 sequence

fragment F1

<u>TCCTCTTCAGAGTACAGAAGATTAAGTGAAACGCAGCGTTTTCTGACGGTA</u>CTAGA
GGACTCTTAGGGGAAGGTAGAATCAATAAAGATCATATTAGGTAAGCAAATTTTGGATG
GAATAGGAGACTAGGTGTGGATGCGCGATCTCGCCAAATTGCACGACCAGAGTGGA
TGCCGGATGGTGGTAAACCgtttcttccttttta CCACCCAAGTGCGAGTGAAACACCCCATG
GCTGCTCTCCGATTGCCCCTCTACAGGCATAAGGGTGTGACTTTGTGGGCTTGAATT
TTACACCCCCTCCAACTTTTCTCGCATCAATTGATCCTGTTACCAATATTGCATGCCCG
GAGGAGACTTGCCCCCTAATTTCGCGGCGTCGTCCCGGATCGCAGGGTGAGACTGT
AGAGACCCCACATAGTGAcaatgattatgtaagaAGAGGGGGGTGATTCGGCCGGCTATCG
AACTCTAACAACTAGGGGGGTGAACAATGCCCAGCAGTCCTCCCCACTCTTTGACAA
ATCAGTATCACCGATTAACACCCCAAATCTTATTCTCAACGGTCCCTCATCCTTGCAC
CCCTCTTTGGACAAATGGCAGTTAGCATTGGTGCACTGACTGACTGCCCAACCTTAA
ACCCAAATTTCTTAGAAGGGGCCCATCTAGTTAGCGAGGGGTGAAAAATTCCTCCAT
CGGAGATGTATTGACCGTAAGTTGCTGCTTAAAAAAAATCAGTTCAGATAGCGagacttttt
tgatttcgCAACGGGAGTGCCTGTTCCATTCGATTGCAATTCTCACCCCTTCTGCCCAGT
CCTGCCAATTGCCCATGAATCTGCTAATTTCGTTGATTCCCACCCCCCTTTCCAACTC
CACAAATTGTCCAATCTCGTTTTCCATTTGGGAGAATCTGCATGTCGACTACATAAAG
CGACCGGTGTCCGAAAAGATCTGTGTagttttcaacattttgtGCTCCCCCCGCTGTTTGAAAA
CGGGGGTGAGCGCTCTCCGGGGTGCGAATTCGTGCCCAATTCCTTTCACCCTGCCT
ATTGTAGACGTCAACCCGCATCTGGTGCGAATATAGCGCACCCCCAATGATCACACC
AACAATTGGTCCACCCCTCCCCAATCTCTAATATTCACAATTCACCTCACTATAAATAC
CCCTGTCCTGCTCccaaattcttttttccttcttccaTCAGCTACTAGCTTTTATCTTATTTACTTTAC
GAAAgATGGATTACGCGAACATCCTCACAGCAATTCCACTCGAGTTTACTCCTCAGGA
TGATATCGTGCTCCTTGAACCGTATCACTACCTAGGAAAGAACCCTGGAAAAGAAATT
CGATCACAACTCATCGAGGCTTTCAACTATTGGTTGGATGTCAAGAAGGAGGATCTC
GAGGTCATCCAGAACGTTGTTGGCATGCTACATACCGCTAGCTTATTAATGGACGATG
TGGAGGATTCATCGGTCCTCAGGCGTGGGTCGCCTGTGGCCCATCTAATTTACGGGA
TTCCGCAGACAATAAACACTGCAAACTACGTCTACTTTCTGGCTTATCAAGAGATCTT
CAAGCTTCGCCCAACACCGATACCCATGCCTGTAATTCCTCCTTCATCTGCTTCGCTT
CAATCATCCGTCTCCTCTGCATCCTCCTCCTCCTCGGCCTCGTCTGAAAACGGGGG
CACGTCAACTCCTAATTCGCAGATTCCGTTCTCGAAAGATACGTATCTTGATAAAGTGA
TCACAGACGAGATGCTTTCCCTCCATAGAGGGCAAGGCCTGGAGCTATTCTGGAGA
GATAGTCTGACGTGTCCTAGCGAAGAGGAATATGTGAAAATGGTTCTTGGAAAGACG
GGAGGTTTGTTCCGTATAGCGGTCAGATTGATGATGGCAAAGTCAGAATGTGACATA
GACTTTGTCCAGCTTGTCAACTTGATCTCAATATACTTCCAGATCAGGGATGACTATAT
GAACCTTCAGTCTTCTGAGTATGCCCATAATAAGAATTTTGCAGAGGACCTCACAGAA
GGAAAATTCAGTTTTCCCACTATCCACTCGATTCATGCCAACCCCTCATCGAGACTCG
TCATCAATACGTTGCAGAAGAAATCGACCTCTCCTGAGATCCTTCACCACTGTGTAAA

CTACATGCGCACAGAAACCCACTCATTCGAATATACTCAGGAAGTCCTCAACACCTTG<br>TCAGGTGCACTCGAGAGAGAACTAGGAAGGCTTCAAGGAGAGTTCGCAGAAGCTAA<br>CTCAAAGATTGATCTTGGAGACGTAGAGTCGGAAGGAAGAACGGGGAAGAACGTCA<br>AATTGGAAGCGATCCTGAAAAAGCTAGCCGATATCCCTCTGTagcACGGGAAGTCTTT<br>ACAGTTTTAGTTAGGAGCCCTTATATATGACAGTAATGCTAGTACGTTTTGTTTTGTTTA<br>ATTAATAACTTAGTTTATGTTAGCCTAGTATAGACTCCATCAATTTTTTTTGTTATTACGT<br>AAGCCGCGATGATAATATCTGATGAAAAATTCCTATCAGAAAATAATTTATCAAAAGTTT<br>CATGCGATATGAGACTAAGTAGAATAGGGAC***TCCCAAAGTGTCAGTCACAAGGGTC***<br>***GCCTCCAAAAAGGCAGATCTCATCG***<br>The promoter P_{ADH2} was marked yellow, *CrtE* was marked blue, T_{DAS1} was marked green, and the underlined parts were the flanking homologous sequences used for assembly
fragment F2
GCTCACAGCGTACACATCACCACTCATTTCTCTATTGAACGGcttgaaatttggaaaccagat<br>gaaaaataaaaggaatggaagaagaatgAGAAAAGGATAATTAATCTTTGGTTTAGCTAAATTCTT<br>CATTGCACTTTGACCTTAAAGGGGCTGATTTAAGGTTATGCCggggaagaagaaatagcGC<br>GATGAGCAAAGTCGATGCCTAAAGGAGTGGTTTTGCTACCTCATTTAAGAAGAGAATA<br>GGACGTGCATCCAGCGATGCGTGCTAGGACAAAGAACCGCACTTGGCGGGTACAAA<br>CCTGACGTCATTTCCTGATATTATTGACATTTGAGCTGACCAATTAAGGTGCCCATCCA<br>CAATAGCCACCTGGATAGCGGAATGCACCCCCATTGAGTTGATCAAACTACCATTTTG<br>CTTATACCTCAAGTTAATGTTGAACTACCATTCTTCACATGCTCCTCCTAGATCCCCTG<br>TCCCCTTTCTCCCCCTCTTTCATCCTTTAATTTGCATTTCTTGACGGTCTTCTATCCCT<br>AGAAAGTTTGGAACGCCTGCTATATGGTTAGGACACGACTGACTAGCTAtaaaatttttcaga<br>ccagactctttctcttcttaaCGCAAATTTAACAGGCAGACAACAACATAGGAAAGAATCACCATA<br>TAGGTTGGACTCTTTACAGACGTCCTTGGCCGTTGACCATGGTGGTACAGTTGTCCA<br>AGTTCTACAAGTTTGTCTGAAGAATGAAGTTATTGGTCTTGGGTGCAGCTTTCCATCT<br>GTTCGATTTATTCGGCTAAGAGTTTACCATTGTGTGCTCGTATGGGGAAGGGTGCAA<br>GGATCAGTAATACAGTCGAACCTGGAGTATCTACCATAGTGGGGATACAATGTAGTTTA<br>TCTGTTATCTCGATTGTTCCTAATTAAggttttctttgatccTCTTCTAGTCCACACCTCCTAGAT<br>GACATTCGAGCTGCCTGGATTGGATGCCTAGGTTTATTGCCTAGTTCAATACAATTCG<br>TGCGGGCTACAGTAGAAGGCCCTTACATAATCCGGAAAGCATGGTCCCCCACCAAAT<br>TGAGAGCTTTTTCAGCCTTCACTGGTGGTATCATTTTCGGGAGATAATAAGGTTTCGA<br>TTGGGAATTCCCACCAGAGAACACTATAGAGGGACCAAGCTGATGCTAGCCTGACAT<br>CCCCAAAGCACACTTCGTAATTGAAAACCGTTACCTCTAGCACACTGTCCAGACTAC<br>CCCCGTCAAAAAAACGCTCTTTTTCTCGACTAATTGAGTCTTCAACTCATCCCGTCCT<br>TGCCGAATTACTTGAATTCATTTCACACCTCCGTTGCTTACGTACTCTCACCGGTCTC<br>CGGTGTACATGGATCCGCTATTGCCAGATATTTCTCATACAACAATCACCAGATCAAG<br>GTCGTGAACGGACCAATGGCATCCAGAGCAATCCTGAACAGATAGGGGTCCGGGCT<br>GTATAAAGTGAAATAACGTGACTTGAACCAGCAACTATGTCCCAGTTGTGCTACACTT<br>AACACGCGATTACCCCGGAGCTCACCAGGCCTCTTCCCCCTCTCATTGGAACCCTC<br>CTAGCGCTTCGAAATAATGGCTGCGTACTATTTAACTGGTGCCAGTTCCCGCTGACAA<br>TatcctttttcttctcccCTTAGTTCCCCACATATCAATTGAACATATTTTTTACACAgATGGACCA<br>ATTGGTGAAAACTGAAGTCACCAAGAAGTCTTTTACTGCTCCTGTACAAAAGGCTTCT<br>ACACCAGTTTTAACCAATAAAACAGTCATTTCTGGATCGAAAGTCAAAAGTTTATCATC<br>TGCGCAATCGAGCTCATCAGGACCTTCATCATCTAGTGAGGAAGATGATTCCCGCGA<br>TATTGAAAGCTTGGATAAGAAAATACGTCCTTTAGAAGAATTAGAAGCATTATTAAGTA<br>GTGGAAATACAAAACAATTGAAGAACAAAGAGGTCGCTGCCTTGGTTATTCACGGTA<br>AGTTACCTTTGTACGCTTTGGAGAAAAAATTAGGTGATACTACGAGAGCGGTTGCGGT<br>ACGTAGGAAGGCTCTTTCAATTTTGGCAGAAGCTCCTGTATTAGCATCTGATCGTTTA<br>CCATATAAAAATTATGACTACGACCGCGTATTTGGCGCTTGTTGTGAAAATGTTATAGG<br>TTACATGCCTTTGCCCGTTGGTGTTATAGGCCCCTTGGTTATCGATGGTACATCTTATC<br>ATATACCAATGGCAACTACAGAGGGTTGTTTGGTAGCTTCTGCCATGCGTGGCTGTAA<br>GGCAATCAATGCTGGCGGTGGTGCAACAACTGTTTTAACTAAGGATGGTATGACAAG<br>AGGCCCAGTAGTCCGTTTCCCAACTTTGAAAAGATCTGGTGCCTGTAAGATATGGTTA<br>GACTCAGAAGAGGGACAAAACGCAATTAAAAAAGCTTTTAACTCTACATCAAGATTTG<br>CACGTCTGCAACATATTCAAACTTGTCTAGCAGGAGATTTACTCTTCATGAGATTTAGA<br>ACAACTACTGGTGACGCAATGGGTATGAATATGATTTCTAAAGGTGTCGAATACTCATT<br>AAAGCAAATGGTAGAAGAGTATGGCTGGGAAGATATGGAGGTTGTCTCCGTTTCTGG

TAACTACTGTACCGACAAAAAACCAGCTGCCATCAACTGGATCGAAGGTCGTGGTAA
GAGTGTCGTCGCAGAAGCTACTATTCCTGGTGATGTTGTCAGAAAAGTGTTAAAAAG
TGATGTTTCCGCATTGGTTGAGTTGAACATTGCTAAGAATTTGGTTGGATCTGCAATG
GCTGGGTCTGTTGGTGGATTTAACGCACATGCAGCTAATTTAGTGACAGCTGTTTTCT
TGGCATTAGGACAAGATCCTGCACAAAATGTTGAAAGTTCCAACTGTATAACATTGAT
GAAAGAAGTGGACGGTGATTTGAGAATTTCCGTATCCATGCCATCCATCGAAGTAGGT
ACCATCGGTGGTGGTACTGTTCTAGAACCACAAGGTGCCATGTTGGACTTATTAGGT
GTAAGAGGCCCGCATGCTACCGCTCCTGGTACCAACGCACGTCAATTAGCAAGAATA
GTTGCCTGTGCCGTCTTGGCAGGTGAATTATCCTTATGTGCTGCCCTAGCAGCCGGC
CATTTGGTTCAAAGTCATATGACCCACAACAGGAAACCTGCTGAACCAACAAAACCTA
ACAATTTGGACGCCACTGATATAAATCGTTTGAAAGATGGGTCCGTCACCTGCATTAA
ATCCTAAtagcTCAAGAGGATGTCAGAATGCCATTTGCCTGAGAGATGCAGGCTTCATT
TTTGATTACTTTTTTATTTGTAACCTATATAGTATAGGattttttttgtcattttgtttcttctcGTACGAGC
TTGCTCCTGATCAGCCTATCTCGCAGCTGATGAATATCTTGTGGTAGGGGTTTGGGAA
AATCATTCGAGtttgatgtttttcttgGTATTTCCCAC***TCCTCTTCAGAGTACAGAAGATTAAGT
GAAACGCAGCGTTTTCTGACGGTA***

The promoter P_{FBA1} was marked yellow, *tHMG1* was marked blue, T_{AOX1} was marked green, and the underlined parts were the flanking homologous sequences used for assembly.

fragment F3

AGGAACCAGTTTACGAACCCCGTCTGCTCAAACGAGTGGAGAGGGAAATCGATTC
AGCAGTTAAATCAATGCTGGAAAATATTCGAGATTACCTAATCGGATCTGGAACTTACT
TCGACCTGACATTTTCTTGCCTGGGGAGCCACGATCGATTATGTAATCAAGAATATGG
ACAGAGGGAAACAGATTTAGCTGTCAAAAGCCCAAGAGAAGCTACCGATCAATGGAT
GCGGATAGATAAAGAAAAgtcctttttttttcattaGCCATCCGAGTTGTCCAATCAAATGTCTGC
CTGCTACGCTGGAGAGGAATCACGCGTGTTTAACATTCGGATTGTCGCCTAAAATAA
GCCTATTACCTACACAGTAAAACCCGGGGGGTGCTTTGGTATCAATGACCCCGGGAT
TTTATCCACcagttttttttctttctggCAAGAGTGCATTGCATCCCCGTACAAATAGTAGCAACCT
CCACAAGAGGAATCCCCTATGAGCGAGAAGTCCATAGTAATACCCCCGCGGAAAAGA
GATATTTTGTTTCCGTGTTGCCCTTGAACTTCAGTTTCCCCCATCAGTTTATATAGTAG
CCGGGTTCCCAATCTCTAGCCCTTCTTTCCTCCTATTTCATTCCTCTCTTCTTACGTTA
TCTTACATTAGCgATGATGACGGCTCTCGCATATTACCAGATCCATCTGATCTATACTCT
CCCAATTCTTGGTCTTCTCGGTCTGCTCACTTCCCCGATTTTGACAAAATTTGACATC
TACAAAATATCGATCCTCGTATTTATTGCGTTTAGTGCAACCACACCATGGGACTCATG
GATCATCAGAAATGGCGCATGGACATATCCATCAGCGGAGAGTGGCCAAGGCGTGTT
TGGAACGTTTCTAGATGTTCCATATGAAGAGTACGCTTTCTTTGTCATTCAAACCGTAA
TCACCGGCTTGGTCTACGTCTTGGCAACTAGGCACCTTCTCCCATCTCTCGCGCTTC
CCAAGACTAGATCGTCCGCCCTTTCTCTCGCGCTCAAGGCGCTCATCCCTCTGCCCA
TTATCTACCTATTTACCGCTCACCCCAGCCCATCGCCCGACCCGCTCGTGACAGATC
ACTACTTCTACATGCGGGCACTCTCCTTACTCATCACCCCACCTACCATGCTCTTGGC
AGCATTATCAGGCGAATATGCTTTCGATTGGAAAAGTGGCCGAGCAAAGTCAACTATT
GCAGCAATCATGATCCCGACGGTGTATCTGATTTGGGTAGATTATGTTGCTGTCGGTC
AAGACTCTTGGTCGATCAACGATGAGAAGATTGTAGGGTGGAGGCTTGGAGGTGTA
CTACCCATTGAGGAAGCTATGTTCTTCTTACTGACGAATCTAATGATTGTTCTGGGTCT
GTCTGCCTGCGATCATACTCAGGCCCTATACCTGCTACACGGTCGAACTATTTATGGC
AACAAAAAGATGCCATCTTCATTTCCCCTCATTACACCGCCTGTGCTCTCCCTGTTTT
TTAGCAGCCGACCATACTCTTCTCAGCCAAAACGTGACTTGGAACTGGCAGTCAAGT
TGTTGGAGGAAAAGAGCCGGAGCTTTTTTGTTGCCTCGGCTGGATTTCCTAGCGAA
GTTAGGGAGAGGCTGGTTGGACTATACGCATTCTGCCGGGTGACTGATGATCTTATC
GACTCTCCTGAAGTATCTTCCAACCCGCATGCCACAATTGACATGGTCTCCGATTTTC
TTACCCTACTATTTGGGCCCCCGCTACACCCTTCGCAACCTGACAAGATCCTTTCTTC
GCCTTTACTTCCTCCTTCGCACCCTTCCCGACCCACGGGAATGTATCCCCTCCCGCC
TCCTCCTTCGCTCTCGCCTGCCGAGCTCGTTCAATTCCTTACCGAAAGGGTTCCCGT
TCAATACCATTTCGCCTTCAGGTTGCTCGCTAAGTTGCAAGGGCTGATCCCTCGATAC
CCACTCGACGAACTCCTTAGAGGATACACCACTGATCTTATCTTTCCTTTATCGACAG
AGGCAGTCCAGGCTCGGAAGACGCCTATCGAGACCACAGCTGACTTGCTGGACTAT
GGTCTATGTGTAGCAGGCTCAGTCGCCGAGCTATTGGTCTATGTCTCTTGGGCAAGT

GCACCAAGTCAGGTCCCTGCCACCATAGAAGAAAGAGAAGCTGTGTTAGTGGCAAG
CCGAGAGATGGGAACTGCCCTTCAGTTGGTGAACATTGCTAGGGACATTAAAGGGG
ACGCAACAGAAGGGAGATTTTACCTACCACTCTCATTCTTTGGTCTTCGGGATGAATC
AAAGCTTGCGATCCCGACTGATTGGACGGAACCTCGGCCTCAAGATTTCGACAAACT
CCTCAGTCTATCTCCTTCGTCCACATTACCATCTTCAAACGCCTCAGAAAGCTTCCGG
TTCGAATGGAAGACGTACTCGCTTCCATTAGTCGCCTACGCAGAGGATCTTGCCAAA
CATTCTTATAAGGGAATTGACCGACTTCCTACCGAGGTTCAAGCGGAATGCGAGCG
GCTTGCGCGAGCTACCTACTGATCGGCCGAGAGATCAAAGTCGTTTGGAAAGGAGA
CGTCGGAGAGAGAAGGACAGTTGCCGGATGGAGGAGAGTACGGAAAGTCTTGAGT
GTGGTCATGAGCGGATGGGAAGGGCAGTAAtagcGTAGTAGTAGTAGTAGCAGAGTAT
CTACAGTGGTGTGTATAATGTATATGAGTGTACTTACCAACCAAATTCCGTTTAGTATTT
CGTCGACGATGATGTAGTACGAGTCCTTCGCGAATCCGTTACTCTCAAGACGGGGAA
AAAAAACGACGAAAATGACCAACTTACTCAACTAAGCAAACCTCAAGAAACATAACAC
TTTGTTGTGAGACAGTAATAAAAA***GCTCACAGCGTACACATCACCACTCATTTCTCTA***
TTGAACGGcttgaaatttgg

The promoter P_{FBP1} was marked yellow, *CrtYB* was marked blue, T_{FBA1} was marked green, and the underlined parts were the flanking homologous sequences used for assembly.

fragment F4

GATCATCTTAGAGAATGTCAGTGAGGATTTTTGTAGAAATGTCTTGGTGTCCTCGTC
CAATCAGGTAGCCATCTCTGAAATATCTGGCTCCGTTGCAACTCCGAACGACCTGCT
GGCAACGTAAAATTCTCCGGGGTAAAACTTAAATGTGGAGTAATGGAACCAGAAACAT
CTCTTCCCTTCTCTCTCCTTCCACCGCCCGTTACCGTCCCTAGGAAATTTTACTCTGC
TGGAGAGCTTCTTCTACGGCCCCCTTGCAGCAATGCTCTTCCCAGCATTACGTTGCG
GGTAAAACGGAGGTCGTGTACCCGACCTAGCAGCCCAGGGATGGAAAAGTCCCGG
CCGTCGCTGGCAATAATAGCGGGCGGACGCATGTCATGAGATTATTGGAAACCACCA
GAATCGAATATAAAAGGCGAACACCTTTCCCAATTTTGGTTTCTCCTGACCCAAAGAC
TTTAAATTTAATTTATTTGTCCCTAtttcaatcaattgaacaaCTATCAAAACACAgatgATGGGAA
AAGAACAAGATCAGGATAAACCCACAGCTATCATCGTGGGATGTGGTATCGGTGGAA
TCGCCACTGCCGCTCGTCTTGCTAAAGAAGGTTTCCAGGTCACGGTGTTCGAGAAG
AACGACTACTCCGGAGGTCGATGCTCTTTAATCGAGCGAGATGGTTATCGATTCGATC
AGGGGCCCAGTTTGCTGCTCTTGCCAGATCTCTCCAAGCAGACATTCGAAGATTTGG
GAGAGAAGATGGAAGATTGGGTCGATCTCATCAAGTGTGAACCCAACTATGTTTGCC
ACTTCCACGATGAAGAGACTTTCACTCTTTCAACCGACATGGCGTTGCTCAAGCGGG
AAGTCGAGCGTTTTGAAGGCAAAGATGGATTTGATCGGTTCTTGTCGTTTATCCAAGA
AGCCCACAGACATTACGAGCTTGCTGTCGTTCACGTCCTGCAGAAGAACTTCCCTG
GCTTCGCAGCATTCTTACGGCTACAGTTCATTGGCCAAATCCTGGCTCTTCACCCCTT
CGAGTCTATCTGGACAAGAGTTTGTCGATATTTCAAGACCGACAGATTACGAAGAGTC
TTCTCGTTTGCAGTGATGTACATGGGTCAAAGCCCATACAGTGCGCCCGGAACATATT
CCTTGCTCCAATACACCGAATTGACCGAGGGCATCTGGTATCCGAGAGGAGGCTTTT
GGCAGGTTCCTAATACTCTTCTTCAGATCATCAAGCGCAACAATCCCTCAGCCAAGTT
CAATTTCAACGCTCCAGTTTCCCAGGTTCTTCTCTCTCCTGCCAAGGACCGAGCGAC
TGGTGTTCGACTTGAATCCGGCGAGGAACATCACGCCGATGTTGTGATTGTCAATGC
TGACCTCGTTTACGCCTCCGAGCACTTGATTCCTGACGATGCCAGAAACAAGATTGG
CCAACTGGGTGAAGTCAAGAGAAGTTGGTGGGCTGACTTAGTTGGTGGAAAGAAGC
TCAAGGGAAGTTGCAGTAGTTTGAGCTTCTACTGGAGCATGGACCGAATCGTGGAC
GGTCTGGGCGGACACAATATCTTCTTGGCCGAGGACTTCAAGGGATCATTCGACACA
ATCTTCGAGGAGTTGGGTCTCCCAGCCGATCCTTCCTTTTACGTGAACGTTCCCTCG
CGAATCGATCCTTCTGCCGCTCCCGAAGGCAAAGATGCTATCGTCATTCTTGTGCCG
TGTGGCCATATCGACGCTTCGAACCCTCAAGATTACAACAAGCTTGTTGCTCGGGCA
ATGAAGTTTGTGATCCACACGCTTTCCGCCAAGCTTGGACTTCCCGACTTTGAAAAA
ATGATTGTGGCAGAGAAGGTTCACGATGCTCCCTCTTGGGAGAAAGAATTCAACCTC
AAGGACGGAAGCATCTTGGGACTGGCTCACAACTTTATGCAAGTTCTTGGTTTCAGG
CCGAGCACCAGACATCCCAAGTATGACAAGTTGTTCTTTGTCGGGGCTTCGACTCAT
CCCGGAACTGGGGTTCCCATCGTCTTGGCTGGAGCCAAGTTAACTGCCAACCAAGT
TCTCGAATCCTTTGACCGATCCCCAGCTCCAGATCCCAATATGTCACTCTCCGTACCA
TATGGAAAACCTCTCAAATCAAATGGAACGGGTATCGATTCTCAGGTCCAGCTGAAGT

TCATGGATTTGGAGAGATGGGTATACCTTTTGGTATTGTTGATTGGGGCCGTGATCGC
TCGATCCGTTGGTGTTCTTGCTTTCTGAtagcGTAGATTTGGCCACTAACGGGTTAGTA
GTTGTGTAAGTCTATTaaatttgatttttgtTTATGGATGATCATCGTAGTGGCTATCTGTTTACC
TGTAGGACATCCTAGGGTGGGATGGTGATGTACACCCCCTCAATCTTCAGATGCAAC
ACTATGTGGTAGGTCATTGACATAAGGTTTAGGAAAGACCTGTTTTTTGACCAATAAAT
GGAACAGGAAGGAAAGG***AGGAACCAGTTTACGAACCCCGTCTGCTCAAACGAGTG***
GAGAGGGAAATC

The promoter P_{GAP} was marked yellow, *CrtI* was marked blue, T_{DAS2} was marked green, and the underlined parts were the flanking homologous sequences used for assembly.

fragment F5

GCACTCTGCTAACTGTAGATTAACGGCGCTCGACAACCCTTAATTCGAGATAAGCT
GGGGGAACATTCGCGAAAATGAAACAAGTCGGCTGTTATAGTATATTTATTATAATATT
GAAAGATCTCAAAAGACTACTTATTTTTGAATGAACCAAGTATGAAATCAACCTATTTG
GGGTTGACCAAAATAAGTAAATATTAATTGTCGAaagcacagatgcttcgttCGCAATGTTTCTA
CTCCTTTTTTACTCTTCCAGATTTTCTCGGACTCCGCGCATCGCCGTACCACTTCAAA
ACACCCAAGCACAGCATACTAAATTTTCCCTCTTTCTTCCTCTAGGGTGTCGTTAATTA
CCCGTACTAAAGGTTTGGAAAAGAAAAAAGGGACCGCCTCGTTTCTTTTTCTTCGTC
GAAAAAGGCAATAAAAATTTTTATCACGTTTCTTTTTCTTGAAATTTTTTTTTTTAGTTTT
TTTCTCTTTCAGTGACCTCCATTGATATTTAAGTTAATAAACGGTCTTCAATTTCTCAAG
TTTCAGTTTCATTTTTCTTGTTCTATTACAACTTTTTTTACTTCTTGTTCATTAGAAAGAA
AGCATAGCAATCTAATCTAAGGGGCGGTGTTGACAATTAATCATCGGCATAGTATATCG
GCATAGTATAATACGACAAGGTGAGGAACTAAACCATGGGTAAGGAAAAGACTCACGT
TTCGAGGCCGCGATTAAATTCCAACATGGATGCTGATTTATATGGGTATAAATGGGCTC
GCGATAATGTCGGGCAATCAGGTGCGACAATCTATCGATTGTATGGGAAGCCCGATG
CGCCAGAGTTGTTTCTGAAACATGGCAAAGGTAGCGTTGCCAATGATGTTACAGATG
AGATGGTCAGACTAAACTGGCTGACGGAATTTATGCCTCTTCCGACCATCAAGCATTT
TATCCGTACTCCTGATGATGCATGGTTACTCACCACTGCGATCCCCGGCAAAACAGC
ATTCCAGGTATTAGAAGAATATCCTGATTCAGGTGAAAATATTGTTGATGCGCTGGCA
GTGTTCCTGCGCCGGTTGCATTCGATTCCTGTTTGTAATTGTCCTTTTAACAGCGATC
GCGTATTTCGCCTCGCTCAGGCGCAATCACGAATGAATAACGGTTTGGTTGATGCGA
GTGATTTTGATGACGAGCGTAATGGCTGGCCTGTTGAACAAGTCTGGAAAGAAATGC
ATAAGCTTTTGCCATTCTCACCGGATTCAGTCGTCACTCATGGTGATTTCTCACTTGAT
AACCTTATTTTTGACGAGGGGAAATTAATAGGTTGTATTGATGTTGGACGAGTCGGAA
TCGCAGACCGATACCAGGATCTTGCCATCCTATGGAACTGCCTCGGTGAGTTTTCTC
CTTCATTACAGAAACGGCTTTTTCAAAAATATGGTATTGATAATCCTGATATGAATAAAT
TGCAGTTTCATTTGATGCTCGATGAGTTTTTCTAACACGTCCGACGGCGGCCCACGG
GTCCCAGGCCTCGGAGATCCGTCCCCCTTTTCCTTTGTCGATATCATGTAATTAGTTA
TGTCACGCTTACATTCACGCCCTCCCCCCACATCCGCTCTAACCGAAAAGGAAGGAG
TTAGACAACCTGAAGTCTAGGTCCCTATTTATTTTTTTATAGTTATGTTAGTATTAAGAA
CGTTATTTATATTTCAAATTTTTCTTTTTTTTCTGTACAGACGCGTGTACGCATGTAACA
TTATACTGAAAACCTTGCTTGAGAAGGTTTTGGGACGCTCGAAGGCTTTAATTTGCTT
GAGATCCTTTTTTTCTGCGCGTAATCTGCTGCTTGCAAACAAAAAAACCACCGCTACC
AGCGGTGGTTTGTTTGCCGGATCAAGAGCTACCAACTCTTTTTCCGAAGGTAACTGG
CTTCAGCAGAGCGCAGATACCAAATACTGTTCTTCTAGTGTAGCCGTAGTTAGGCCAC
CACTTCAAGAACTCTGTAGCACCGCCTACATACCTCGCTCTGCTAATCCTGTTACCAG
TGGCTGCTGCCAGTGGCGATAAGTCGTGTCTTACCGGGTTGGACTCAAGACGATAG
TTACCGGATAAGGCGCAGCGGTCGGGCTGAACGGGGGGTTCGTGCACACAGCCCA
GCTTGGAGCGAACGACCTACACCGAACTGAGATACCTACAGCGTGAGCTATGAGAAA
GCGCCACGCTTCCCGAAGGGAGAAAGGCGGACAGGTATCCGGTAAGCGGCAGGGT
CGGAACAGGAGAGCGCACGAGGGAGCTTCCAGGGGGAAACGCCTGGTATCTTTATA
GTCCTGTCGGGTTTCGCCACCTCTGACTTGAGCGTCGATTTTTGTGATGCTCGTCAG
GGGGGCGGAGCCT***ATGGAAAAAGCTGGAGCTGGCCTTGTCCAGATGTAATCACCG***
CCGCTGAA

The promoter P_{TEF1} was marked yellow, promoter P_{EM7} was marked purple, *KanMX* was marked blue, T_{CYC1} was marked green, *Ori* was marked red, *PARS1* was marked brown and the underlined parts were the flanking homologous sequences used for assembly.

fragment F6

ATGGAAAAAGCTGGAGCTGGCCTTGTCCAGATGTAATCACCGCCGCTGAATGCCT
GCACTGGGTAGACCCACAGAAATTCGTTGCAAATGTCGCCAATTCTCTAAGGGATCA
TGGCACCTTGAGTTACTGGCTTTATACAgaaccaatttttcagaacgAAAGGGCCAATCAGGT
TTACAACAAATTCACCTACGGGTCTGACTACCTGGGCCCATATTGGGATCCTGGCCG
TACACATTTCAGAAATCATTTAAAGGAGTTGAATCACATCTTACTGGATAGCGAGCTTT
TTGACGAAGTGAAAATTTCTAATTTTAAACAAGAGGAAGGGGTCAAAAACGGAGATAT
CTTATACttggaaaaagagatgACAATCAgtgatttcatcaattttgtATCTAGTTGGCCTTCTGTGTTTT
CGTGGAAGCAGCAACGAGGAAAGGAGGGTATCCTAGATGATTTTTACAACGAACTGA
ACGACTGCTTTGAGGGGGGTAACATGAAAGTAATATGGAACTCCGTCCTAGTATTTGC
CAGGAGGAAGCAAAGGGTTGTATAGGCTTTAGTACTTATAGAGGAAACGGGGTTACG
TGCAAGCGCGCATGCCTGAGCTTTGAGGGGGGGGACTTTCAcatctcttcttctcaCACTTA
GCCCTAACACAGAGAATAATAAAAAGCATTGCAAGATGAGTGTTGTCAGCAAGCAATA
CGACATCCACGAAGGCATTATCTTTGTAATTGAATTGACCCCGGAGCTTCACGCGCC
GGCTTCAGAAGGGAAATCTCAGCTCCA***GATCATCTTAGAGAATGTCAGTGAGGATTT***
TTGTAGAAATGTCTTGGTGTCCTCGTCC

The upstream homologous arm of *KpKu70* was marked gray and the underlined parts were the flanking homologous sequences used for assembly.

fragment F7

TCCCAAAGTGTCAGTCACAAGGGTCGCCTCCAAAAAGGCAGATCTCATCGCCAAC
ATTGACAAGTACTTTCAGCagaagttcaaagagaCTAAGGCCTGATTCGTGTTCCTTACTTT
TTCCTCGCAACGTGTTTTTTTCCCACCACATTGCCTATGTTGTAATGCAATGCAGATG
CTGGCCCAGTTTTTGACGATTCTCGAAAATTGGCATTTTCGTCGATGCCATTGGCCAA
ACTGAAAATTCAAGACAAAATAGATTGGATTTTATCTGCAACGTCTTCCACCTACACAA
CCACTCTACAAACTTCAGACAAACATGTTTATAAAAGCAGCTACTAGATCCAAAATGAC
AAGTTCGTTATTCTCTACTACGTTTGTTGTGGCATTTGGATTGGTGGCTAGCAACAAC
CTCTTGCCATGTCCTGTTGACCACTCTATGAATAACGAGACTCCGCAAGAATTGAAAC
CATTGCAGGCTGAATCTTCTACTAGAAAGTTGAACTCTTCCGCTTAAGTCAAATAAAA
CTACTGACACAGATGATGCACAGAAACAACGGATCACGCTCTTGACTGATTAGTCCC
GTCATTTTGGttctcattttcttcacaGTCACCTATCAATGTATGATCACCTGGAAGGATTTCCCT
ACGAtacttcaaatcttttACTTGATAATATTACTCATTATGGCTCAGGAATGCAGACTGCCTGA
TTCAAGACGCTGCTCTTCTTATTTAACACTTGTACACTAACCCCATGGAAGCCAGGGA
AGGGAATAACCATCTCTCTGGTAATAAATCGGTCTTTATTTATGCATAGAAAAGGAATC
TATTATATTTCGTTCATTTG***GCACTCTGCTAACTGTAGATTAACGGCGCTCGACAACC***
CTTAATTCGAGA

The downstream homologous arm of *KpKu70* was marked gray and the underlined parts were the flanking homologous sequences used for assembly.

Table S6. Guide RNA sequence (5’→3’)

Target locus	Sequence (5’→3’)
KpHis4	ATGGCTCTCTTAACGTTATC
KpADH900	CGTTCTTACCACCGTACACG
II-4	CCTAAATACTACCTAAACAG
II-5	aactttgaaacaaaagaagg
II-6	CCAATATAGGATTGAACTCG
KanMX	TTACTCACCACTGCGATCCC
crtE	GGGAGGTTTGTTCCGTATAG
DNA ligase IV	GGCGGGAAACGTTACGCAGC
KpADH900-2	TGTGAAGAGATTATCGCCCC

Table S7. Plasmid sequence

pGS188 (5’→3’, 9907bp)

ATTTAAATGACCCTTGTGACTGACACTTTGGGAGTCCCTATTCTACTTAGT
CTCATATCGCATGAAACTTTTGATAAATTATTTTCTGATAGGAATTTTTCATC
AGATATTATCATCGCGGCTTACGTAATAACAAAAAAAATTGATGGAGTCTAT
ACTAGGCTAACATAAACTAAGTTATTAATTAAACAAAACAAAACGTACTAGC
ATTACTGTCATATATAAGGGCTCCTAACTAAAACTGTAAAGACTTCCCGTCT
GAGTTAAACTTTTCTTTTCTTCTTTGGATCTCCACCGAGCTGAGAGAGGTC
GATTCTTGTTTCATAGAGCCCCGTAATTGACTGATGAATCAGTGTGGCGTC
CAGGACCTCCTTTGTAGAGGTGTACCGCTTTCTGTCTATGGTGGTGTCGA
AGTACTTGAAGGCTGCAGGCGCGCCCAAGTTGGTCAGAGTAAACAAGTG
GATAATGTTTTCTGCCTGCTCCCTGATGGGCTTATCCCTGTGCTTATTGTA
AGCAGAAAGCACCTTATCGAGGTTAGCGTCGGCGAGGATCACTCTTTTG
GAGAATTCGCTTATTTGCTCGATGATCTCATCAAGGTAGTGTTTGTGTTGT
TCCACGAACAGCTGCTTCTGCTCATTATCTTCGGGGGACCCTTTGAGCTT
TTCATAGTGGCTGGCCAGATACAAGAAATTAACGTATTTAGAGGGCAGTGC
CAGCTCGTTACCTTTCTGCAGCTCGCCCGCACTAGCGAGCATTCGTTTCC
GGCCGTTTTCAAGCTCAAAGAGAGAGTACTTGGGAAGCTTAATGATGAGG
TCTTTTTTGACCTCTTTATATCCTTTCGCCTCGAGAAAGTCGATGGGGTTT
TTTTCGAAGCTTGATCGCTCCATGATTGTGATGCCCAGCAGTTCCTTGAC
GCTTTTGAGTTTTTTAGACTTCCCTTTCTCCACTTTGGCCACAACCAGTAC
ACTGTAAGCGACTGTAGGAGAATCGAATCCGCCGTATTTCTTGGGGTCCC
AATCTTTTTTGCGTGCGATCAGCTTGTCGCTGTTCCTTTTCGGGAGGATA
CTTTCCTTGGAGAAGCCTCCGGTCTGTACTTCGGTCTTTTTAACGATGTTC
ACCTGCGGCATGGACAGGACCTTCCGGACTGTCGCGAAATCCCTACCCT
TGTCCCACACGATTTCTCCTGTTTCTCCGTTTGTTTCGATAAGTGGTCGCT
TCCGAATCTCTCCATTGGCCAGTGTAATCTCGGTCTTGAAAAAATTCATAAT
ATTGCTGTAAAAGAAGTACTTAGCGGTGGCCTTGCCTATTTCCTGCTCAGA
CTTTGCGATCATTTTCCTAACATCGTACACTTTATAGTCTCCGTAAACAAAT
TCAGATTCAAGCTTGGGATATTTTTTGATAAGTGCAGTGCCTACCACTGCA
TTCAGGTAGGCATCATGCGCATGGTGGTAATTGTTGATCTCTCTCACCTTA
TAAAACTGAAAGTCCTTTCTGAAATCTGAAACCAGCTTAGACTTCAGAGTA
ATAACTTTCACCTCTCGAATCAGTTTGTCATTTTCATCGTACTTGGTGTTCA
TGCGTGAATCGAGAATTTGGGCCACGTGCTTGGTGATCTGGCGTGTCTC
AACAAGCTGCCTTTTGATGAAGCCGGCTTTATCCAACTCAGACAGGCCAC
CTCGTTCAGCCTTAGTCAGATTATCGAACTTCCGTTGTGTGATCAGTTTGG
CGTTCAGCAGCTGCCGCCAATAATTTTTCATTTTCTTGACAACTTCTTCTG
AGGGGACGTTATCACTCTTCCCTCTATTTTTATCGGATCTTGTCAACACTTT
ATTATCAATAGAATCATCTTTGAGAAAAGACTGGGGCACGATATGATCCAC
GTCGTAGTCGGAGAGCCGATTGATGTCCAGTTCCTGATCCACGTACATGT
CCCTGCCGTTCTGCAGGTAGTACAGGTAGAGCTTCTCATTCTGAAGCTGG
GTGTTTTCAACTGGGTGTTCCTTAAGGATTTGGGACCCCAGTTCTTTTATA
CCCTCTTCAATCCTCTTCATCCTTTCCCTACTGTTCTTCTGTCCCTTCTGG
GTAGTTTGGTTCTCTCGGGCCATCTCGATAACGATATTCTCGGGCTTATGC
CTTCCCATTACTTTGACGAGTTCATCCACGACCTTAACGGTCTGCAGTATT
CCCTTTTTGATAGCTGGGCTACCTGCAAGATTAGCGATGTGCTCGTGAAG
ACTGTCCCCCTGGCCAGAAACTTGTGCTTTCTGGATGTCCTCCTTAAAGG
TGAGAGAGTCATCATGGATCAACTGCATGAAGTTCCGGTTGGCAAATCCA
TCGGACTTAAGAAAATCCAGGATTGTCTTTCCACTCTGCTTGTCTCGGATC
CCATTGATCAGTTTTCTTGACAGCCGCCCCCATCCTGTATATCGGCGCCT
CTTGAGCTGTTTCATGACTTTGTCGTCGAAGAGATGAGCGTAAGTTTTCA
AGCGTTCTTCAATCATCTCCCTATCTTCAAACAACGTAAGGGTGAGGACAA

TGTCCTCAAGAATGTCCTCGTTCTCCTCATTGTCCAGGAAGTCCTTGTCTT
TAATGATTTTCAGGAGATCGTGATACGTTCCCAGGGATGCGTTGAAGCGA
TCCTCCACTCCGCTGATTTCAACAGAGTCGAAACATTCAATCTTTTTGAAA
TAGTCTTCTTTGAGCTGTTTCACGGTAACTTTCCGGTTCGTCTTGAAGAG
GAGGTCCACGATAGCTTTCTTCTGCTCTCCAGACAGGAATGCTGGCTTTC
TCATCCCTTCTGTGACGTATTTGACCTTGGTGAGCTCGTTATAAACTGTGA
AGTACTCGTACAGCAGAGAGTGTTTAGGAAGCACCTTTTCGTTAGGCAGA
TTTTTATCAAAGTTAGTCATCCTTTCGATGAAGGACTGGGCAGAGGCCCC
CTTATCCACGACTTCCTCGAAGTTCCAGGGAGTGATGGTTTCTTCTGATTT
GCGAGTCATCCACGCGAATCTGGAATTTCCCCGGGCGAGGGGGCCTACA
TAGTAGGGTATCCGAAATGTGAGGATTTTCTCAATCTTTTCCCTGTTATCTT
TCAAAAAGGGGTAGAAATCCTCTTGCCGCCTGAGGATAGCGTGCAGTTC
GCCCAGGTGAATCTGGTGGGGGATGCTTCCATTGTCGAAAGTGCGCTGT
TTGCGCAACAGATCTTCTCTGTTAAGCTTTACCAGCAGCTCCTCGGTGCC
GTCCATTTTTTCCAAGATGGGCTTAATAAATTTGTAAAATTCCTCCTGGCTT
GCTCCGCCGTCAATGTATCCGGCGTAGCCATTTTTAGACTGATCGAAGAA
AATTTCCTTGTACTTCTCAGGCAGTTGCTGTCTGACAAGGGCCTTCAGCA
AAGTCAAGTCTTGGTGGTGCTCATCATAGCGCTTGATCATACTAGCGCTC
AGCGGAGCTTTGGTGATCTCCGTGTTCACTCGCAGAATATCACTCAGCAG
AATGGCGTCTGACAGGTTCTTTGCCGCCAAAAAAAGGTCTGCGTACTGGT
CGCCGATCTGGGCCAGCAGATTGTCGAGATCATCATCGTAGGTGTCTTTG
CTCAGTTGAAGCTTGGCATCTTCGGCCAGGTCGAAGTTAGATTTAAAGTT
GGGGGTCAGCCCGAGTGACAGGGCGATAAGATTACCAAACAGGCCGTTC
TTCTTCTCCCCAGGGAGCTGTGCGATGAGGTTTTCGAGCCGCCGGGATT
TGGACAGCCTAGCGCTCAGGATTGCTTTGGCGTCAACTCCGGATGCGTT
GATCGGGTTCTCTTCGAAAAGCTGATTGTAAGTCTGAACCAGTTGGATAA
AGAGTTTGTCGACATCGCTGTTGTCTGGGTTCAGGTCCCCCTCGATGAG
GAAGTGTCCCCGAAATTTGATCATATGCGCCAGCGCGAGATAGATCAACC
GCAAGTCAGCCTTATCAGTACTGTCTACAAGCTTCTTCCTCAGATGATATAT
GGTTGGGTACTTTTCATGGTACGCCACCTCGTCCACGATATTGCCAAAGA
TTGGGTGGCGCTCGTGCTTTTTATCCTCCTCCACCAAAAAGGACTCCTCC
AGCCTATGGAAGAAAGAGTCATCCACCTTAGCCATCTCATTACTAAAGATC
TCCTGCAGGTAGCAGATCCGATTCTTTCTGCGGGTATATCTGCGCCGTGC
TGTTCTTTTGAGCCGCGTGGCTTCGGCCGTCTCCCCGGAGTCGAACAGG
AGGGCGCCAATGAGGTTCTTCTTTATGCTGTGGCGATCGGTATTGCCCAG
AACTTTGAATTTTTTGCTCGGCACCTTGTACTCGTCCGTAATGACGGCCC
AGCCGACGCTGTTTGTGCCGATATCGAGCCCAATGGAGTACTTCTTGTCC
ATCGTTTCGGAATTCTGTTGTAGTTTTAATATAGTTTGAGTATGAGATGGAA
CTCAGAACGAAGGAATTATCACCAGTTTATATATTCTGAGGAAAGGGTGTG
TCCTAAATTGGACAGTCACGATGGCAATAAACGCTCAGCCAATCAGAATG
CAGGAGCCATAAATTGTTGTATTATTGCTGCAAGATTTATGTGGGTTCACAT
TCCACTGAATGGTTTTCACTGTAGAATTGGTGTCCTAGTTGTTATGTTTCG
AGATGTTTTCAAGAAAAACTAAAATGCACAAACTGACCAATAATGTGCCGT
CGCGCTTGGTACAAACGTCAGGATTGCCACCACTTTTTTCGCACTCTGGT
ACAAAAGTTCGCACTTCCCACTCGTATGTAACGAAAAACAGAGCAGTCTAT
CCAGAACGAGACAAATTAGCGCGTACTGTCCCATTCCATAAGGTATCATAG
GAAACGAGAGTCCTCCCCCCATCACGTATATATAAACACACTGATATCCCA
CATCCGCTTGTCACCAAACTAATACATCCAGTTCAAGTTACCTAAACAAAT
CAAAATTGCACTGATGAGTCCGTGAGGACGAAACGAGTAAGCTCGTCAG
AGACCcaatacgcaaaccgcctctccccgcgcgttggccgattcattaatgcagctggcacgacaggttt

cccgactggaaagcgggcagtgagcgcaacgcaattaatgtgagttagctcactcattaggcaccccagg
ctttacactttatgcttccggctcgtatgttgtgtggaattgtgagcggataacaatttcacacatactagagaaa
gaggagaaatactagatggcttcctccgaagacgttatcaaagagttcatgcgtttcaaagttcgtatggaa
ggttccgttaacggtcacgagttcgaaatcgaaggtgaaggtgaaggtcgtccgtacgaaggtacccaga
ccgctaaactgaaagttaccaaaggtggtccgctgccgttcgcttgggacatcctgtccccgcagttccagta
cggttccaaagcttacgttaaacacccggctgacatcccggactacctgaaactgtccttcccggaaggtttc
aaatgggaacgtgttatgaacttcgaagacggtggtgttgttaccgttacccaggactcctccctgcaagac
ggtgagttcatctacaaagttaaactgcgtggCaccaacttcccgtccgacggtccggttatgcagaaaaa
aaccatgggttgggaagcttccaccgaacgtatgtacccggaagacggtgctctgaaaggtgaaatcaaa
atgcgtctgaaactgaaagacggtggtcactacgacgctgaagttaaaaccacctacatggctaaaaaac
cggttcagctgccgggtgcttacaaaaccgacatcaaactggacatcacctcccacaacgaagactacac
catcgttgaacagtacgaacgtgctgaaggtcgtcactccaccggtgcttaataacgctgatagtgctagtgt
agatcgctactagagccaggcatcaaataaaacgaaaggctcagtcgaaagactgggcctttcgttttatct
gttgtttgtcggtgaacgctctctactagagGGTCTCTGTTTTAGAGCTAGAAATAGCAAG
TTAAAATAAGGCTAGTCCGTTATCAACTTGAAAAAGTGGCACCGAGTCGG
TGCTTTTGGCCGGCATGGTCCCAGCCTCCTCGCTGGCGCCGGCTGGGC
AACATGCTTCGGCATGGCGAATGGGACTCAAGAGGATGTCAGAATGCCAT
TTGCCTGAGAGATGCAGGCTTCATTTTTGATACTTTTTTATTTGTAACCTATA
TAGTATAGGATTTTTTTTGTCATTTTGTTTCTTCTCGTACGAGCTTGCTCCT
GATCAGCCTATCTCGCAGCTGATGAATATCTTGTGGTAGGGGTTTGGGAA
AATCATTCGAGTTTGATGTTTTTCTTGGTATTTCCCACTCCTCTTCAGAGTA
CAGAAGATTAAGTGAGACTCGAGTTTTTGTAGAAATGTCTTGGTGTCCTC
GTCCAATCAGGTAGCCATCTCTGAAATATCTGGCTCCGTTGCAACTCCGA
ACGACCTGCTGGCAACGTAAAATTCTCCGGGGTAAAACTTAAATGTGGAG
TAATGGAACCAGAAACATCTCTTCCCTTCTCTCTCCTTCCACCGCCCGTTA
CCGTCCCTAGGAAATTTTACTCTGCTGGAGAGCTTCTTCTACGGCCCCCT
TGCAGCAATGCTCTTCCCAGCATTACGTTGCGGGTAAAACGGAGGTCGT
GTACCCGACCTAGCAGCCCAGGGATGGAAAAGTCCCGGCCGTCGCTGG
CAATAATAGCGGGCGGACGCATGTCATGAGATTATTGGAAACCACCAGAA
TCGAATATAAAAGGCGAACACCTTTCCCAATTTTGGTTTCTCCTGACCCAA
AGACTTTAAATTTAATTTATTTGTCCCTAtttcaatcaattgaacaaCTATCAAAACAC
AgATGGCTCGCAGTTATGCCGAGAGAGCAAATACTCATCAATCACCTGTG
GCACGACGACTGTTTGCGCTTATGGAACAGAAACAGAGTAACCTATGCGC
ATCAGTCGACGTGAGAACAACTAAAGAATTATTGGAGCTTCTAGATAAATT
GGGCCCATTTATCTGTTTGGCCAAGACTCATATCGACATAATTGATGACTT
CACGTATGATGGAACTATTCTGCCTTTATTGGAactatcaaagaaacacaagTTTT
TAATTTTTGAGGACAGAAAGTTTGCTGATATAGGCAACACTGTCAAGCATC
AATATCAAGGAGGTGTCTACAAGATTGCACAATGGGCAGATATTACAAATG
CTCATGGTGTCATTGGTAGTGGAATTGTAAAGGGTCTAAAGGAGGCAGCC
ACTGAGACAACAGATCAACCAAGGGGACTATTGATGTTGGCTGAACTGTC
GTCAAAGGGATCAATTGCCCATGGTAAGTACACCGAAGAAACTGTAGAAA
TTGCAAAATCAGACAAGGAATTCGTCATTGGGTTTATTGCTCAAAATTCTAT
GGGAGGACAAGATGAAGGGTTCGATTGGATTATTATGACACCAGGTGTTG
GTTTGGATGACACTGGTGATGCTCTAGGCCAACAATATCGAACAGTGAGT
CAAGTATTTTCCACTGGCACTGACATCATAATCGTAGGTCGTGGTTTGTTT
GGCAAGGGCAGAGATCCCTTAAAAGAAGGTGAACGGTATAGAAAAGCTG
GGTGGGAAGCTTACCAAAATATTCTGAGGTAATCAAGAGGATGTCAGAAT
GCCATTTGCCTGAGAGATGCAGGCTTCATTTTTGATACTTTTTTATTTGTAA
CCTATATAGTATAGGATTTTTTTTGTCATTTTGTTTCTTCTCGTACGAGCTTG
CTCCTGATCAGCCTATCTCGCAGCTGATGAATATCTTGTGGTAGGGGTTTG

GGAAAATCATTCGAGTTTGATGTTTTTCTTGGTATTTCCCACTCCTCTTCA
GAGTACAGAAGATTAAGTGAGAACAAGGCCAGCTCCAGCTTTTTCCATAG
GCTCCGCCCCCCTGACGAGCATCACAAAAATCGACGCTCAAGTCAGAGG
TGGCGAAACCCGACAGGACTATAAAGATACCAGGCGTTTCCCCCTGGAA
GCTCCCTCGTGCGCTCTCCTGTTCCGACCCTGCCGCTTACCGGATACCT
GTCCGCCTTTCTCCCTTCGGGAAGCGTGGCGCTTTCTCATAGCTCACGC
TGTAGGTATCTCAGTTCGGTGTAGGTCGTTCGCTCCAAGCTGGGCTGTGT
GCACGAACCCCCCGTTCAGCCCGACCGCTGCGCCTTATCCGGTAACTAT
CGTCTTGAGTCCAACCCGGTAAGACACGACTTATCGCCACTGGCAGCAG
CCACTGGTAACAGGATTAGCAGAGCGAGGTATGTAGGCGGTGCTACAGA
GTTCTTGAAGTGGTGGCCTAACTACGGCTACACTAGAAGAACAGTATTTG
GTATCTGCGCTCTGCTGAAGCCAGTTACCTTCGGAAAAAGAGTTGGTAGC
TCTTGATCCGGCAAACAAACCACCGCTGGTAGCGGTGGTTTTTTTGTTTG
CAAGCAGCAGATTACGCGCAGAAAAAAAGGATCTCAAGCAAATTAAAGCC
TTCGAGCGTCCCAAAACCTTCTCAAGCAAGGTTTTCAGTATAATGTTACAT
GCGTACACGCGTCTGTACAGAAAAAAAAGAAAAATTTGAAATATAAATAAC
GTTCTTAATACTAACATAACTATAAAAAAATAAATAGGGACCTAGACTTCAG
GTTGTCTAACTCCTTCCTTTTCGGTTAGAGCGGATGTGGGGGGAGGGCG
TGAATGTAAGCGTGACATAACTAATTACATGATATCGACAAAGGAAAAGGG
GGACGGATCTCCGAGGCCTGGGACCCGTGGGCCGCCGTCGGACGTGT
CAGTCCTGCTCCTCGGCCACGAAGTGCACGCAGTTGCCGGCCGGGTCG
CGCAGGGCGAACTCCCGCCCCCACGGCTGCTCGCCGATCTCGGTCATG
GCCGGCCCGGAGGCGTCCCGGAAGTTCGTGGACACGACCTCCGACCAC
TCGGCGTACAGCTCGTCCAGGCCGCGCACCCACACCCAGGCCAGGGTG
TTGTCCGGCACCACCTGGTCCTGGACCGCGCTGATGAACAGGGTCACGT
CGTCCCGGACCACACCGGCGAAGTCGTCCTCCACGAAGTCCCGGGAGA
ACCCGAGCCGGTCGGTCCAGAACTCGACCGCTCCGGCGACGTCGCGCG
CGGTGAGCACCGGAACGGCACTGGTCAACTTGGCCATGGTTTAGTTCCT
CACCTTGTCGTATTATACTATGCCGATATACTATGCCGATGATTAATTGTCAA
CACCGCCCCTTAGATTAGATTGCTATGCTTTCTTTCTAATGAACAAGAAGTA
AAAAAAGTTGTAATAGAACAAGAAAAATGAAACTGAAACTTGAGAAATTGA
AGACCGTTTATTAACTTAAATATCAATGGAGGTCACTGAAAGAGAAAAAAA
CTAAAAAAAAAAAATTTCAAGAAAAAGAAACGTGATAAAAATTTTTATTGCCT
TTTTCGACGAAGAAAAAGAAACGAGGCGGTCCCTTTTTTCTTTTCCAAAC
CTTTAGTACGGGTAATTAACGACACCCTAGAGGAAGAAAGAGGGAAAATTT
AGTATGCTGTGCTTGGGTGTTTTGAAGTGGTACGGCGATGCGCGGAGTC
CGAGAAAATCTGGAAGAGTAAAAAAGGAGTAGAAACATTGCGaacgaagcatc
tgtgcttTCGACAATTAATATTTACTTATTTTGGTCAACCCCAAATAGGTTGATTT
CATACTTGGTTCATTCAAAAATAAGTAGTCTTTTGAGATCTTTCAATATTATA
ATAAATATACTATAACAGCCGACTTGTTTCATTTTCGCGAATGTTCCCCCAG
C T T A T C T C G A

Annotation:

the sequence (9-258 bp) is ***DAS1* terminator** (3’→5’);
the sequence (264-287 bp) is ***SV40* (nuclear location signal)** (3’→5’);
the sequence (288-4391 bp) is ***SpCas9*** (3’→5’);
the sequence (4405-4954 bp) is **bidirectional promoter HTX1**;
the sequence (4955-4997 bp) is **Hammerhead ribozyme** (5’→3’);
the sequence (5005-6032 bp) is ***RFP* expression cassette** (5’→3’);
the sequence (6040-6115 bp) is **sgRNA Scaffold** (5’→3’);
the sequence (6116-6187 bp) is **HDV ribozyme** (5’→3’);

the sequence (6188-6434 bp) is ***AOX1* terminator** (5’→3’);
the sequence (6441-6926 bp) is ***GAP* promoter** (5’→3’);
the sequence (6928-7719 bp) is ***KpURA3*** (5’→3’);
the sequence (7720-7966 bp) is ***AOX1* terminator** (5’→3’);
the sequence (7986-8574 bp) is ***Ori*** (3’→5’);
the sequence (8575-8822 bp) is ***CYC1* terminator** (3’→5’);
the sequence (8888-9262 bp) is ***BleoR* (encodingbleomycin Resistance Protein)** (3’→5’);
the sequence (9281-9328 bp) is ***EM7* promoter** (3’→5’);
the sequence (9336-9722 bp) is ***TEF1* promoter** (3’→5’);
the sequence (9744-9907 bp) is ***PARS1*** (3’→5’);

pGS327 (5’→3’, 5838bp)

GCGaacgaagcatctgtgcttTCGACAATTAATATTTACTTATTTTGGTCAACCCCAAATAGGTT
GATTTCATACTTGGTTCATTCAAAAATAAGTAGTCTTTTGAGATCTTTCAATATTATAATA
AATATACTATAACAGCCGACTTGTTTCATTTTCGCGAATGTTCCCCCAGCTTATCTCGA
ATTAAGGGTTGTCGAGCGCTCTAGATCTCACTTAATCTTCTGTACTCTGAAGAGGAGT
GGGAAATACCAAGAAAAACATCAAACTCGAATGATTTTCCCAAACCCCTACCACAAGA
TATTCATCAGCTGCGAGATAGGCTGATCAGGAGCAAGCTCGTACGAGAAGAAACAAA
ATGACAAAAAAAATCCTATACTATATAGGTTACAAATAAAAAAGTATCAAAAATGAAGCC
TGCATCTCTCAGGCAAATGGCATTCTGACATCCTCTTGAGTCCCATTCGCCATGCCG
AAGCATGTTGCCCAGCCGGCGCCAGCGAGGAGGCTGGGACCATGCCGGCCAAAAG
CACCGACTCGGTGCCACTTTTTCAAGTTGATAACGGACTAGCCTTATTTTAACTTGCTA
TTTCTAGCTCTAAAACAGAGACCctctagtagagagcgttcaccgacaaacaacagataaaacgaaaggc
ccagtctttcgactgagcctttcgttttatttgatgcctggctctagtagcgatctacactagcactatcagcgttattaagcaccgg
tggagtgacgaccttcagcacgttcgtactgttcaacgatggtgtagtcttcgttgtgggaggtgatgtccagtttgatgtcggtttt
gtaagcacccggcagctgaaccggtttttagccatgtaggtggttttaacttcagcgtcgtagtgaccaccgtctttcagtttca
gacgcattttgatttcacctttcagagcaccgtcttccgggtacatacgttcggtggaagcttcccaacccatggtttttttctgcat
aaccggaccgtcggacgggaagttggtGccacgcagtttaactttgtagatgaactcaccgtcttgcagggaggagtcctg
ggtaacggtaacaacaccaccgtcttcgaagttcataacacgttcccatttgaaaccttccgggaaggacagtttcaggtagt
ccgggatgtcagccgggtgtttaacgtaagctttggaaccgtactggaactgcggggacaggatgtcccaagcgaacggc
agcggaccacctttggtaactttcagtttagcggtctgggtaccttcgtacggacgaccttcaccttcaccttcgatttcgaactc
gtgaccgttaacggaaccttccatacgaactttgaaacgcatgaactctttgataacgtcttcggaggaagccatctagtatttc
tcctctttctctagtatgtgtgaaattgttatccgctcacaattccacacaacatacgagccggaagcataaagtgtaaagcctg
gggtgcctaatgagtgagctaactcacattaattgcgttgcgctcactgcccgctttccagtcgggaaacctgtcgtgccagct
gcattaatgaatcggccaacgcgcggggagaggcggtttgcgtattgGGTCTCTTGCGCATGCCCGGAGT
CGAACCGGGGGCCCGACGATGGCAACGTCGAATTTTACCGCTAAACTACATGCGCG
TTGGCGAATAACTAAAATGTATGTAGTGAgaataaaagaaaaaaaagaagagtcACAGCGGAG
GTCCTGCTCTCCATTTTATATCTGCTGAAAATCTTGtgtaaaaaaaaatttcgtgaaaaagaaattct
TTAGGGAAATTTCAGCCAATCATGTACGAGTGCGTGTGGGACTGCGAAATTAGTCCT
GAGTCTCTTGATGGCAATTCTGATAGAGAGAAATCGCAAACTTTGAGAGGAAGAACAT
CGCAGAGGATAAGGGACAGTCTACAACACTTATATCCGACAAGGATTGTTTTTACGAC
CATTATGAAGCAAGTTGTAGGTATTTTTCCCTTGCTGTCCTCCAGGGTTGGCTCAGTA
CTCTTTCCCACTAAGGGGACACCTCATGCAGTGCGGCAGATAAAAGAGGCGACAGTT
ATCCATTTCAGAGGCCaacgtGGCCACGGCCgggacGGCCGCGGCCGCTTTTTGTAGA
AATGTCTTGGTGTCCTCGTCCAATCAGGTAGCCATCTCTGAAATATCTGGCTCCGTTG
CAACTCCGAACGACCTGCTGGCAACGTAAAATTCTCCGGGGTAAAACTTAAATGTGG
AGTAATGGAACCAGAAACATCTCTTCCCTTCTCTCTCCTTCCACCGCCCGTTACCGTC
CCTAGGAAATTTTACTCTGCTGGAGAGCTTCTTCTACGGCCCCCTTGCAGCAATGCT
CTTCCCAGCATTACGTTGCGGGTAAAACGGAGGTCGTGTACCCGACCTAGCAGCCC
AGGGATGGAAAAGTCCCGGCCGTCGCTGGCAATAATAGCGGGCGGACGCATGTCAT
GAGATTATTGGAAACCACCAGAATCGAATATAAAAGGCGAACACCTTTCCCAATTTTG
GTTTCTCCTGACCCAAAGACTTTAAATTTAATTTATTTGTCCCTAtttcaatcaattgaacaaCTA
TCAAAACACAgatgAGTAAAGGAGAAGAACTTTTCACTGGAGTTGTCCCAATTCTTGTT
GAATTAGATGGTGATGTTAATGGGCACAAATTTTCTGTCAGTGGAGAGGGTGAAGGT

GATGCTACATACGGAAAGCTTACCCTTAAATTTATTTGCACTACTGGAAAACTACCAGT
TCCATGGCCAACACTTGTCACTACTTTCTCTTATGGTGTTCAATGCTTTTCCCGTTATC
CGGATCATATGAAACGGCATGACTTTTTCAAGAGTGCCATGCCCGAAGGTTATGTACA
GGAACGCACTATATCTTTCAAAGATGACGGGAACTACAAGACGCGTGCTGAAGTCAA
GTTTGAAGGTGATACCCTTGTTAATCGTATCGAGTTAAAAGGTATTGATTTTAAAGAAG
ATGGAAACATTCTCGGACACAAACTCGAGTACAACTATAACTCACACAATGTATACATC
ACGGCAGACAAACAAAAGAATGGAATCAAAGCTAACTTCAAAATTCGCCACAACATTG
AAGATGGCTCAGTTCAACTAGCAGACCATTATCAACAAAATACTCCAATTGGCGATGG
CCCTGTCCTTTTACCAGACAACCATTACCTGTCGACACAATCTGCCCTTTCGAAAGAT
CCCAACGAAAAGCGTGACCACATGGTCCTTCTTGAGTTTGTAACTGCTGCTGGGATT
ACACATGGCATGGATGAACTATACAAAtagGTAGATTTGGCCACTAACGGGTTAGTAGT
TGTGTAAGTCTATTaaatttgatttttgtTTATGGATGATCATCGTAGTGGCTATCTGTTTACCTG
TAGGACATCCTAGGGTGGGATGGTGATGTACACCCCCTCAATCTTCAGATGCAACAC
TATGTGGTAGGTCATTGACATAAGGTTTAGGAAAGACCTGTTTTTTGACCAATAAATGG
AACAGGAAGGAAAGGAGGAACCAGTTTACGAACCCCGTCGCTAGCACAAGGCCAGC
TCCAGCTTTTTCCATAGGCTCCGCCCCCCTGACGAGCATCACAAAAATCGACGCTCA
AGTCAGAGGTGGCGAAACCCGACAGGACTATAAAGATACCAGGCGTTTCCCCCTGG
AAGCTCCCTCGTGCGCTCTCCTGTTCCGACCCTGCCGCTTACCGGATACCTGTCCG
CCTTTCTCCCTTCGGGAAGCGTGGCGCTTTCTCATAGCTCACGCTGTAGGTATCTCA
GTTCGGTGTAGGTCGTTCGCTCCAAGCTGGGCTGTGTGCACGAACCCCCCGTTCAG
CCCGACCGCTGCGCCTTATCCGGTAACTATCGTCTTGAGTCCAACCCGGTAAGACAC
GACTTATCGCCACTGGCAGCAGCCACTGGTAACAGGATTAGCAGAGCGAGGTATGTA
GGCGGTGCTACAGAGTTCTTGAAGTGGTGGCCTAACTACGGCTACACTAGAAGAACA
GTATTTGGTATCTGCGCTCTGCTGAAGCCAGTTACCTTCGGAAAAAGAGTTGGTAGC
TCTTGATCCGGCAAACAAACCACCGCTGGTAGCGGTGGTTTTTTTGTTTGCAAGCAG
CAGATTACGCGCAGAAAAAAAGGATCTCAAGCAAATTAAAGCCTTCGAGCGTCCCAA
AACCTTCTCAAGCAAGGTTTTCAGTATAATGTTACATGCGTACACGCGTCTGTACAGA
AAAAAAAGAAAAATTTGAAATATAAATAACGTTCTTAATACTAACATAACTATAAAAAAAT
AAATAGGGACCTAGACTTCAGGTTGTCTAACTCCTTCCTTTTCGGTTAGAGCGGATGT
GGGGGGAGGGCGTGAATGTAAGCGTGACATAACTAATTACATGATATCGACAAAGGA
AAAGGGGGACGGATCTCCGAGGCCTGGGACCCGTGGGCCGCCGTCGGACGTGTTA
GAAAAACTCATCGAGCATCAAATGAAACTGCAATTTATTCATATCAGGATTATCAATACC
ATATTTTTGAAAAAGCCGTTTCTGTAATGAAGGAGAAAACTCACCGAGGCAGTTCCAT
AGGATGGCAAGATCCTGGTATCGGTCTGCGATTCCGACTCGTCCAACATCAATACAA
CCTATTAATTTCCCCTCGTCAAAAATAAGGTTATCAAGTGAGAAATCACCATGAGTGAC
GACTGAATCCGGTGAGAATGGCAAAAGCTTATGCATTTCTTTCCAGACTTGTTCAACA
GGCCAGCCATTACGCTCGTCATCAAAATCACTCGCATCAACCAAACCGTTATTCATTC
GTGATTGCGCCTGAGCGAGGCGAAATACGCGATCGCTGTTAAAAGGACAATTACAAA
CAGGAATCGAATGCAACCGGCGCAGGAACACTGCCAGCGCATCAACAATATTTTCAC
CTGAATCAGGATATTCTTCTAATACCTGGAATGCTGTTTTGCCGGGGATCGCAGTGGT
GAGTAACCATGCATCATCAGGAGTACGGATAAAATGCTTGATGGTCGGAAGAGGCATA
AATTCCGTCAGCCAGTTTAGTCTGACCATCTCATCTGTAACATCATTGGCAACGCTAC
CTTTGCCATGTTTCAGAAACAACTCTGGCGCATCGGGCTTCCCATACAATCGATAGAT
TGTCGCACCTGATTGCCCGACATTATCGCGAGCCCATTTATACCCATATAAATCAGCAT
CCATGTTGGAATTTAATCGCGGCCTCGAAACGTGAGTCTTTTCCTTACCCATGGTTTA
GTTCCTCACCTTGTCGTATTATACTATGCCGATATACTATGCCGATGATTAATTGTCAAC
ACCGCCCCTTAGATTAGATTGCTATGCTTTCTTTCTAATGAACAAGAAGTAAAAAAAGT
TGTAATAGAACAAGAAAAATGAAACTGAAACTTGAGAAATTGAAGACCGTTTATTAACT
TAAATATCAATGGAGGTCACTGAAAGAGAAAAAAACTAAAAAAAAAAAATTTCAAGAAAA
AGAAACGTGATAAAAATTTTTATTGCCTTTTTCGACGAAGAAAAAGAAACGAGGCGGT
CCCTTTTTTCTTTTCCAAACCTTTAGTACGGGTAATTAACGACACCCTAGAGGAAGAA
AGAGGGAAAATTTAGTATGCTGTGCTTGGGTGTTTTGAAGTGGTACGGCGATGCGCG
GAGTCCGAGAAAATCTGGAAGAGTAAAAAAAGGAGTAGAAACATT

Annotation:

the sequence (22-185 bp) is **PARS1** (3’→5’);
the sequence (211-457 bp) is ***AOX1* terminator** (3’→5’);

the sequence (458-529 bp) is **HDV ribozyme** (3’→5’);
the sequence (530-605 bp) is **sgRNA Scaffold** (3’→5’);
the sequence (613-1640 bp) is ***RFP* expression cassette** (3’→5’);
the sequence (1648-1718 bp) is **tRNA^{Gly}** (3’→5’);
the sequence (1719-2141 bp) is **promoter of *KpTEF1*** (3’→5’);
the sequence (2188-2673 bp) is ***GAP* promoter** (5’→3’);
the sequence (2675-3391 bp) is ***GFP*** (5’→3’);
the sequence (3392-3641 bp) is ***DAS2* terminator** (5’→3’);
the sequence (3667-4255 bp) is ***Ori*** (5’→3’);
the sequence (4256-4503 bp) is ***CYC1* terminator** (3’→5’);
the sequence (4569-5378 bp) is ***kanMX*** (3’→5’);
the sequence (5397-5444 bp) is ***EM7* promoter** (3’→5’);
the sequence (5452-5838) is ***TEF1* promoter** (3’→5’).

pZW1 (5’→3’, 5838bp)

gatgcttcgttCGCAATGTTTCTACTCCTTTTTACTCTTCCAGATTTTCTCGGAC
TCCGCGCATCGCCGTACCACTTCAAAACACCCAAGCACAGCATACTAAAT
TTTCCCTCTTTCTTCCTCTAGGGTGTCGTTAATTACCCGTACTAAAGGTTT
GGAAAAGAAAAAAGGGACCGCCTCGTTTCTTTTTCTTCGTCGAAAAAGGC
AATAAAAATTTTTATCACGTTTCTTTTTCTTGAAATTTTTTTTTTTTAGTTTTTT
TCTCTTTCAGTGACCTCCATTGATATTTAAGTTAATAAACGGTCTTCAATTT
CTCAAGTTTCAGTTTCATTTTTCTTGTTCTATTACAACTTTTTTTACTTCTTG
TTCATTAGAAAGAAAGCATAGCAATCTAATCTAAGGGGCGGTGTTGACAAT
TAATCATCGGCATAGTATATCGGCATAGTATAATACGACAAGGTGAGGAACT
AAACCATGGGTAAGGAAAAGACTCACGTTTCGAGGCCGCGATTAAATTCC
AACATGGATGCTGATTTATATGGGTATAAATGGGCTCGCGATAATGTCGGG
CAATCAGGTGCGACAATCTATCGATTGTATGGGAAGCCCGATGCGCCAGA
GTTGTTTCTGAAACATGGCAAAGGTAGCGTTGCCAATGATGTTACAGATG
AGATGGTCAGACTAAACTGGCTGACGGAATTTATGCCTCTTCCGACCATC
AAGCATTTTATCCGTACTCCTGATGATGCATGGTTACTCACCACTGCGATC
CCCGGCAAAACAGCATTCCAGGTATTAGAAGAATATCCTGATTCAGGTGAA
AATATTGTTGATGCGCTGGCAGTGTTCCTGCGCCGGTTGCATTCGATTCC
TGTTTGTAATTGTCCTTTTAACAGCGATCGCGTATTTCGCCTCGCTCAGGC
GCAATCACGAATGAATAACGGTTTGGTTGATGCGAGTGATTTTGATGACG
AGCGTAATGGCTGGCCTGTTGAACAAGTCTGGAAAGAAATGCATAAGCTT
TTGCCATTCTCACCGGATTCAGTCGTCACTCATGGTGATTTCTCACTTGAT
AACCTTATTTTTGACGAGGGGAAATTAATAGGTTGTATTGATGTTGGACGA
GTCGGAATCGCAGACCGATACCAGGATCTTGCCATCCTATGGAACTGCCT
CGGTGAGTTTTCTCCTTCATTACAGAAACGGCTTTTTCAAAAATATGGTATT
GATAATCCTGATATGAATAAATTGCAGTTTCATTTGATGCTCGATGAGTTTT
TCTAACACGTCCGACGGCGGCCCACGGGTCCCAGGCCTCGGAGATCCG
TCCCCCTTTTCCTTTGTCGATATCATGTAATTAGTTATGTCACGCTTACATT
CACGCCCTCCCCCCACATCCGCTCTAACCGAAAAGGAAGGAGTTAGACA
ACCTGAAGTCTAGGTCCCTATTTATTTTTTTATAGTTATGTTAGTATTAAGAA
CGTTATTTATATTTCAAATTTTTCTTTTTTTTCTGTACAGACGCGTGTACGCA
TGTAACATTATACTGAAAACCTTGCTTGAGAAGGTTTTGGGACGCTCGAA
GGCTTTAATTTGCTTGAGATCCTTTTTTTCTGCGCGTAATCTGCTGCTTGC
AAACAAAAAAACCACCGCTACCAGCGGTGGTTTGTTTGCCGGATCAAGA
GCTACCAACTCTTTTTCCGAAGGTAACTGGCTTCAGCAGAGCGCAGATAC
CAAATACTGTTCTTCTAGTGTAGCCGTAGTTAGGCCACCACTTCAAGAACT

CTGTAGCACCGCCTACATACCTCGCTCTGCTAATCCTGTTACCAGTGGCT
GCTGCCAGTGGCGATAAGTCGTGTCTTACCGGGTTGGACTCAAGACGAT
AGTTACCGGATAAGGCGCAGCGGTCGGGCTGAACGGGGGGTTCGTGCA
CACAGCCCAGCTTGGAGCGAACGACCTACACCGAACTGAGATACCTACA
GCGTGAGCTATGAGAAAGCGCCACGCTTCCCGAAGGGAGAAAGGCGGA
CAGGTATCCGGTAAGCGGCAGGGTCGGAACAGGAGAGCGCACGAGGGA
GCTTCCAGGGGGAAACGCCTGGTATCTTTATAGTCCTGTCGGGTTTCGCC
ACCTCTGACTTGAGCGTCGATTTTTGTGATGCTCGTCAGGGGGGCGGAG
CCTATGGAAAAAGCTGGAGCTGGCCTTGTCCAGATGTAATCACCGCCGCT
GAATGCCTGCACTGGGTAGACCCACAGAAATTCGTTGCAAATGTCGCCAA
TTCTCTAAGGGATCATGGCACCTTGAGTTACTGGCTTTATACAgaaccaatttttc
agaacgAAAGGGCCAATCAGGTTTACAACAAATTCACCTACGGGTCTGACT
ACCTGGGCCCATATTGGGATCCTGGCCGTACACATTTCAGAAATCATTTAA
AGGAGTTGAATCACATCTTACTGGATAGCGAGCTTTTTGACGAAGTGAAAA
TTTCTAATTTTAAACAAGAGGAAGGGGTCAAAAACGGAGATATCTTATACttg
gaaaaagagatgACAATCAgtgatttcatcaattttgtATCTAGTTGGCCTTCTGTGTTTT
CGTGGAAGCAGCAACGAGGAAAGGAGGGTATCCTAGATGATTTTTACAAC
GAACTGAACGACTGCTTTGAGGGGGGTAACATGAAAGTAATATGGAACTC
CGTCCTAGTATTTGCCAGGAGGAAGCAAAGGGTTGTATAGGCTTTAGTAC
TTATAGAGGAAACGGGGTTACGTGCAAGCGCGCATGCCTGAGCTTTGAG
GGGGGGGACTTTCAcatctcttcttctcaCACTTAGCCCTAACACAGAGAATAATA
AAAAGCATTGCAAGATGAGTGTTGTCAGCAAGCAATACGACATCCACGAA
GGCATTATCTTTGTAATTGAATTGACCCCGGAGCTTCACGCGCCGGCTTC
AGAAGGGAAATCTCAGCTCCAGATCATCTTAGAGAATGTCAGTGAGGATT
TTTGTAGAAATGTCTTGGTGTCCTCGTCCAATCAGGTAGCCATCTCTGAAA
TATCTGGCTCCGTTGCAACTCCGAACGACCTGCTGGCAACGTAAAATTCT
CCGGGGTAAAACTTAAATGTGGAGTAATGGAACCAGAAACATCTCTTCCC
TTCTCTCTCCTTCCACCGCCCGTTACCGTCCCTAGGAAATTTTACTCTGCT
GGAGAGCTTCTTCTACGGCCCCCTTGCAGCAATGCTCTTCCCAGCATTAC
GTTGCGGGTAAAACGGAGGTCGTGTACCCGACCTAGCAGCCCAGGGATG
GAAAAGTCCCGGCCGTCGCTGGCAATAATAGCGGGCGGACGCATGTCAT
GAGATTATTGGAAACCACCAGAATCGAATATAAAAGGCGAACACCTTTCCC
AATTTTGGTTTCTCCTGACCCAAAGACTTTAAATTTAATTTATTTGTCCCTAtt
tcaatcaattgaacaaCTATCAAAACACAgatgATGGGAAAAGAACAAGATCAGGAT
AAACCCACAGCTATCATCGTGGGATGTGGTATCGGTGGAATCGCCACTGC
CGCTCGTCTTGCTAAAGAAGGTTTCCAGGTCACGGTGTTCGAGAAGAAC
GACTACTCCGGAGGTCGATGCTCTTTAATCGAGCGAGATGGTTATCGATT
CGATCAGGGGCCCAGTTTGCTGCTCTTGCCAGATCTCTCCAAGCAGACA
TTCGAAGATTTGGGAGAGAAGATGGAAGATTGGGTCGATCTCATCAAGTG
TGAACCCAACTATGTTTGCCACTTCCACGATGAAGAGACTTTCACTCTTTC
AACCGACATGGCGTTGCTCAAGCGGGAAGTCGAGCGTTTTGAAGGCAAA
GATGGATTTGATCGGTTCTTGTCGTTTATCCAAGAAGCCCACAGACATTAC
GAGCTTGCTGTCGTTCACGTCCTGCAGAAGAACTTCCCTGGCTTCGCAG
CATTCTTACGGCTACAGTTCATTGGCCAAATCCTGGCTCTTCACCCCTTCG
AGTCTATCTGGACAAGAGTTTGTCGATATTTCAAGACCGACAGATTACGAA
GAGTCTTCTCGTTTGCAGTGATGTACATGGGTCAAAGCCCATACAGTGCG
CCCGGAACATATTCCTTGCTCCAATACACCGAATTGACCGAGGGCATCTG
GTATCCGAGAGGAGGCTTTTGGCAGGTTCCTAATACTCTTCTTCAGATCAT
CAAGCGCAACAATCCCTCAGCCAAGTTCAATTTCAACGCTCCAGTTTCCC
AGGTTCTTCTCTCTCCTGCCAAGGACCGAGCGACTGGTGTTCGACTTGA

ATCCGGCGAGGAACATCACGCCGATGTTGTGATTGTCAATGCTGACCTCG
TTTACGCCTCCGAGCACTTGATTCCTGACGATGCCAGAAACAAGATTGGC
CAACTGGGTGAAGTCAAGAGAAGTTGGTGGGCTGACTTAGTTGGTGGAA
AGAAGCTCAAGGGAAGTTGCAGTAGTTTGAGCTTCTACTGGAGCATGGA
CCGAATCGTGGACGGTCTGGGCGGACACAATATCTTCTTGGCCGAGGAC
TTCAAGGGATCATTCGACACAATCTTCGAGGAGTTGGGTCTCCCAGCCGA
TCCTTCCTTTTACGTGAACGTTCCCTCGCGAATCGATCCTTCTGCCGCTC
CCGAAGGCAAAGATGCTATCGTCATTCTTGTGCCGTGTGGCCATATCGAC
GCTTCGAACCCTCAAGATTACAACAAGCTTGTTGCTCGGGCAATGAAGTT
TGTGATCCACACGCTTTCCGCCAAGCTTGGACTTCCCGACTTTGAAAAAA
TGATTGTGGCAGAGAAGGTTCACGATGCTCCCTCTTGGGAGAAAGAATTC
AACCTCAAGGACGGAAGCATCTTGGGACTGGCTCACAACTTTATGCAAGT
TCTTGGTTTCAGGCCGAGCACCAGACATCCCAAGTATGACAAGTTGTTCT
TTGTCGGGGCTTCGACTCATCCCGGAACTGGGGTTCCCATCGTCTTGGC
TGGAGCCAAGTTAACTGCCAACCAAGTTCTCGAATCCTTTGACCGATCCC
CAGCTCCAGATCCCAATATGTCACTCTCCGTACCATATGGAAAACCTCTCA
AATCAAATGGAACGGGTATCGATTCTCAGGTCCAGCTGAAGTTCATGGAT
TTGGAGAGATGGGTATACCTTTTGGTATTGTTGATTGGGGCCGTGATCGC
TCGATCCGTTGGTGTTCTTGCTTTCTGAtagcGTAGATTTGGCCACTAACGG
GTTAGTAGTTGTGTAAGTCTATTaaatttgatttttgtTTATGGATGATCATCGTAGT
GGCTATCTGTTTACCTGTAGGACATCCTAGGGTGGGATGGTGATGTACAC
CCCCTCAATCTTCAGATGCAACACTATGTGGTAGGTCATTGACATAAGGTT
TAGGAAAGACCTGTTTTTTGACCAATAAATGGAACAGGAAGGAAAGGAGG
AACCAGTTTACGAACCCCGTCTGCTCAAACGAGTGGAGAGGGAAATCGA
TTCAGCAGTTAAATCAATGCTGGAAAATATTCGAGATTACCTAATCGGATCT
GGAACTTACTTCGACCTGACATTTTCTTGCCTGGGGAGCCACGATCGATT
ATGTAATCAAGAATATGGACAGAGGGAAACAGATTTAGCTGTCAAAAGCCC
AAGAGAAGCTACCGATCAATGGATGCGGATAGATAAAGAAAAgtcctttttttttcatt
aGCCATCCGAGTTGTCCAATCAAATGTCTGCCTGCTACGCTGGAGAGGAA
TCACGCGTGTTTAACATTCGGATTGTCGCCTAAAATAAGCCTATTACCTAC
ACAGTAAAACCCGGGGGGTGCTTTGGTATCAATGACCCCGGGATTTTATC
CACcagtttttttctttctggCAAGAGTGCATTGCATCCCCGTACAAATAGTAGCAAC
CTCCACAAGAGGAATCCCCTATGAGCGAGAAGTCCATAGTAATACCCCCG
CGGAAAAGAGATATTTTGTTTCCGTGTTGCCCTTGAACTTCAGTTTCCCCC
ATCAGTTTATATAGTAGCCGGGTTCCCAATCTCTAGCCCTTCTTTCCTCCTA
TTTCATTCCTCTCTTCTTACGTTATCTTACATTAGCgatgATGACGGCTCTCG
CATATTACCAGATCCATCTGATCTATACTCTCCCAATTCTTGGTCTTCTCGG
TCTGCTCACTTCCCCGATTTTGACAAAATTTGACATCTACAAAATATCGATC
CTCGTATTTATTGCGTTTAGTGCAACCACACCATGGGACTCATGGATCATC
AGAAATGGCGCATGGACATATCCATCAGCGGAGAGTGGCCAAGGCGTGT
TTGGAACGTTTCTAGATGTTCCATATGAAGAGTACGCTTTCTTTGTCATTCA
AACCGTAATCACCGGCTTGGTCTACGTCTTGGCAACTAGGCACCTTCTCC
CATCTCTCGCGCTTCCCAAGACTAGATCGTCCGCCCTTTCTCTCGCGCTC
AAGGCGCTCATCCCTCTGCCCATTATCTACCTATTTACCGCTCACCCCAGC
CCATCGCCCGACCCGCTCGTGACAGATCACTACTTCTACATGCGGGCACT
CTCCTTACTCATCACCCCACCTACCATGCTCTTGGCAGCATTATCAGGCG
AATATGCTTTCGATTGGAAAAGTGGCCGAGCAAAGTCAACTATTGCAGCA
ATCATGATCCCGACGGTGTATCTGATTTGGGTAGATTATGTTGCTGTCGGT
CAAGACTCTTGGTCGATCAACGATGAGAAGATTGTAGGGTGGAGGCTTG
GAGGTGTACTACCCATTGAGGAAGCTATGTTCTTCTTACTGACGAATCTAA

TGATTGTTCTGGGTCTGTCTGCCTGCGATCATACTCAGGCCCTATACCTG
CTACACGGTCGAACTATTTATGGCAACAAAAAGATGCCATCTTCATTTCCC
CTCATTACACCGCCTGTGCTCTCCCTGTTTTTTAGCAGCCGACCATACTCT
TCTCAGCCAAAACGTGACTTGGAACTGGCAGTCAAGTTGTTGGAGGAAA
AGAGCCGGAGCTTTTTTGTTGCCTCGGCTGGATTTCCTAGCGAAGTTAGG
GAGAGGCTGGTTGGACTATACGCATTCTGCCGGGTGACTGATGATCTTAT
CGACTCTCCTGAAGTATCTTCCAACCCGCATGCCACAATTGACATGGTCT
CCGATTTTCTTACCCTACTATTTGGGCCCCCGCTACACCCTTCGCAACCT
GACAAGATCCTTTCTTCGCCTTTACTTCCTCCTTCGCACCCTTCCCGACC
CACGGGAATGTATCCCCTCCCGCCTCCTCCTTCGCTCTCGCCTGCCGAG
CTCGTTCAATTCCTTACCGAAAGGGTTCCCGTTCAATACCATTTCGCCTTC
AGGTTGCTCGCTAAGTTGCAAGGGCTGATCCCTCGATACCCACTCGACG
AACTCCTTAGAGGATACACCACTGATCTTATCTTTCCTTTATCGACAGAGG
CAGTCCAGGCTCGGAAGACGCCTATCGAGACCACAGCTGACTTGCTGGA
CTATGGTCTATGTGTAGCAGGCTCAGTCGCCGAGCTATTGGTCTATGTCTC
TTGGGCAAGTGCACCAAGTCAGGTCCCTGCCACCATAGAAGAAAGAGAA
GCTGTGTTAGTGGCAAGCCGAGAGATGGGAACTGCCCTTCAGTTGGTGA
ACATTGCTAGGGACATTAAAGGGGACGCAACAGAAGGGAGATTTTACCTA
CCACTCTCATTCTTTGGTCTTCGGGATGAATCAAAGCTTGCGATCCCGAC
TGATTGGACGGAACCTCGGCCTCAAGATTTCGACAAACTCCTCAGTCTAT
CTCCTTCGTCCACATTACCATCTTCAAACGCCTCAGAAAGCTTCCGGTTC
GAATGGAAGACGTACTCGCTTCCATTAGTCGCCTACGCAGAGGATCTTGC
CAAACATTCTTATAAGGGAATTGACCGACTTCCTACCGAGGTTCAAGCGG
GAATGCGAGCGGCTTGCGCGAGCTACCTACTGATCGGCCGAGAGATCAA
AGTCGTTTGGAAAGGAGACGTCGGAGAGAGAAGGACAGTTGCCGGATG
GAGGAGAGTACGGAAAGTCTTGAGTGTGGTCATGAGCGGATGGGAAGG
GCAGTAAtagcGTAGTAGTAGTAGTAGCAGAGTATCTACAGTGGTGTGTATA
ATGTATATGAGTGTACTTACCAACCAAATTCCGTTTAGTATTTCGTCGACGA
TGATGTAGTACGAGTCCTTCGCGAATCCGTTACTCTCAAGACGGGGAAAA
AAAACGACGAAAATGACCAACTTACTCAACTAAGCAAACCTCAAGAAACAT
AACACTTTGTTGTGAGACAGTAATAAAAAGCTCACAGCGTACACATCACCA
CTCATTTCTCTATTGAACGGcttgaaatttggaaaccagatgaaaaataaaaggaatggaag
aagaatgAGAAAAGGATAATTAATCTTTGGTTTAGCTAAATTCTTCATTGCACT
TTGACCTTAAAGGGGCTGATTTAAGGTTATGCCggggaagaagaaatagcGCGA
TGAGCAAAGTCGATGCCTAAAGGAGTGGTTTTGCTACCTCATTTAAGAAG
AGAATAGGACGTGCATCCAGCGATGCGTGCTAGGACAAAGAACCGCACT
TGGCGGGTACAAACCTGACGTCATTTCCTGATATTATTGACATTTGAGCTG
ACCAATTAAGGTGCCCATCCACAATAGCCACCTGGATAGCGGAATGCACC
CCCATTGAGTTGATCAAACTACCATTTTGCTTATACCTCAAGTTAATGTTGA
ACTACCATTCTTCACATGCTCCTCCTAGATCCCCTGTCCCCTTTCTCCCCC
TCTTTCATCCTTTAATTTGCATTTCTTGACGGTCTTCTATCCCTAGAAAGTT
TGGAACGCCTGCTATATGGTTAGGACACGACTGACTAGCTAtaaaattttcagac
cagactctttctcttcttaaCGCAAATTTAACAGGCAGACAACAACATAGGAAAGAAT
CACCATATAGGTTGGACTCTTTACAGACGTCCTTGGCCGTTGACCATGGT
GGTACAGTTGTCCAAGTTCTACAAGTTTGTCTGAAGAATGAAGTTATTGGT
CTTGGGTGCAGCTTTCCATCTGTTCGATTTATTCGGCTAAGAGTTTACCAT
TGTGTGCTCGTATGGGGAAGGGTGCAAGGATCAGTAATACAGTCGAACCT
GGAGTATCTACCATAGTGGGGATACAATGTAGTTTATCTGTTATCTCGATTG
TTCCTAATTAAggttttctttgatccTCTTCTAGTCCACACCTCCTAGATGACATTCG
AGCTGCCTGGATTGGATGCCTAGGTTTATTGCCTAGTTCAATACAATTCGT

GCGGGCTACAGTAGAAGGCCCTTACATAATCCGGAAAGCATGGTCCCCC
ACCAAATTGAGAGCTTTTTCAGCCTTCACTGGTGGTATCATTTTCGGGAGA
TAATAAGGTTTCGATTGGGAATTCCCACCAGAGAACACTATAGAGGGACC
AAGCTGATGCTAGCCTGACATCCCCAAAGCACACTTCGTAATTGAAAACC
GTTACCTCTAGCACACTGTCCAGACTACCCCCGTCAAAAAAACGCTCTTT
TTCTCGACTAATTGAGTCTTCAACTCATCCCGTCCTTGCCGAATTACTTGA
ATTCATTTCACACCTCCGTTGCTTACGTACTCTCACCGGTCTCCGGTGTAC
ATGGATCCGCTATTGCCAGATATTTCTCATACAACAATCACCAGATCAAGG
TCGTGAACGGACCAATGGCATCCAGAGCAATCCTGAACAGATAGGGGTC
CGGGCTGTATAAAGTGAAATAACGTGACTTGAACCAGCAACTATGTCCCA
GTTGTGCTACACTTAACACGCGATTACCCCGGAGCTCACCAGGCCTCTTC
CCCCTCTCATTGGAACCCTCCTAGCGCTTCGAAATAATGGCTGCGTACTAT
TTAACTGGTGCCAGTTCCCGCTGACAATatcctttttcttctcccCTTAGTTCCCCAC
ATATCAATTGAACATATTTTTTACACAgATGGACCAATTGGTGAAAACTGAA
GTCACCAAGAAGTCTTTTACTGCTCCTGTACAAAAGGCTTCTACACCAGTT
TTAACCAATAAAACAGTCATTTCTGGATCGAAAGTCAAAAGTTTATCATCTG
CGCAATCGAGCTCATCAGGACCTTCATCATCTAGTGAGGAAGATGATTCC
CGCGATATTGAAAGCTTGGATAAGAAAATACGTCCTTTAGAAGAATTAGAA
GCATTATTAAGTAGTGGAAATACAAAACAATTGAAGAACAAAGAGGTCGCT
GCCTTGGTTATTCACGGTAAGTTACCTTTGTACGCTTTGGAGAAAAAATTA
GGTGATACTACGAGAGCGGTTGCGGTACGTAGGAAGGCTCTTTCAATTTT
GGCAGAAGCTCCTGTATTAGCATCTGATCGTTTACCATATAAAAATTATGAC
TACGACCGCGTATTTGGCGCTTGTTGTGAAAATGTTATAGGTTACATGCCT
TTGCCCGTTGGTGTTATAGGCCCCTTGGTTATCGATGGTACATCTTATCATA
TACCAATGGCAACTACAGAGGGTTGTTTGGTAGCTTCTGCCATGCGTGGC
TGTAAGGCAATCAATGCTGGCGGTGGTGCAACAACTGTTTTAACTAAGGA
TGGTATGACAAGAGGCCCAGTAGTCCGTTTCCCAACTTTGAAAAGATCTG
GTGCCTGTAAGATATGGTTAGACTCAGAAGAGGGACAAAACGCAATTAAA
AAAGCTTTTAACTCTACATCAAGATTTGCACGTCTGCAACATATTCAAACTT
GTCTAGCAGGAGATTTACTCTTCATGAGATTTAGAACAACTACTGGTGACG
CAATGGGTATGAATATGATTTCTAAAGGTGTCGAATACTCATTAAAGCAAAT
GGTAGAAGAGTATGGCTGGGAAGATATGGAGGTTGTCTCCGTTTCTGGTA
ACTACTGTACCGACAAAAAACCAGCTGCCATCAACTGGATCGAAGGTCGT
GGTAAGAGTGTCGTCGCAGAAGCTACTATTCCTGGTGATGTTGTCAGAAA
AGTGTTAAAAAGTGATGTTTCCGCATTGGTTGAGTTGAACATTGCTAAGAA
TTTGGTTGGATCTGCAATGGCTGGGTCTGTTGGTGGATTTAACGCACATG
CAGCTAATTTAGTGACAGCTGTTTTCTTGGCATTAGGACAAGATCCTGCAC
AAAATGTTGAAAGTTCCAACTGTATAACATTGATGAAAGAAGTGGACGGTG
ATTTGAGAATTTCCGTATCCATGCCATCCATCGAAGTAGGTACCATCGGTG
GTGGTACTGTTCTAGAACCACAAGGTGCCATGTTGGACTTATTAGGTGTAA
GAGGCCCGCATGCTACCGCTCCTGGTACCAACGCACGTCAATTAGCAAG
AATAGTTGCCTGTGCCGTCTTGGCAGGTGAATTATCCTTATGTGCTGCCCT
AGCAGCCGGCCATTTGGTTCAAAGTCATATGACCCACAACAGGAAACCTG
CTGAACCAACAAAACCTAACAATTTGGACGCCACTGATATAAATCGTTTGA
AAGATGGGTCCGTCACCTGCATTAAATCCTAAtagcTCAAGAGGATGTCAGA
ATGCCATTTGCCTGAGAGATGCAGGCTTCATTTTTGATTACTTTTTTATTTG
TAACCTATATAGTATAGGatttttttgtcattttgtttcttctcGTACGAGCTTGCTCCTGAT
CAGCCTATCTCGCAGCTGATGAATATCTTGTGGTAGGGGTTTGGGAAAAT
CATTCGAGtttgatgtttttcttgGTATTTCCCACTCCTCTTCAGAGTACAGAAGATT
AAGTGAAACGCAGCGTTTTCTGACGGTACTAGAGGACTCTTAGGGGAAG

GTAGAATCAATAAAGATCATATTAGGTAAGCAAATTTTGGATGGAATAGGAG
ACTAGGTGTGGATGCGCGATCTCGCCAAATTGCACGACCAGAGTGGATG
CCGGATGGTGGTAAACCgtttcttcctttttaCCACCCAAGTGCGAGTGAAACACC
CCATGGCTGCTCTCCGATTGCCCCTCTACAGGCATAAGGGTGTGACTTTG
TGGGCTTGAATTTTACACCCCCTCCAACTTTTCTCGCATCAATTGATCCTG
TTACCAATATTGCATGCCCGGAGGAGACTTGCCCCCTAATTTCGCGGCGT
CGTCCCGGATCGCAGGGTGAGACTGTAGAGACCCCACATAGTGAcaatgatt
atgtaagaAGAGGGGGGTGATTCGGCCGGCTATCGAACTCTAACAACTAGG
GGGGTGAACAATGCCCAGCAGTCCTCCCCACTCTTTGACAAATCAGTATC
ACCGATTAACACCCCAAATCTTATTCTCAACGGTCCCTCATCCTTGCACCC
CTCTTTGGACAAATGGCAGTTAGCATTGGTGCACTGACTGACTGCCCAAC
CTTAAACCCAAATTTCTTAGAAGGGGCCCATCTAGTTAGCGAGGGGTGAA
AAATTCCTCCATCGGAGATGTATTGACCGTAAGTTGCTGCTTAAAAAAAT
CAGTTCAGATAGCGagactttttttgatttcgCAACGGGAGTGCCTGTTCCATTCGAT
TGCAATTCTCACCCCTTCTGCCCAGTCCTGCCAATTGCCCATGAATCTGC
TAATTTCGTTGATTCCCACCCCCCTTTCCAACTCCACAAATTGTCCAATCT
CGTTTTCCATTTGGGAGAATCTGCATGTCGACTACATAAAGCGACCGGTG
TCCGAAAAGATCTGTGTagttttcaacattttgtGCTCCCCCCGCTGTTTGAAAACG
GGGGTGAGCGCTCTCCGGGGTGCGAATTCGTGCCCAATTCCTTTCACCC
TGCCTATTGTAGACGTCAACCCGCATCTGGTGCGAATATAGCGCACCCCC
AATGATCACACCAACAATTGGTCCACCCCTCCCCAATCTCTAATATTCACA
ATTCACCTCACTATAAATACCCCTGTCCTGCTCccaaattctttttttccttcttccaTCAG
CTACTAGCTTTTATCTTATTTACTTTACGAAAgATGGATTACGCGAACATCCT
CACAGCAATTCCACTCGAGTTTACTCCTCAGGATGATATCGTGCTCCTTGA
ACCGTATCACTACCTAGGAAAGAACCCTGGAAAAGAAATTCGATCACAAC
TCATCGAGGCTTTCAACTATTGGTTGGATGTCAAGAAGGAGGATCTCGAG
GTCATCCAGAACGTTGTTGGCATGCTACATACCGCTAGCTTATTAATGGAC
GATGTGGAGGATTCATCGGTCCTCAGGCGTGGGTCGCCTGTGGCCCATC
TAATTTACGGGATTCCGCAGACAATAAACACTGCAAACTACGTCTACTTTC
TGGCTTATCAAGAGATCTTCAAGCTTCGCCCAACACCGATACCCATGCCT
GTAATTCCTCCTTCATCTGCTTCGCTTCAATCATCCGTCTCCTCTGCATCC
TCCTCCTCCTCGGCCTCGTCTGAAAACGGGGGCACGTCAACTCCTAATT
CGCAGATTCCGTTCTCGAAAGATACGTATCTTGATAAAGTGATCACAGACG
AGATGCTTTCCCTCCATAGAGGGCAAGGCCTGGAGCTATTCTGGAGAGAT
AGTCTGACGTGTCCTAGCGAAGAGGAATATGTGAAAATGGTTCTTGGAAA
GACGGGAGGTTTGTTCCGTATAGCGGTCAGATTGATGATGGCAAAGTCAG
AATGTGACATAGACTTTGTCCAGCTTGTCAACTTGATCTCAATATACTTCCA
GATCAGGGATGACTATATGAACCTTCAGTCTTCTGAGTATGCCCATAATAA
GAATTTTGCAGAGGACCTCACAGAAGGAAAATTCAGTTTTCCCACTATCC
ACTCGATTCATGCCAACCCCTCATCGAGACTCGTCATCAATACGTTGCAG
AAGAAATCGACCTCTCCTGAGATCCTTCACCACTGTGTAAACTACATGCG
CACAGAAACCCACTCATTCGAATATACTCAGGAAGTCCTCAACACCTTGTC
AGGTGCACTCGAGAGAGAACTAGGAAGGCTTCAAGGAGAGTTCGCAGAA
GCTAACTCAAAGATTGATCTTGGAGACGTAGAGTCGGAAGGAAGAACGG
GGAAGAACGTCAAATTGGAAGCGATCCTGAAAAAGCTAGCCGATATCCCT
CTGTagcACGGGAAGTCTTTACAGTTTTAGTTAGGAGCCCTTATATATGACA
GTAATGCTAGTACGTTTTGTTTTGTTTAATTAATAACTTAGTTTATGTTAGCC
TAGTATAGACTCCATCAATTTTTTTTTGTTATTACGTAAGCCGCGATGATAATA
TCTGATGAAAAATTCCTATCAGAAAATAATTTATCAAAAGTTTCATGCGATAT
GAGACTAAGTAGAATAGGGACTCCCAAAGTGTCAGTCACAAGGGTCGCC

TCCAAAAAGGCAGATCTCATCGCCAACATTGACAAGTACTTTCAGCagaagt
tcaaagagaCTAAGGCCTGATTCGTGTTCCTTACTTTTTCCTCGCAACGTGTT
TTTTTCCCACCACATTGCCTATGTTGTAATGCAATGCAGATGCTGGCCCAG
TTTTTGACGATTCTCGAAAATTGGCATTTTCGTCGATGCCATTGGCCAAAC
TGAAAATTCAAGACAAAATAGATTGGATTTTATCTGCAACGTCTTCCACCTA
CACAACCACTCTACAAACTTCAGACAAACATGTTTATAAAAGCAGCTACTA
GATCCAAAATGACAAGTTCGTTATTCTCTACTACGTTTGTTGTGGCATTTG
GATTGGTGGCTAGCAACAACCTCTTGCCATGTCCTGTTGACCACTCTATG
AATAACGAGACTCCGCAAGAATTGAAACCATTGCAGGCTGAATCTTCTACT
AGAAAGTTGAACTCTTCCGCTTAAGTCAAATAAAACTACTGACACAGATGA
TGCACAGAAACAACGGATCACGCTCTTGACTGATTAGTCCCGTCATTTTG
GttctcattttcttcacaGTCACCTATCAATGTATGATCACCTGGAAGGATTTCCCTA
CGAtacttcaaatcttttACTTGATAATATTACTCATTATGGCTCAGGAATGCAGACT
GCCTGATTCAAGACGCTGCTCTTCTTATTTAACACTTGTACACTAACCCCA
TGGAAGCCAGGGAAGGGAATAACCATCTCTCTGGTAATAAATCGGTCTTTA
TTTATGCATAGAAAAGGAATCTATTATATTTCGTTCATTTGGCACTCTGCTAA
CTGTAGATTAACGGCGCTCGACAACCCTTAATTCGAGATAAGCTGGGGGA
ACATTCGCGAAAATGAAACAAGTCGGCTGTTATAGTATATTTATTATAATATT
GAAAGATCTCAAAAGACTACTTATTTTTGAATGAACCAAGTATGAAATCAAC
CTATTTGGGGTTGACCAAAATAAGTAAATATTAATTGTCGAaagcaca

Annotation:

the sequence (15-401 bp) is ***TEF1* promoter** (5’→3’);
the sequence (409-456 bp) is ***EM7* promoter** (5’→3’);
the sequence (475-1284 bp) is ***kanMX*** (5’→3’);
the sequence (1350-1597 bp) is ***CYC1* terminator** (5’→3’);
the sequence (1598-2186 bp) is ***Ori*** (5’→3’);
the sequence (2206-3001bp) is ***Ku70* UHA (the upstream homology arm of Ku70)** (5’→3’);
the sequence (3003-3488 bp) is ***GAP* promoter** (5’→3’);
the sequence (3493-5241 bp) is ***CrtI*** (5’→3’);
the sequence (5246-5495 bp) is ***DAS2* terminator** (5’→3’);
the sequence (5496-6133 bp) is ***FBP1* promoter** (5’→3’);
the sequence (6138-8159 bp) is ***CrtYB*** (5’→3’);
the sequence (8164-8413 bp) is ***FBA1* terminator** (5’→3’);
the sequence (8414-10098 bp) is ***FBA1* promoter** (5’→3’);
the sequence (10100-11677 bp) is ***tHMG1*** (5’→3’);
the sequence (11682-11929 bp) is ***AOX1* terminator** (5’→3’);
the sequence (11930-13141 bp) is ***ADH2* promoter** (5’→3’);
the sequence (13143-14271 bp) is ***CrtE*** (5’→3’);
the sequence (14275-14524 bp) is ***DAS1* terminator** (5’→3’);
the sequence (14525-15374bp) is ***Ku70* DHA (the downstream homology arm of Ku70)** (5’→3’);
the sequence (15394-15557 bp) is ***PARS1*** (3’→5’).

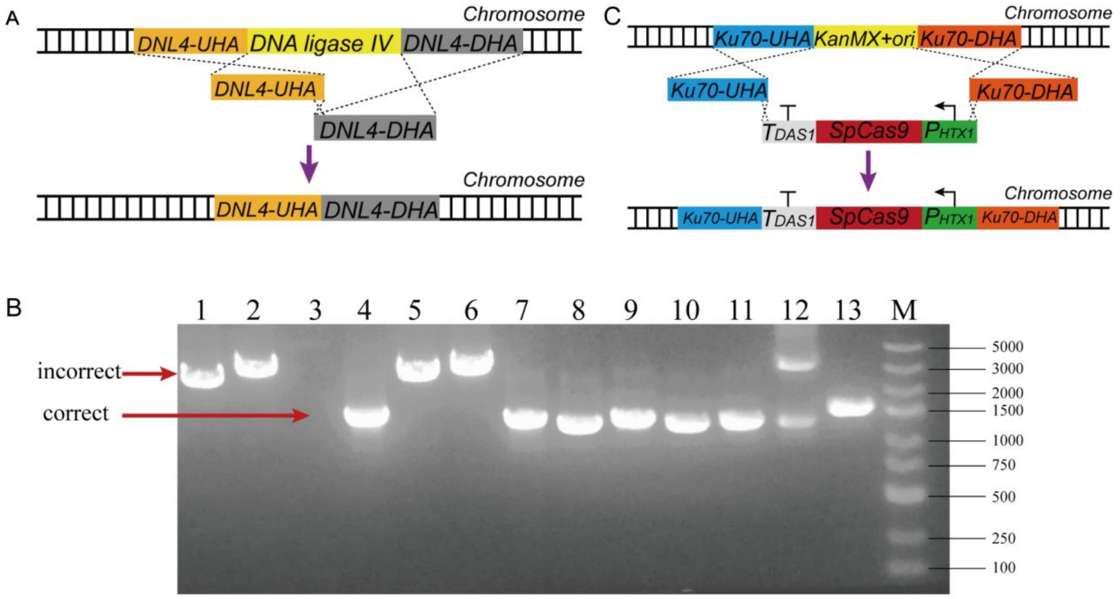

Figure S1. Knockout of *DNA ligase IV* and integration of *SpCas9*. (A) was the design of knockout *DNA ligase IV*, and (B) was a picture of agarose gel electrophoresis validation for the knockout *DNA ligase IV*. (C) The design of the *SpCas9* expression cassette integrated at the *Ku70* locus.

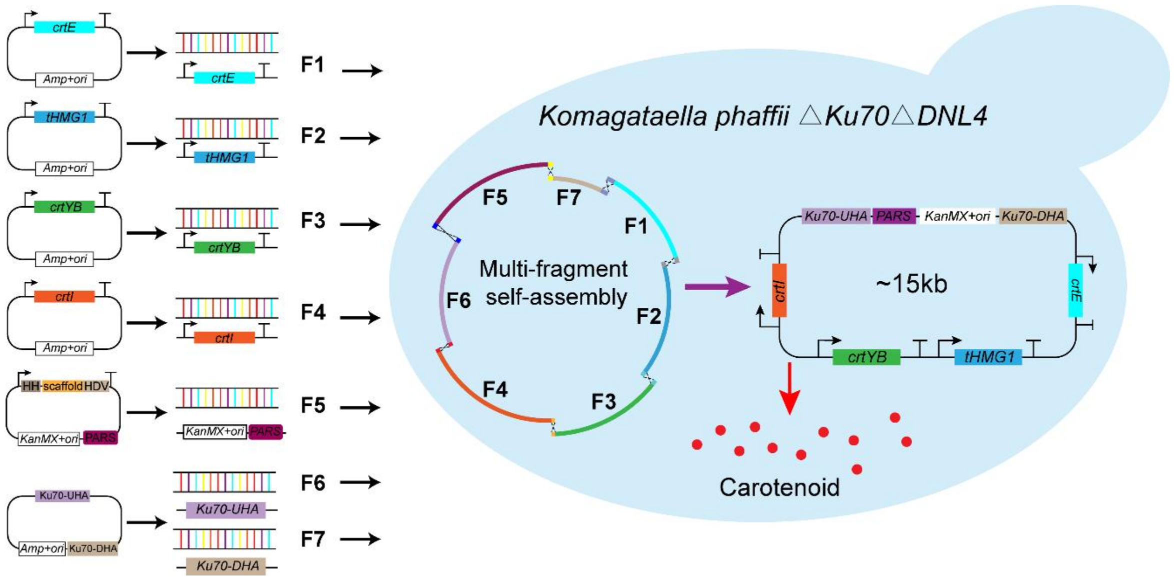

Figure S2. Design of multiple fragments spontaneously assembled intracellularly into a complete plasmid.

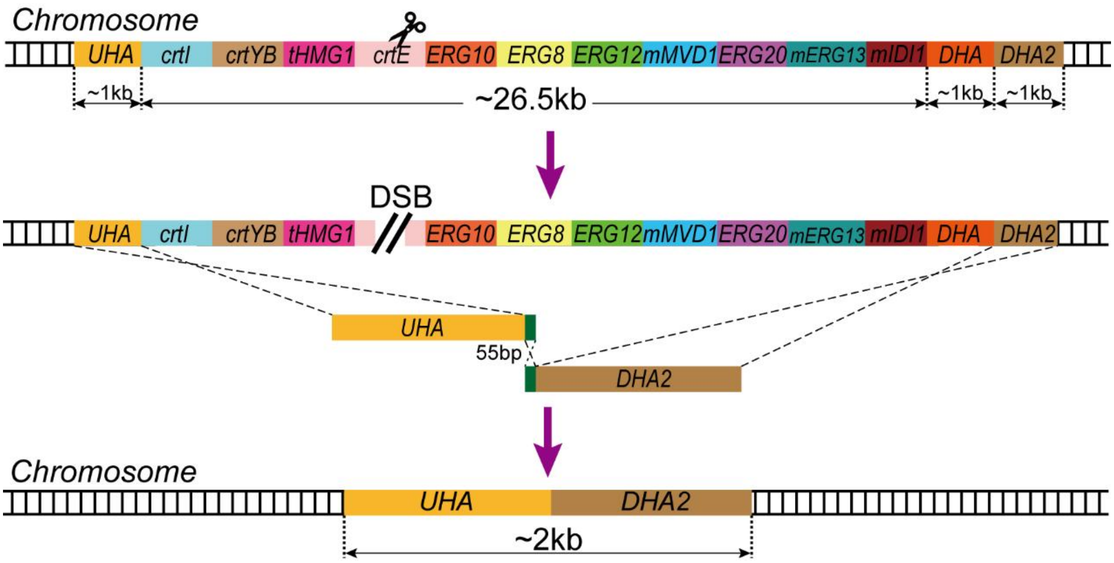

Figure S3. Design of single gRNA plasmid-mediated knockout of a large DNA fragment.The gRNA targeting crtE was designed to cleave the chromosome to form a single DSB, and simultaneously the upstream and downstream homologous arms around 1000bp were provided as repair templates for the knockout of the large DNA fragment.

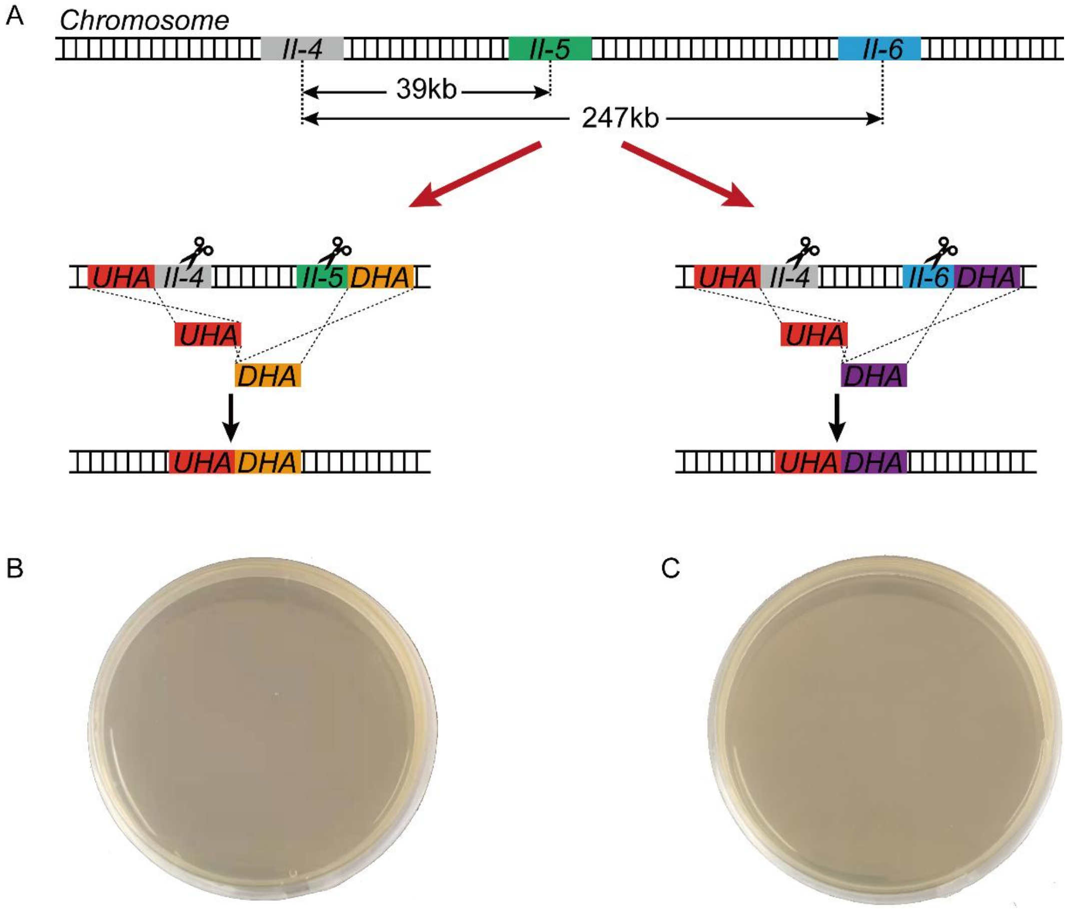

Figure S4. Design and results of double gRNA plasmid-mediated knockout of

larger DNA fragments. (a) Design of double gRNA plasmid-mediated knockout of larger DNA fragments. (b) and (c) were the experiment results of (a), and no colonies grew on the plates.

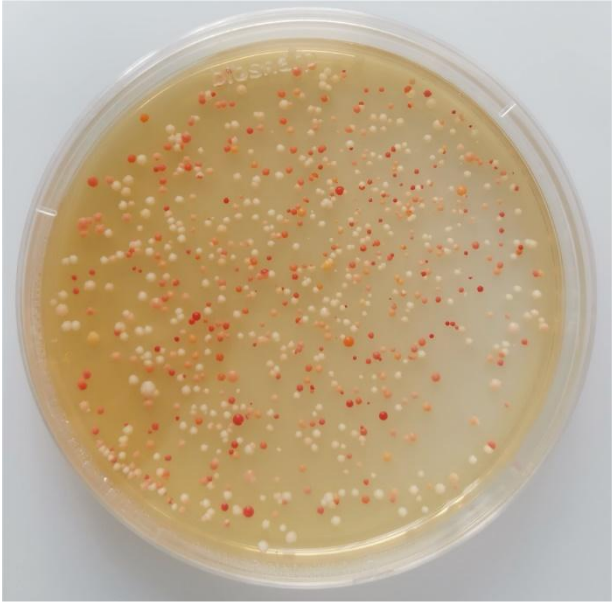

Figure S5. Plate image of yeast combinatorial library. Obviously, it could be observed that colonies of different color grades appeared on the plate.

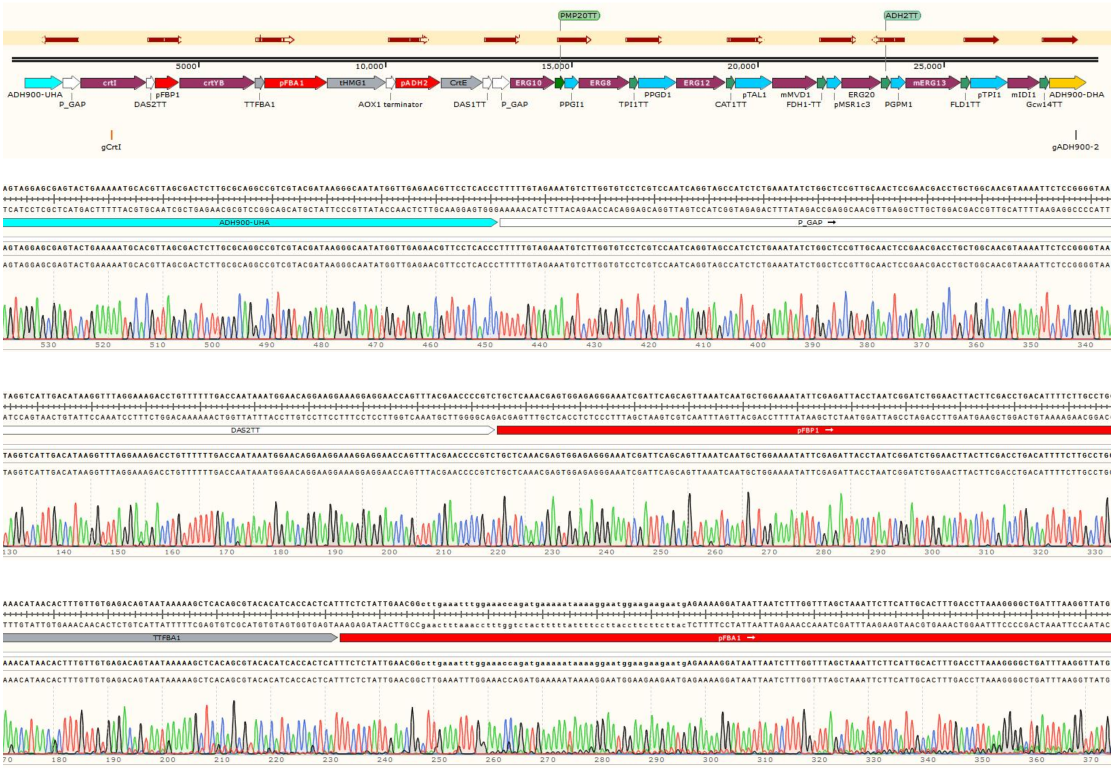

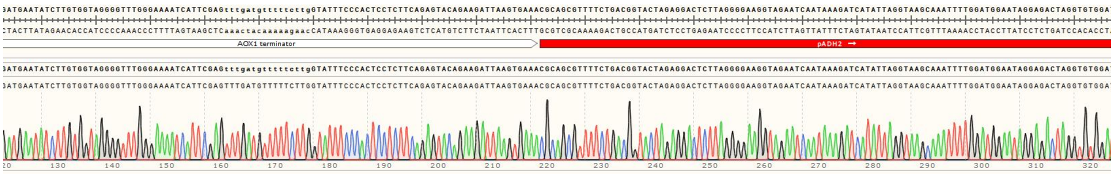

66

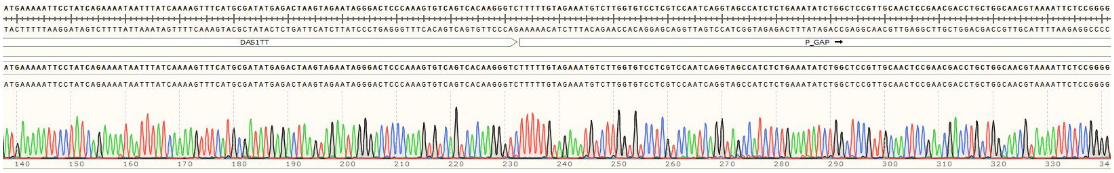

67

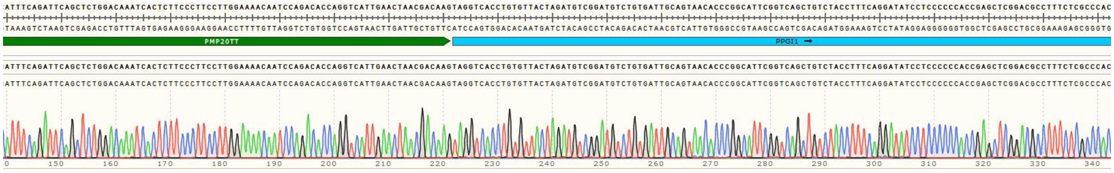

68

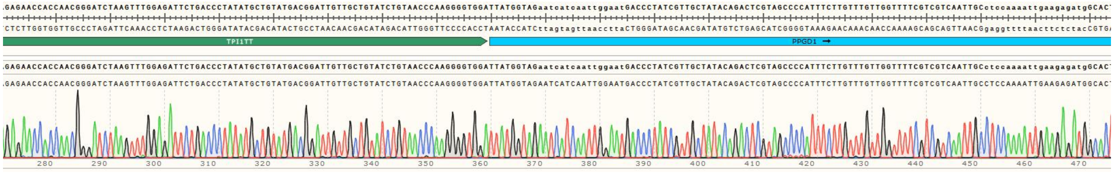

69

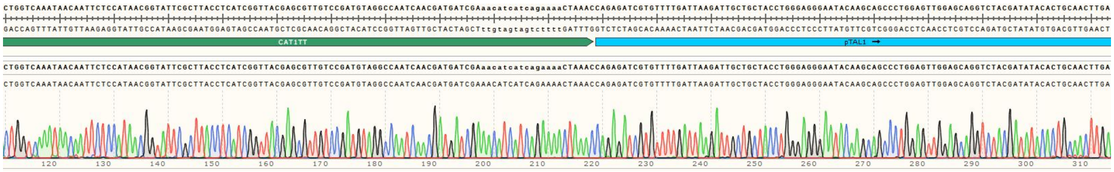

70

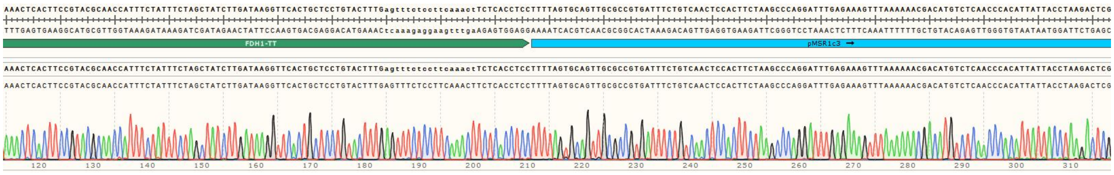

71

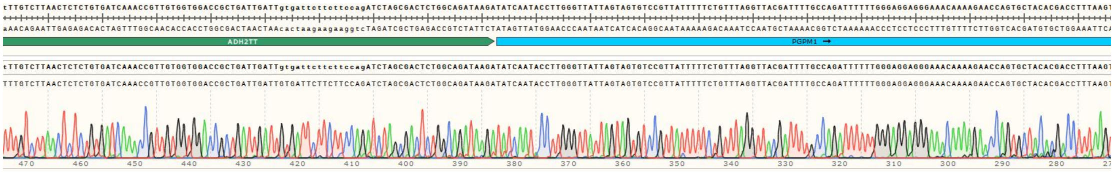

72

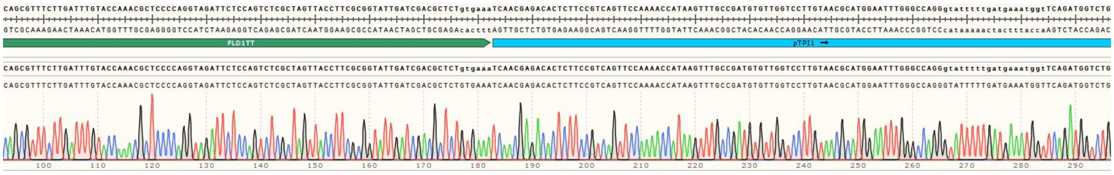

73

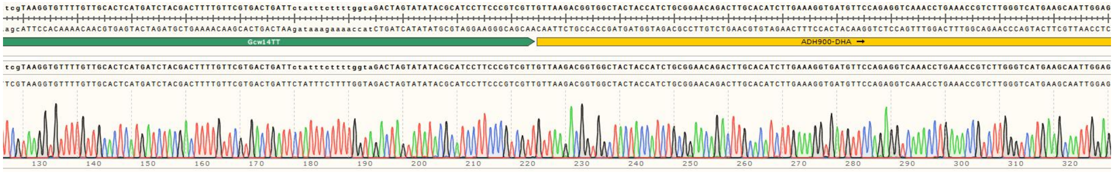

74

Figure S6. Sequencing results of 11 expression cassettes integrated into the *ADH900* loci. Because the expression cassettes and homology arms were directly amplified from plasmids and genomes, we only needed to sequence whether the correct assembly between fragments was achieved.

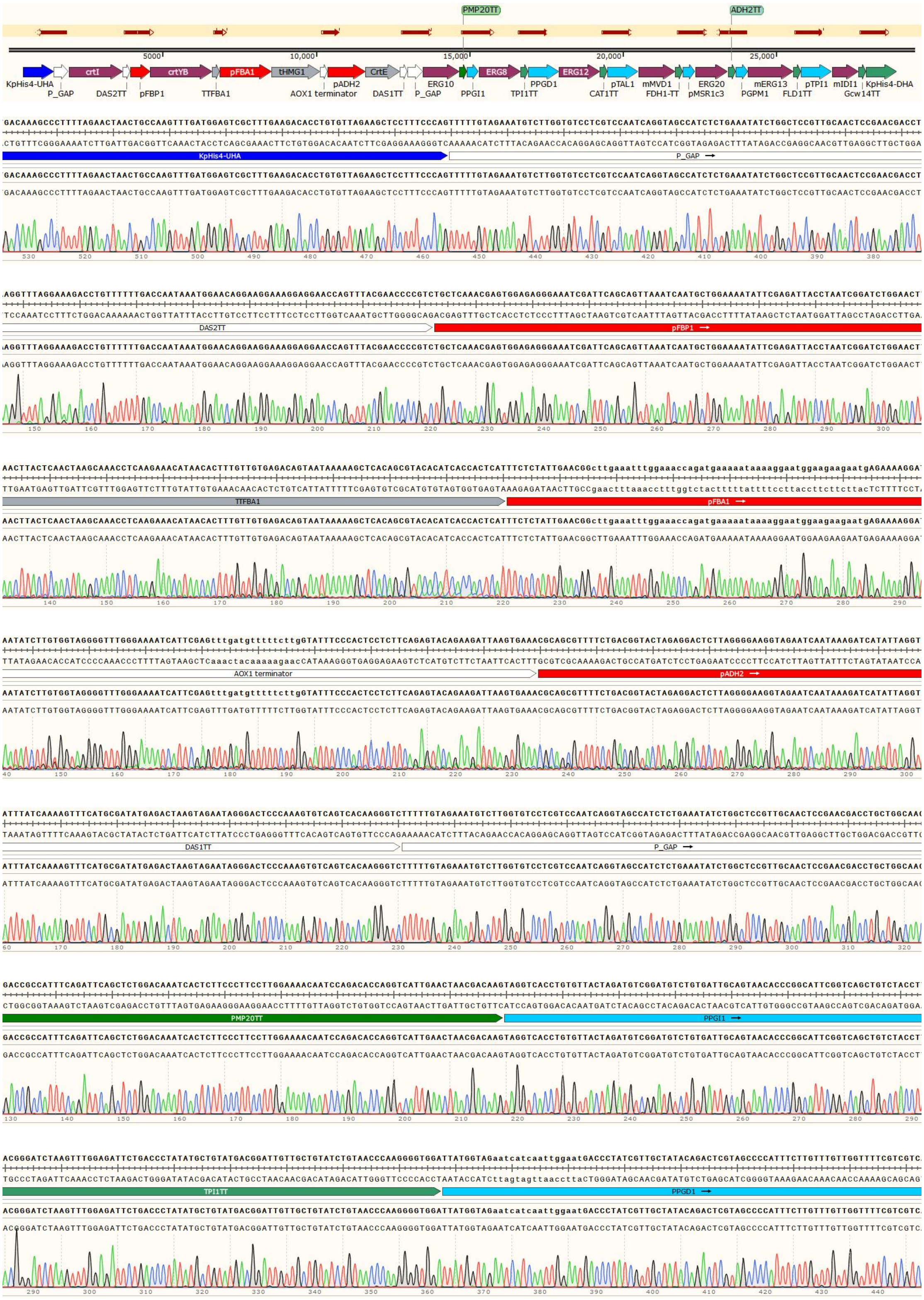

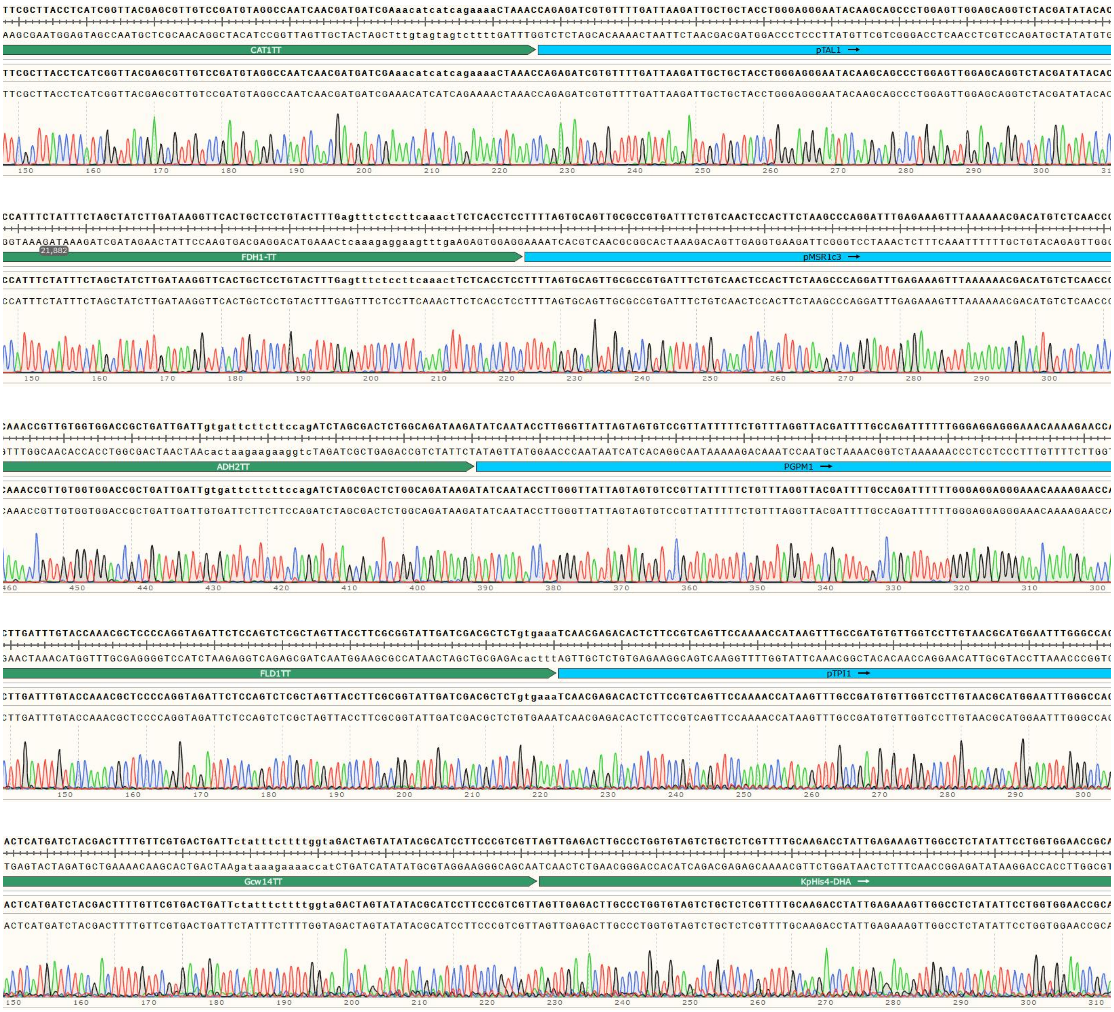

Figure S7. Sequencing results of 11 expression cassettes integrated into the *KpHis4* loci. Because the expression cassettes and homology arms were directly amplified from plasmids and genomes, we only needed to sequence whether the correct assembly between fragments was achieved.

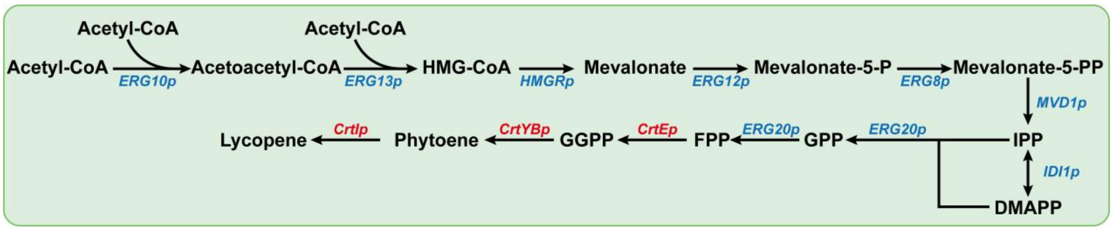

Figure S8. The schematic diagram of the lycopene synthesis pathway. Proteins marked blue exist in the endogenous MVA pathway in *K. phaffii*, and those marked red were heterologous proteins.

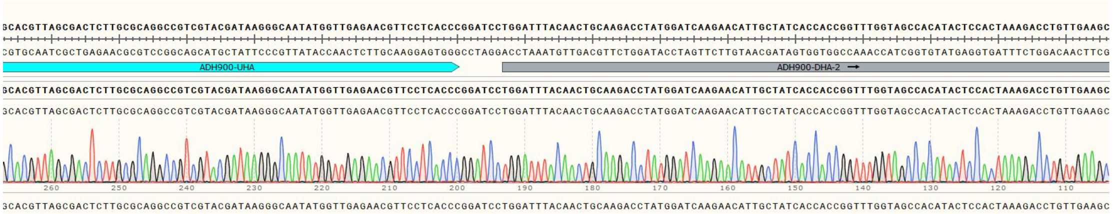

Figure S9. Sequencing results of large DNA fragment knockout by introducing two DSBs.

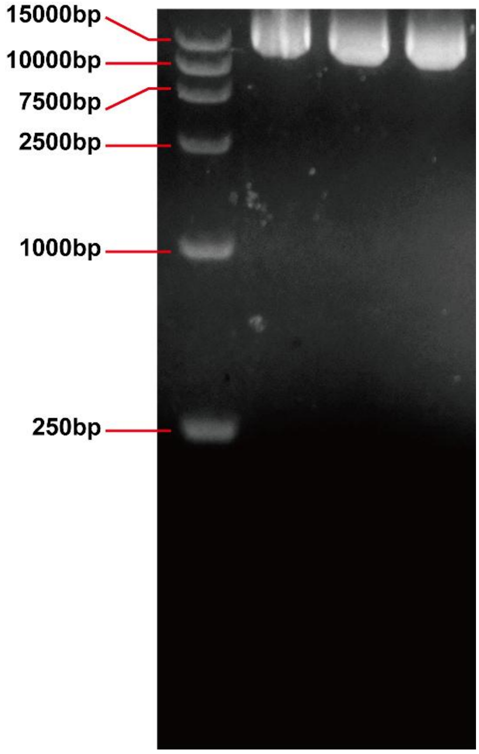

Figure S10. Gel electrophoresis result of plasmid pZW1

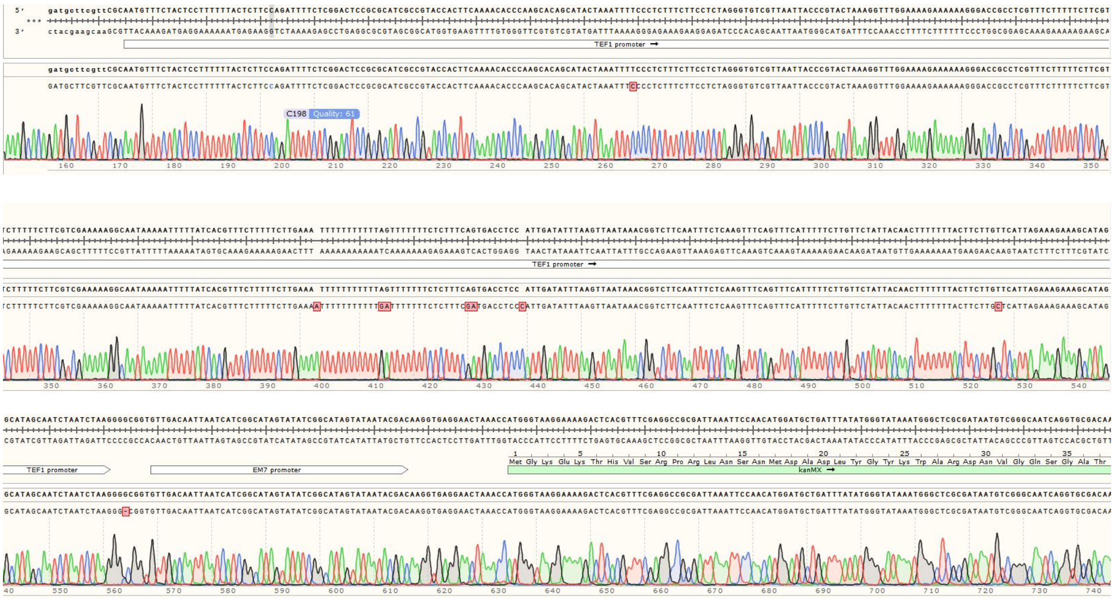

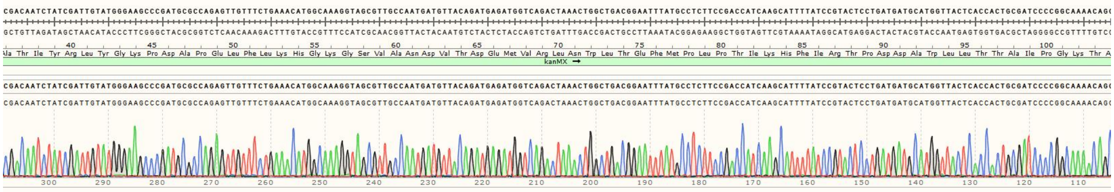

113

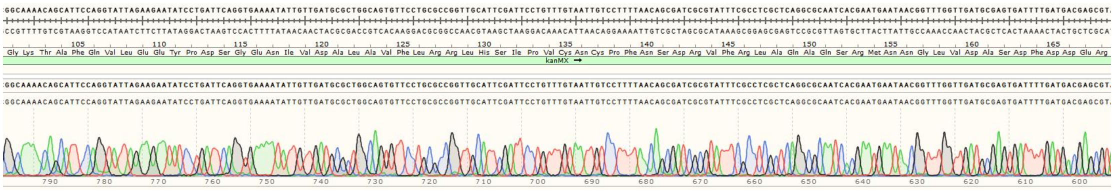

114

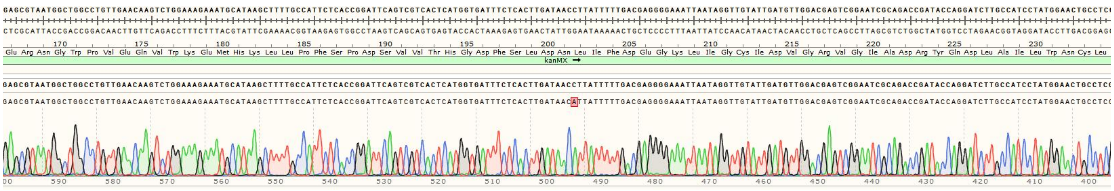

115

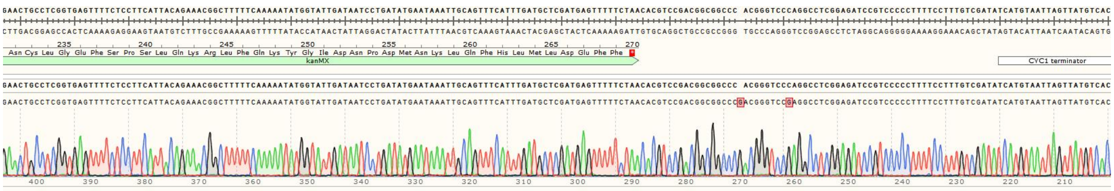

116

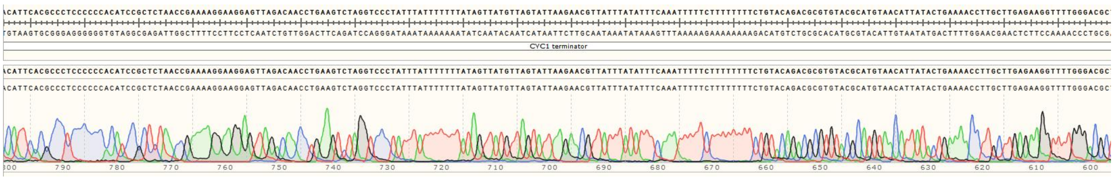

117

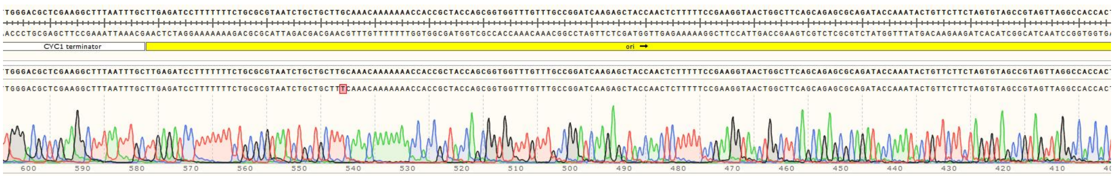

118

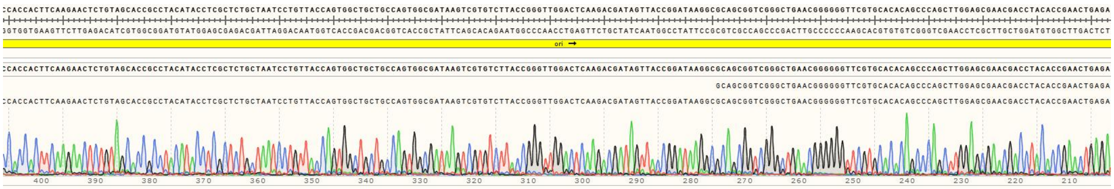

119

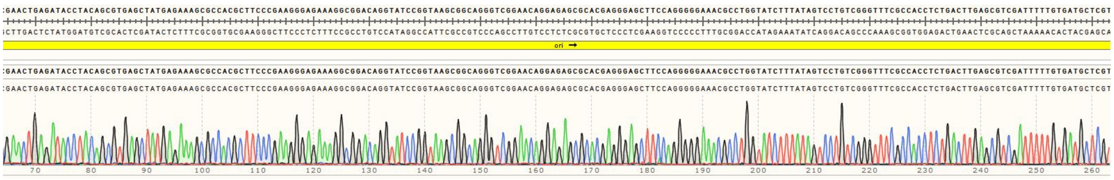

120

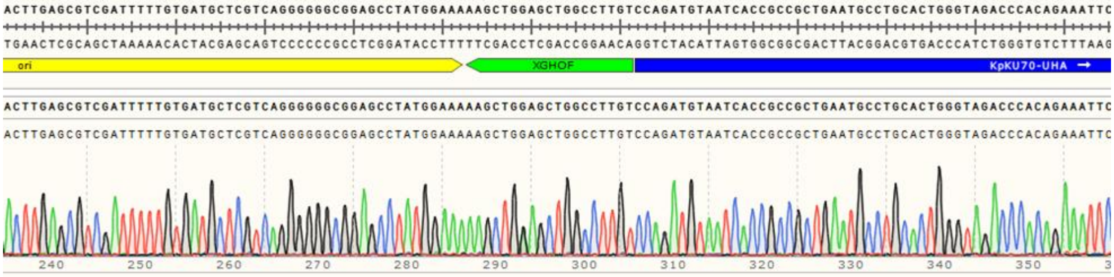

121

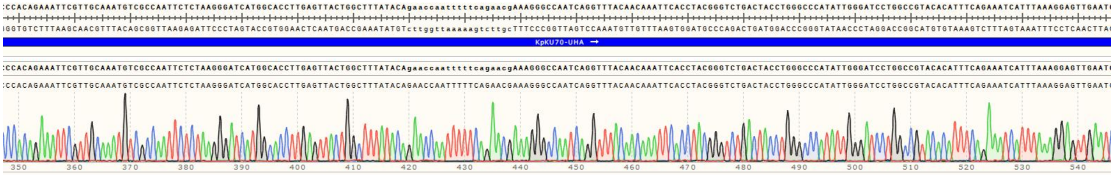

122

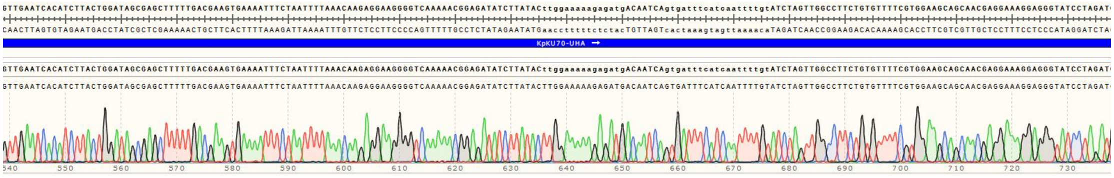

123

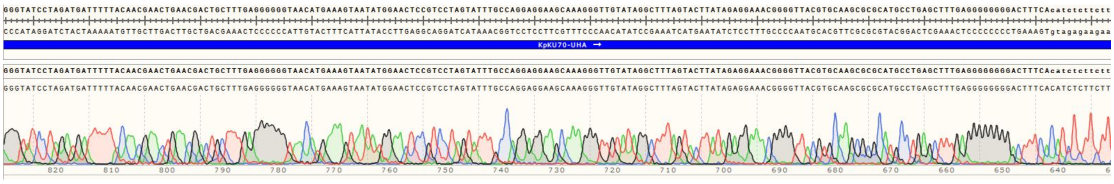

124

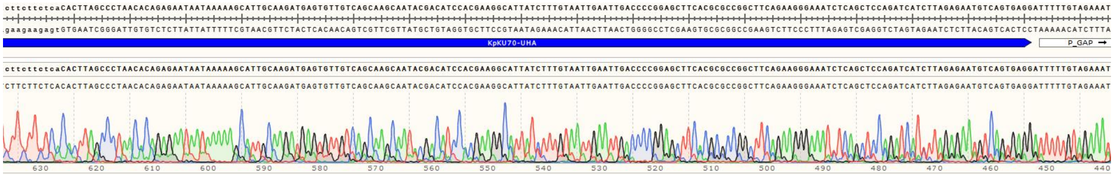

125

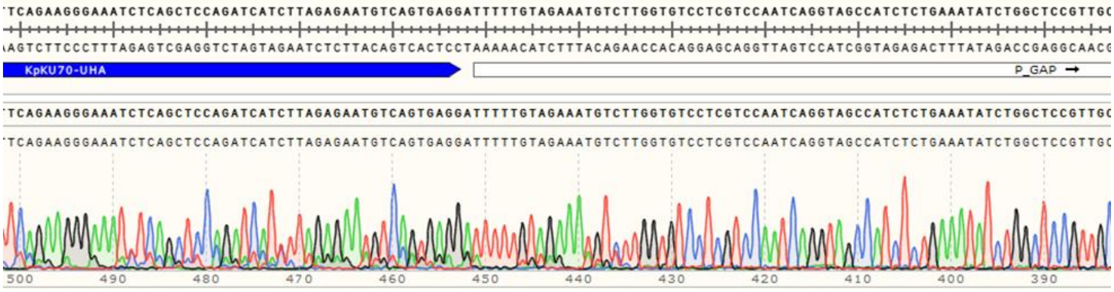

126

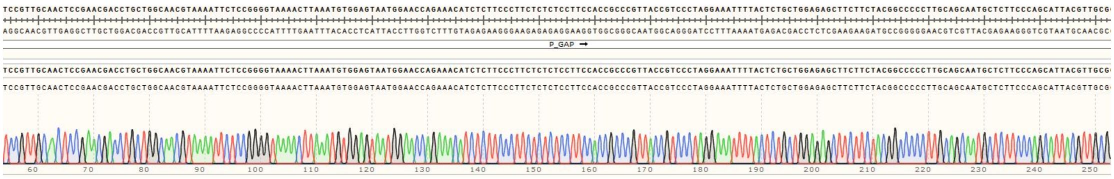

127

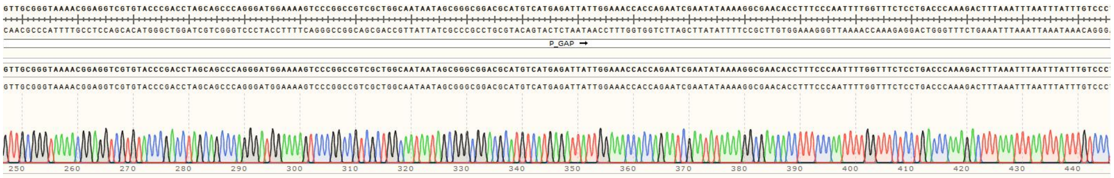

128

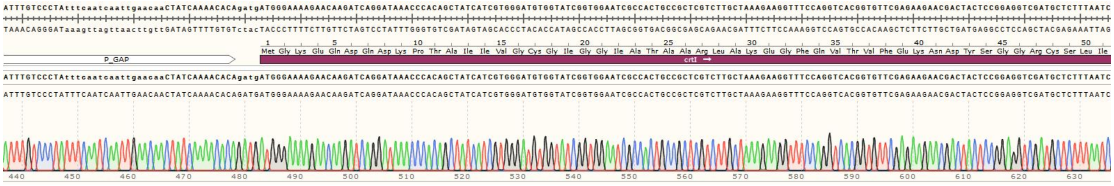

129

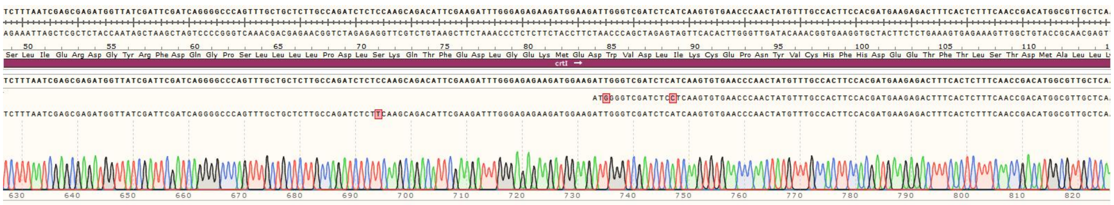

130

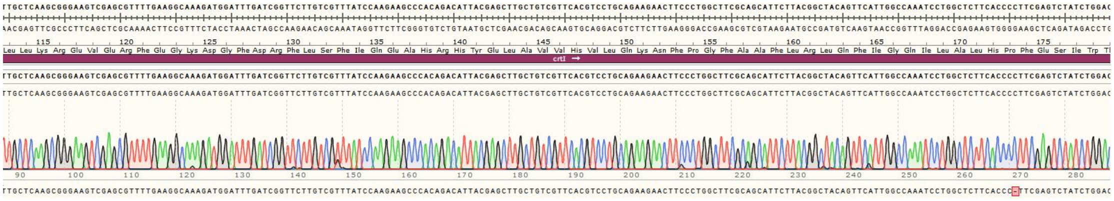

131

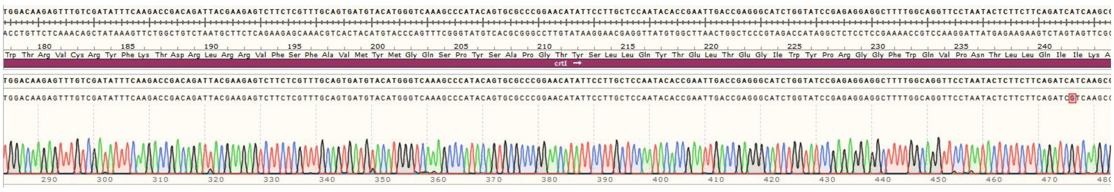

132

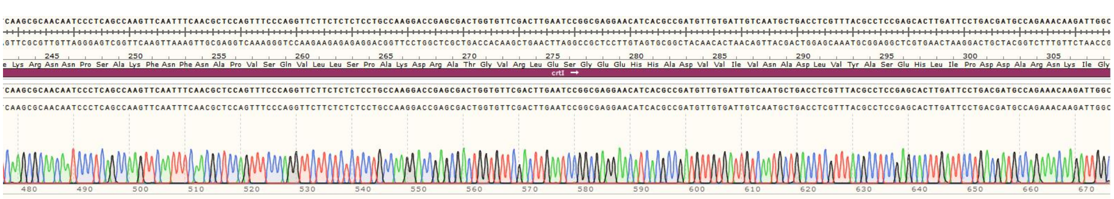

133

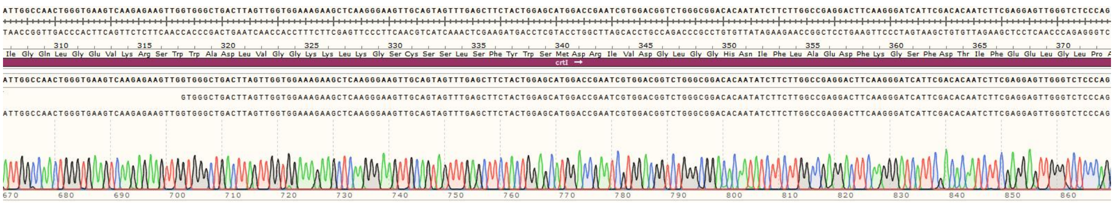

134

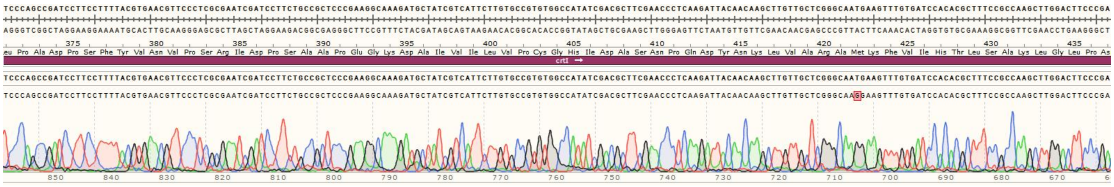

135

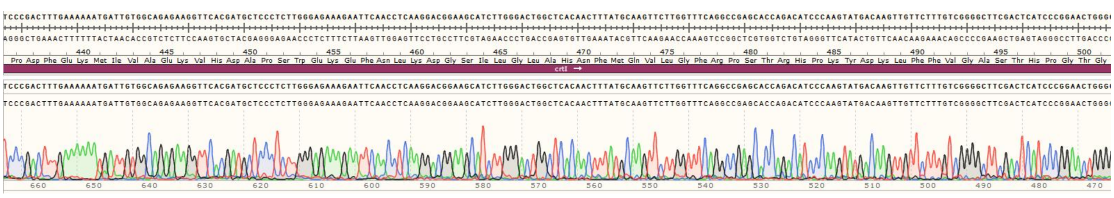

136

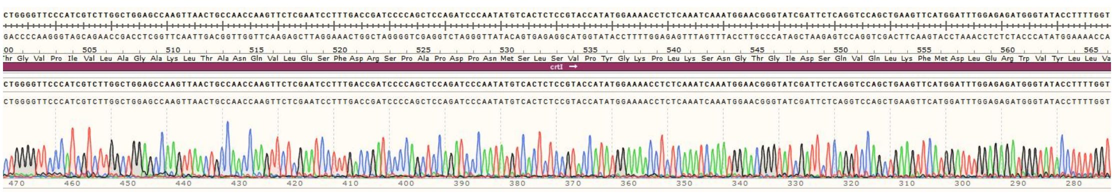

137

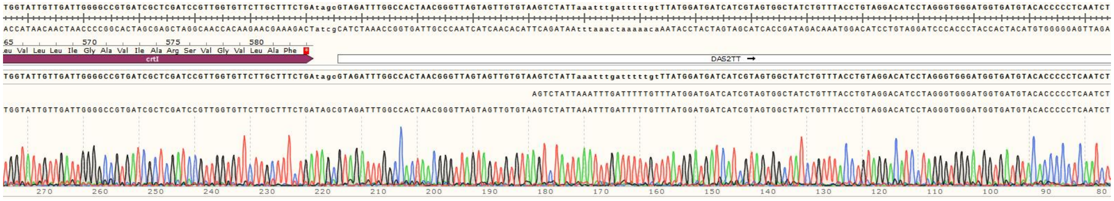

138

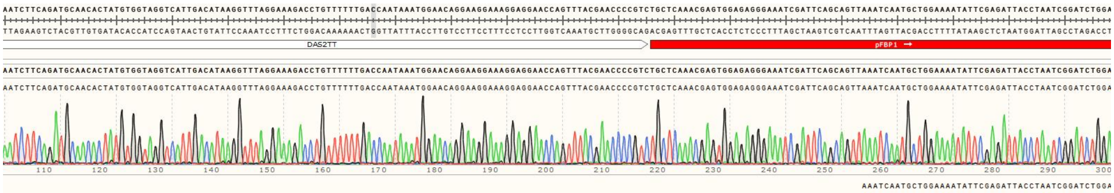

139

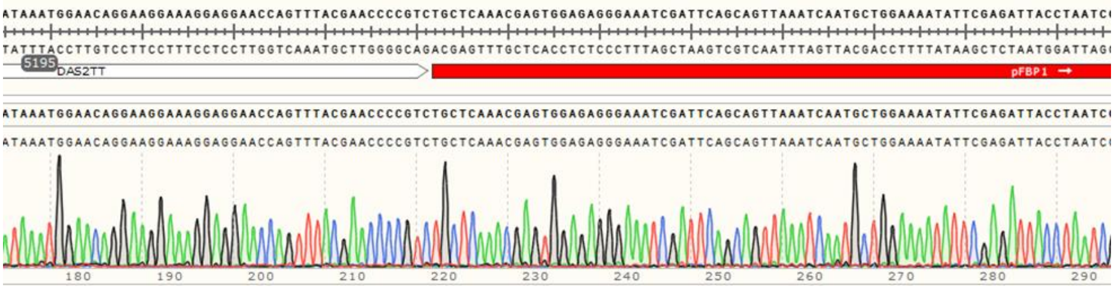

140

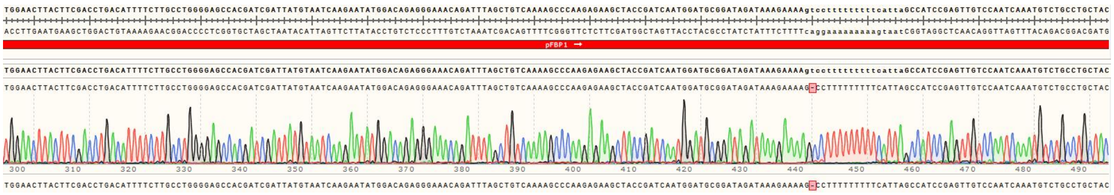

141

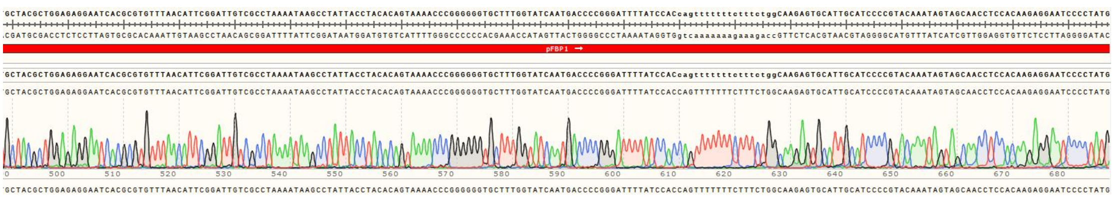

142

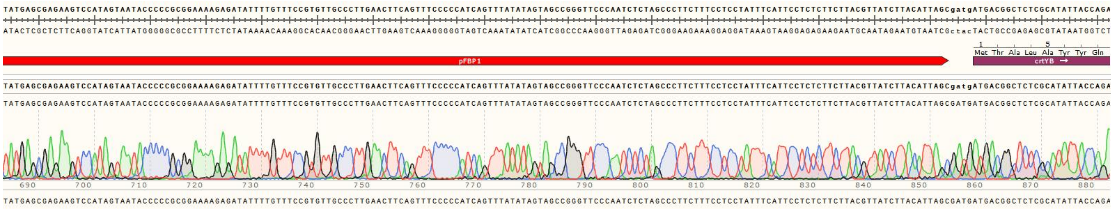

143

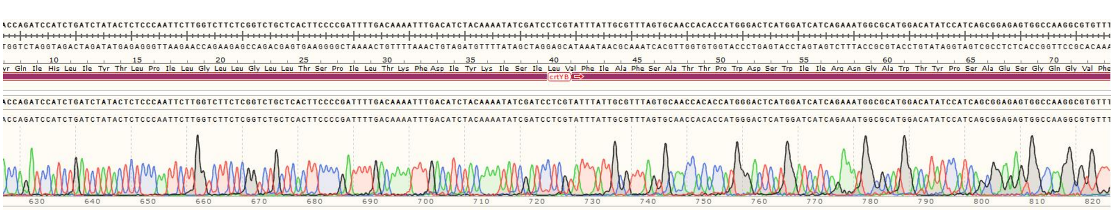

144

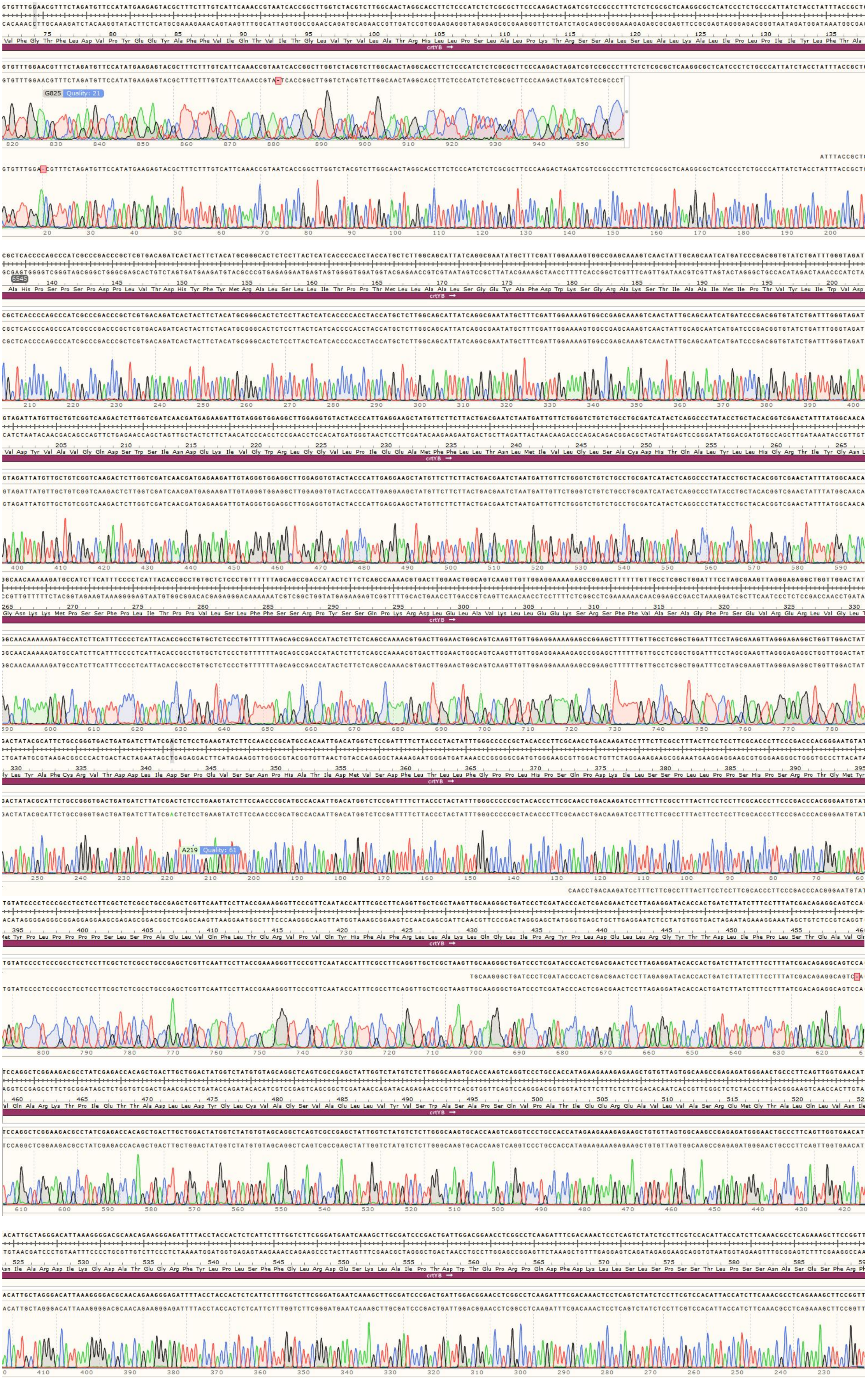

145

146

147

148

149

150

151

152

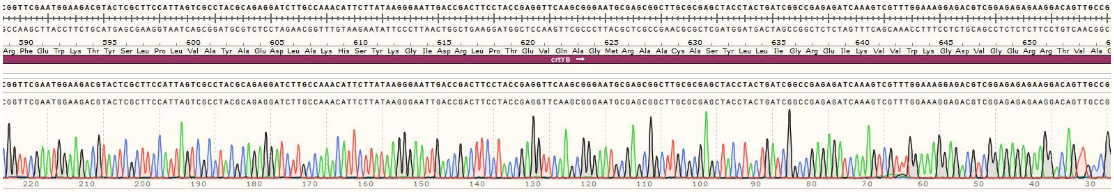

153

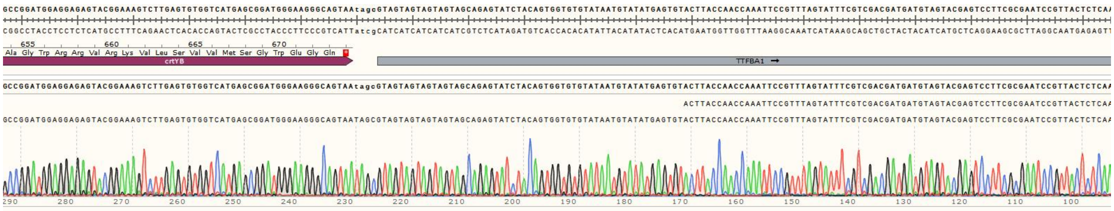

154

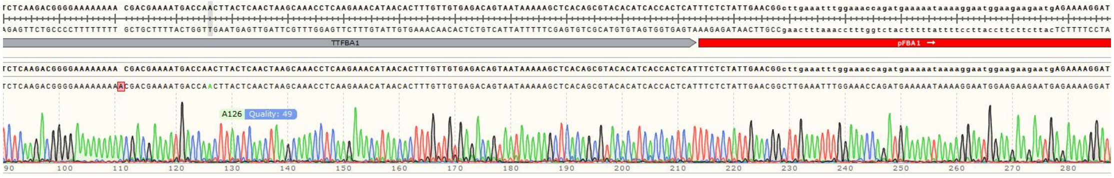

155

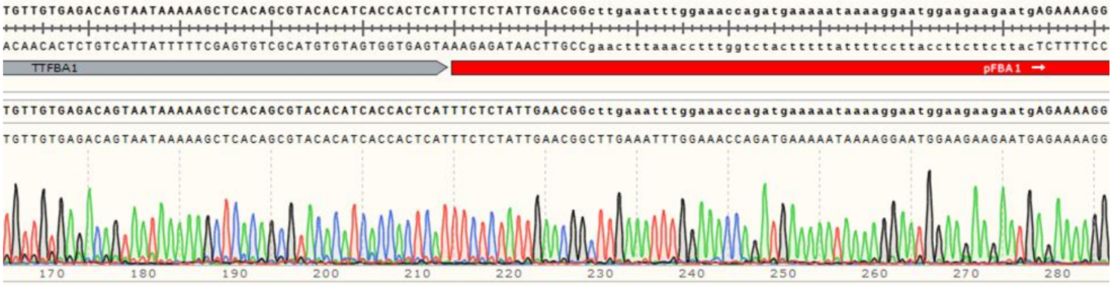

156

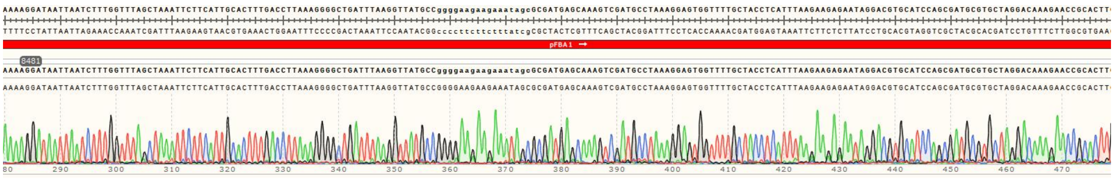

157

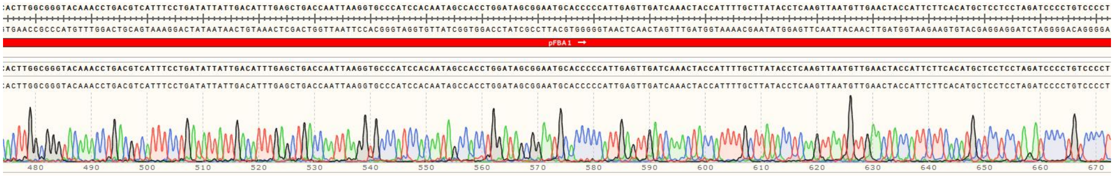

158

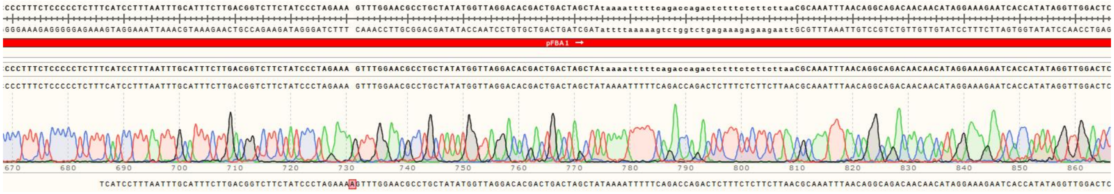

159

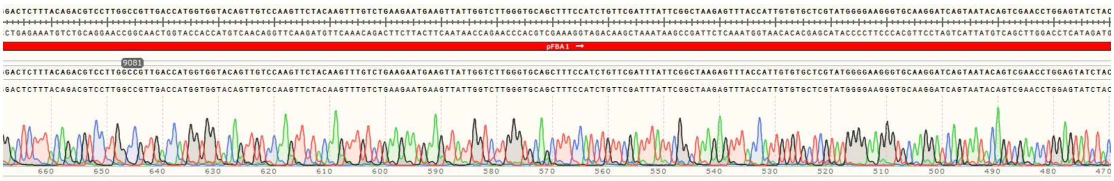

160

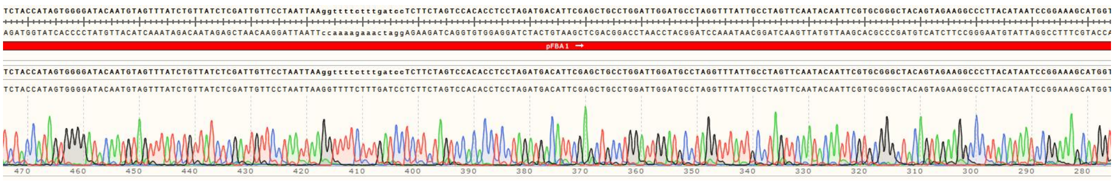

161

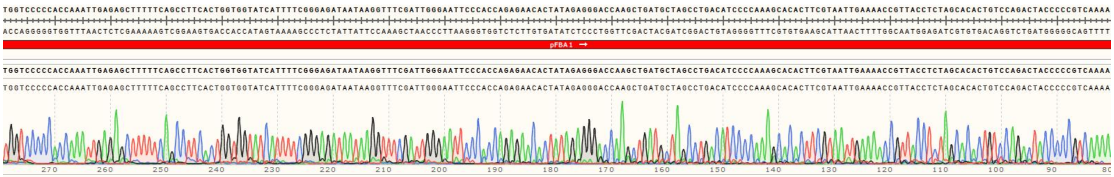

162

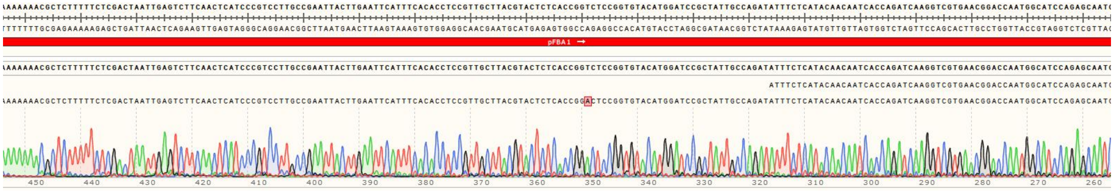

163

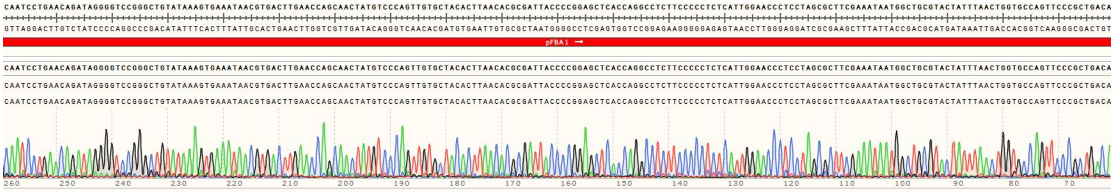

164

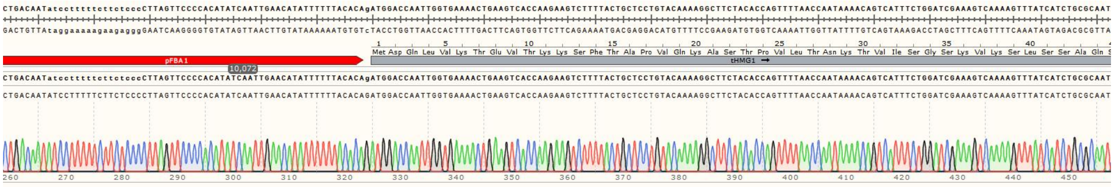

165

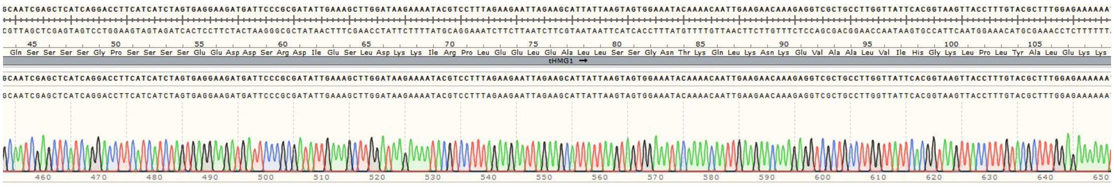

166

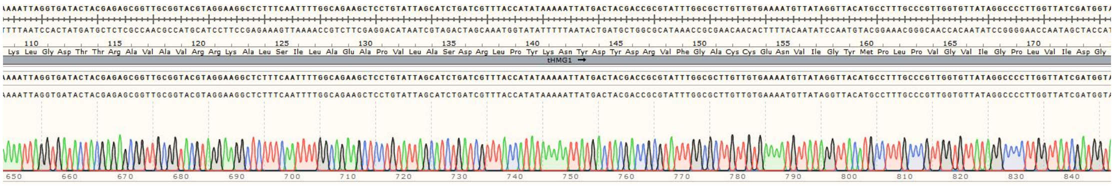

167

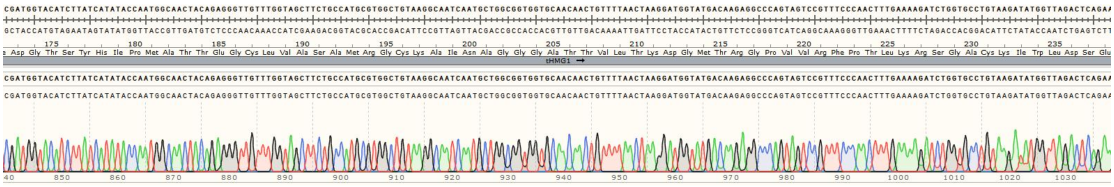

168

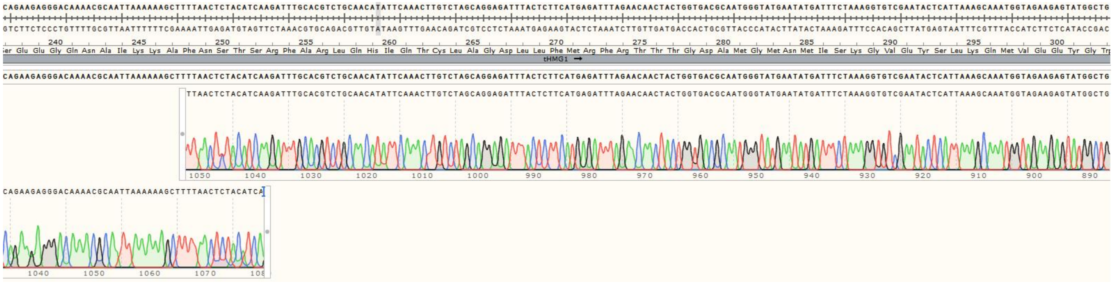

169

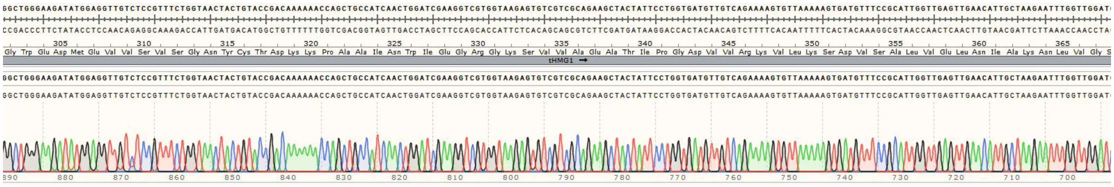

170

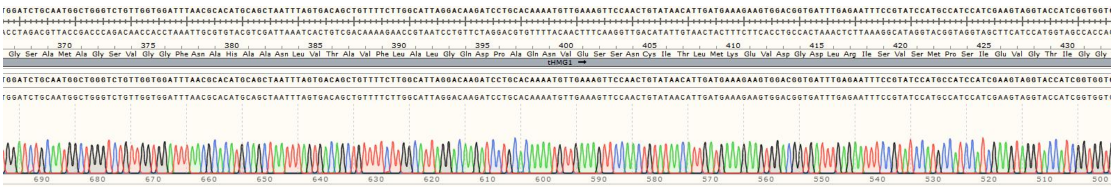

171

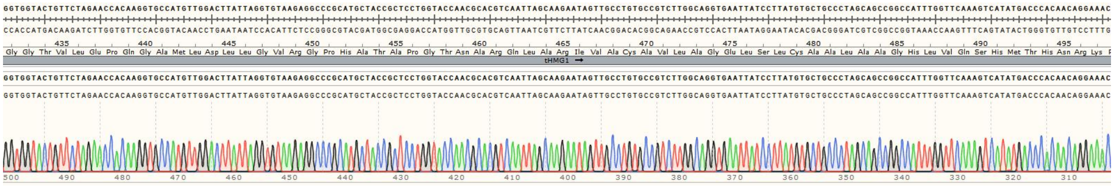

172

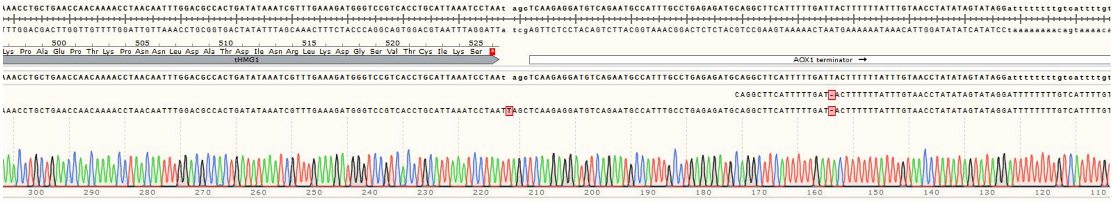

173

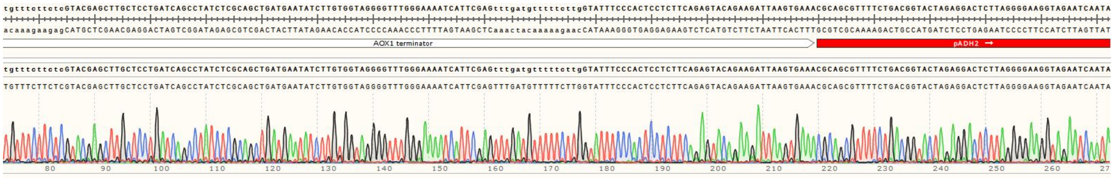

174

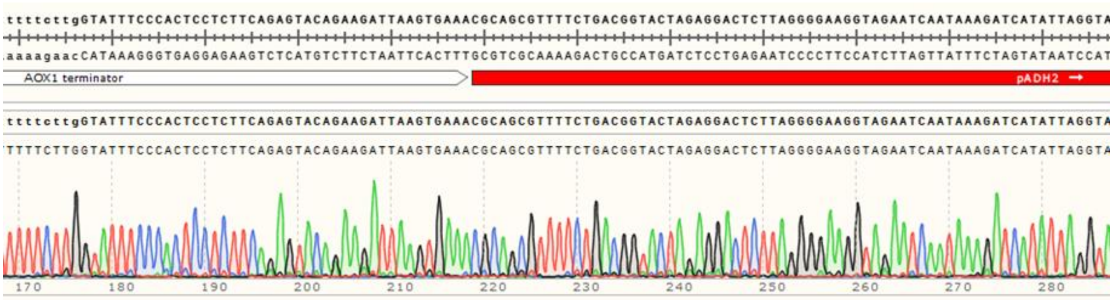

175

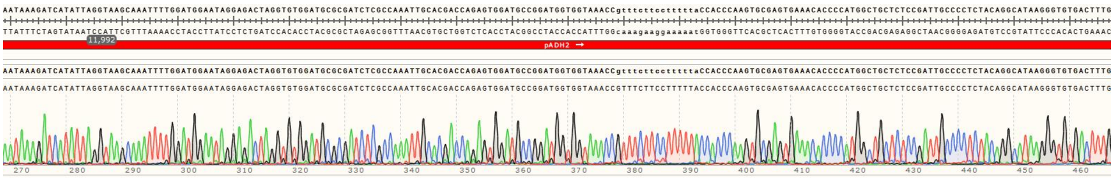

176

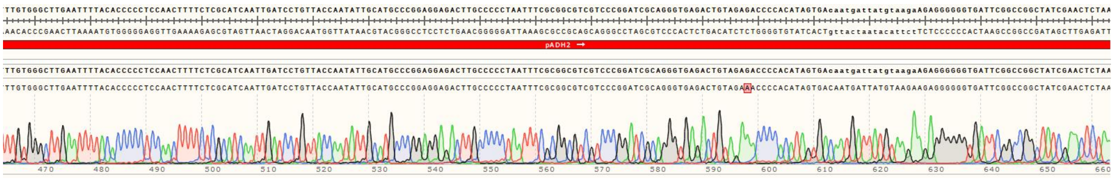

177

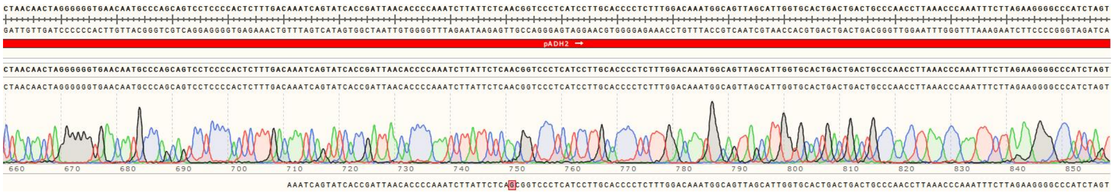

178

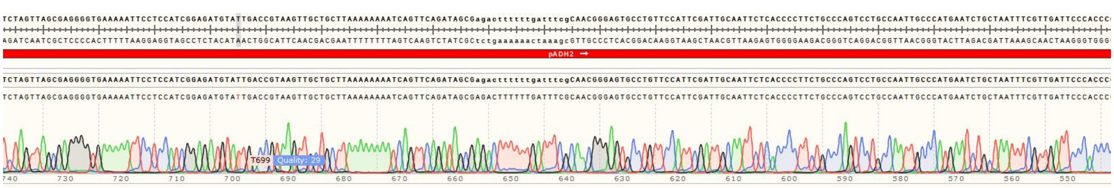

179

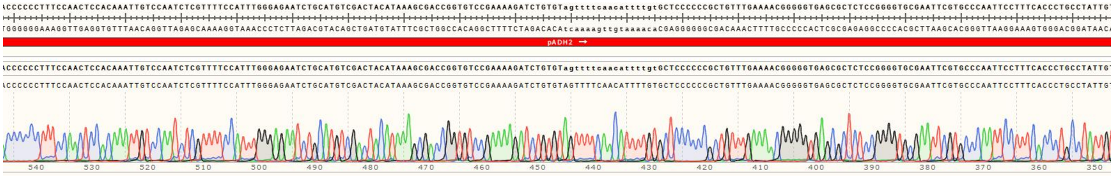

180

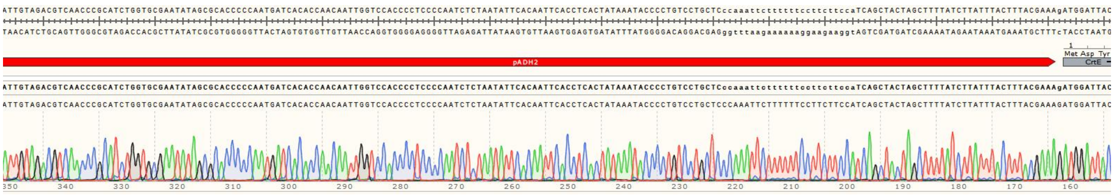

181

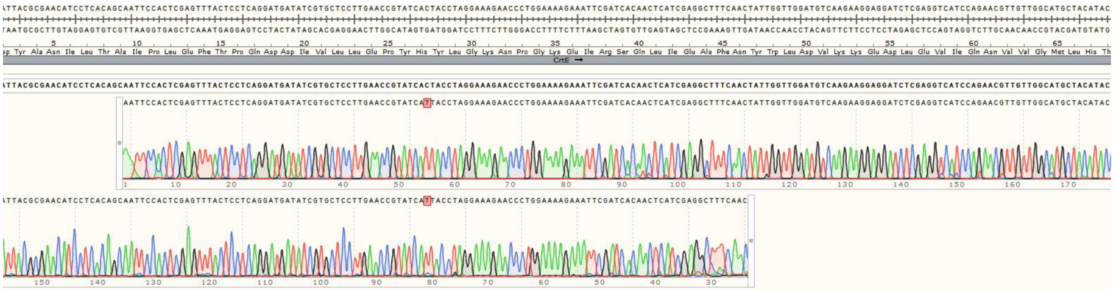

182

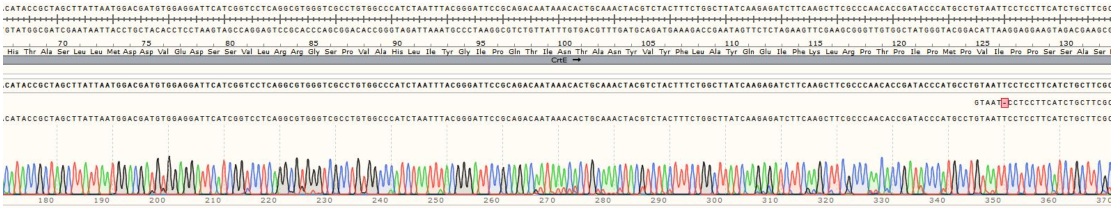

183

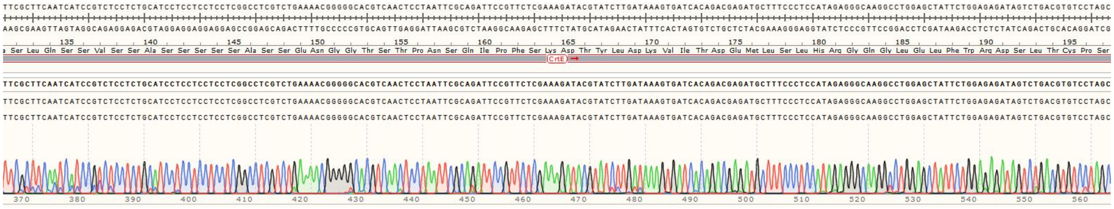

184

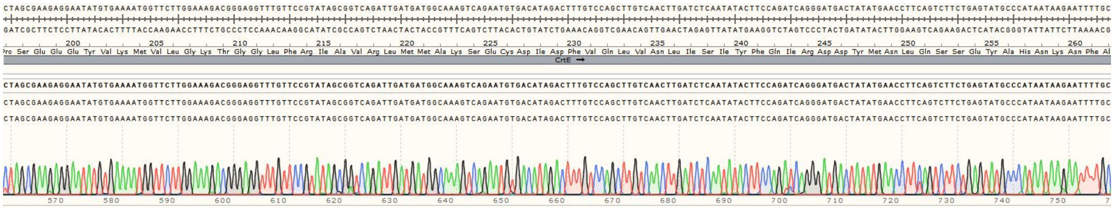

185

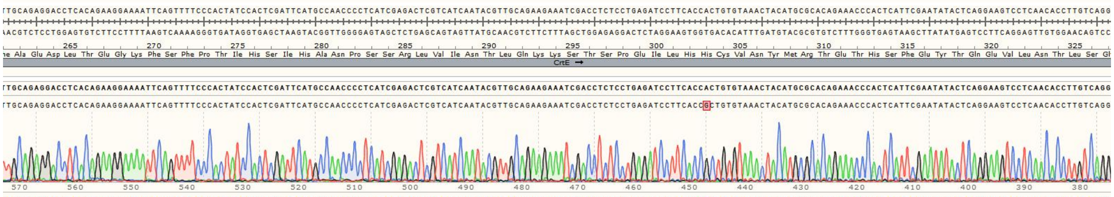

186

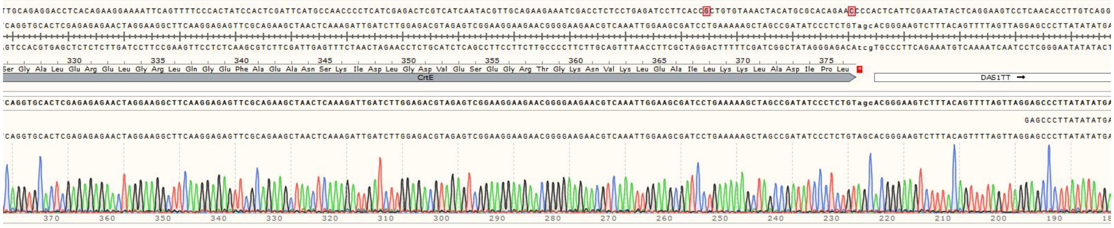

187

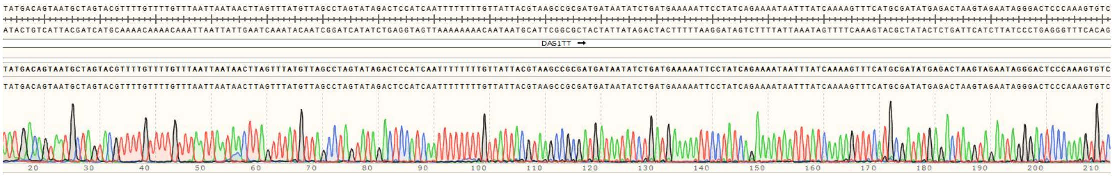

188

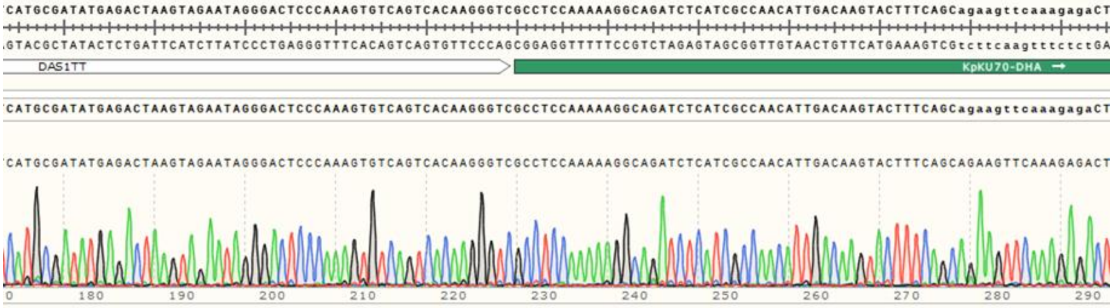

189

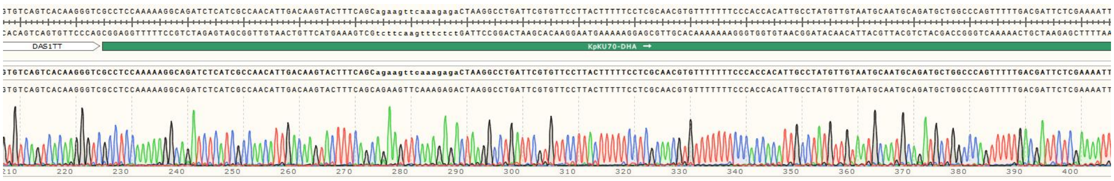

190

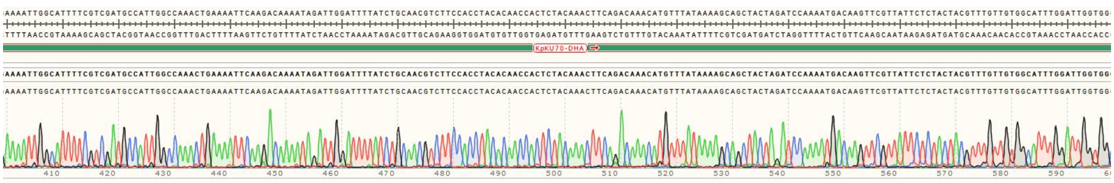

191

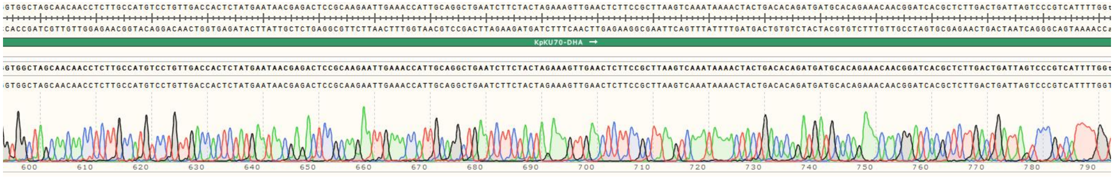

192

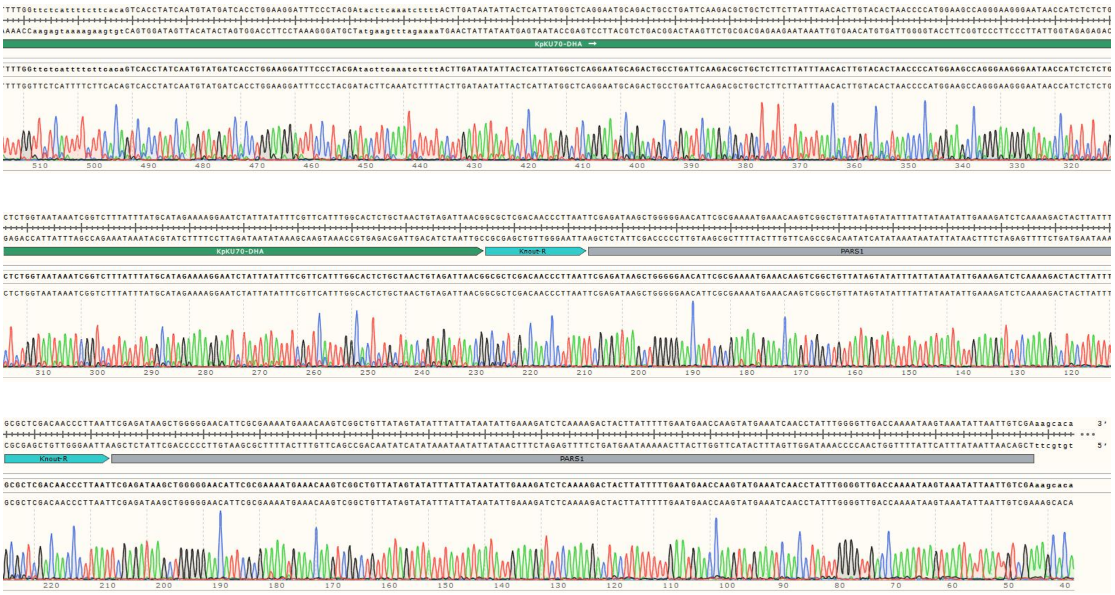

Figure S11.Complete sequencing results results of multi-fragment self-assembly into the plasmid pZW1.
